# Optimized design and analysis of preclinical intervention studies *in vivo*

**DOI:** 10.1038/srep30723

**Published:** 2016-08-02

**Authors:** Teemu D. Laajala, Mikael Jumppanen, Riikka Huhtaniemi, Vidal Fey, Amanpreet Kaur, Matias Knuuttila, Eija Aho, Riikka Oksala, Jukka Westermarck, Sari Mäkelä, Matti Poutanen, Tero Aittokallio

**Affiliations:** 1 Department of Mathematics and Statistics, University of Turku, Turku, Finland; 2 Institute for Molecular Medicine Finland (FIMM), University of Helsinki, Helsinki, Finland; 3 Turku Center for Disease Modeling (TCDM), University of Turku, Turku, Finland; 4 Drug Research Doctoral Programme (DRDP), University of Turku, Turku, Finland; 5 Cancer Cell Signaling Group, Turku Centre for Biotechnology, University of Turku, Turku, Finland; 6 Department of Physiology, Institute of Biomedicine, University of Turku, Turku, Finland; 7 Orion Corporation, Orion Pharma, Department of Oncology and Critical Care Research, Turku, Finland; 8 Department of Medical Biochemistry and Genetics, Institute of Biomedicine, University of Turku, Turku, Finland; 9 Department of Pathology, University of Turku, Turku, Finland; 10 Turku Doctoral Programme of Molecular Medicine (TuDMM), University of Turku, Turku, Finland; 11 Functional Foods Forum, University of Turku, Turku, Finland; 12 Institute of Medicine, The Sahlgrenska Academy, Gothenburg University, Gothenburg, Sweden

## Abstract

Recent reports have called into question the reproducibility, validity and translatability of the preclinical animal studies due to limitations in their experimental design and statistical analysis. To this end, we implemented a matching-based modelling approach for optimal intervention group allocation, randomization and power calculations, which takes full account of the complex animal characteristics at baseline prior to interventions. In prostate cancer xenograft studies, the method effectively normalized the confounding baseline variability, and resulted in animal allocations which were supported by RNA-seq profiling of the individual tumours. The matching information increased the statistical power to detect true treatment effects at smaller sample sizes in two castration-resistant prostate cancer models, thereby leading to saving of both animal lives and research costs. The novel modelling approach and its open-source and web-based software implementations enable the researchers to conduct adequately-powered and fully-blinded preclinical intervention studies, with the aim to accelerate the discovery of new therapeutic interventions.

*In vivo* animal studies are an essential part of any drug development project. To further increase the reproducibility and translatability of preclinical studies, there is an increasing need to improve their experimental design and statistical analysis^1^,^2^,^3^,^4^,^5^,^6^. Recurrent concerns are especially related to lack of power calculations for sample size estimation, inadequate conduction of randomized and blinded intervention group allocations, and limited consideration of individual animal characteristics at baseline prior to interventions^2^,^6^,^7^,^8^,^9^,^10^. It has been argued that preclinical animal studies should more closely follow the established practices applied in the human clinical trials, where standardized requirements have been enforced for reporting statistical power, randomization procedures and stratification factors^1^,^11^. Typical sources of variation in the animal baseline characteristics include differences in gender, body weight and age, as well as in the genetic differences, cage conditions or the variability in gut microbiota^7^,^12^,^13^,^14^. Each of these experimental factors may contribute to confounding variability in the intervention responses, leading to false positive or negative findings, unless the study is carried out using adequate sample sizes or design that normalizes such confounding factors. Although these issues are widely acknowledged among the researchers, and guidelines are available for standardizing and reporting preclinical animal research^15^, the implementation of the best practices is often neglected^2^,^8^,^16^,^17^,^18^,^19^. Accordingly, a recent survey revealed that over 85% of published animal studies did not describe any randomization or blinding, and over 95% lacked the estimation of sufficient sample size needed for detecting true effects in the intervention studies^17^.

In the absence of established practices and procedures for power calculations tailored for preclinical studies, the preferred sample size is often decided through historical precedent rather than solid statistics^9^,^20^. Similarly, the current approaches for allocating animals to separate intervention arms are typically based on manual picking and balancing of the animal groups based on only one baseline variable^6^. However, such simple design procedures may easily miss the complex relationships between multiple baseline variables, and the subtle intervention effects. Further, it remains a challenging question how to choose among the multiple baseline markers due to inherent differences in animal experimentation. Preferably, the intervention groups should be balanced using all the available baseline factors, including information about the animal characteristics (e.g., gender, age and weight), littermates, housing conditions, and pre-treatments, among others. Otherwise, even minor uncontrolled differences between the treatment arms may cause significant variation in the response profiles^13^. Many of the experimental factors lead to complex hierarchical designs, with nested animal, host-tumor, cage, batch and litter relationships at multiple levels, thus reaching beyond the capability of the existing randomization and allocation methods available for preclinical animal studies^21^,^22^. The current methods often assume the independence of the baseline variables and experimental units, which may lead to over-optimistic evaluation of the effective sample size, also known as pseudo-replication^16^. This takes place, for instance, when one allocates multiple animals from a single batch or cage to a single treatment arm, or when multiple tumours are placed in the same animal.

## Results

**Figure 1:**
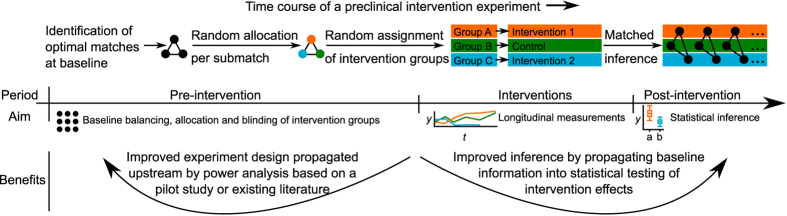
Benefits of the modelling framework over the course of the study period. The animal baseline matching improves the statistical analysis and design of preclinical animal studies in terms of power calculations, balanced allocations, and intervention blinding (pre-intervention period), as well as through the use of matching information in the statistical testing of the intervention effects (post-intervention period).

We developed and implemented a novel methodology to improve the experimental design and statistical analysis of preclinical studies carried out with experimental animals. The advances are based on a mathematical optimization framework for animal matching that improves both the unbiased allocation of the intervention groups, as well as the sensitivity and specificity of the post-intervention efficacy evaluations by making the full use of all the available baseline characteristics. To support its widespread use in various experimental settings, the modelling framework has been made available both as an open-source R-package (http://cran.r-project.org/package=hamlet) (Supplementary Note S1), and through a web-based graphical user interface (http://rvivo.tcdm.fi/) (Supplementary Note S2). To our knowledge, these implementations are the first that effectively consider the nested, hierarchical structures of preclinical animal studies across the different phases of the experiment, starting from the power analyses, to allocation of animals to the various treatment arms, and all the way to finally evaluate the intervention effects (Fig. 1). In the present work, we demonstrate the benefits of these tools over conventional analysis in two applications of orthotopic xenografts of VCaP prostate cancer cells in immune deficient mice as disease models for castration-resistant prostate cancer (CRPC) (Supplementary Fig. S1). The first study analysed the efficacy of two androgen receptor antagonists (ARN-509 and MDV3100) to suppress the growth of castration-resistant VCaP tumors^23^, while the second study investigated the effect of surgical and pharmaceutic therapies on orchiectomized mice (for details; see Supplementary Methods and Supplementary Note S3).

**Figure 2:**
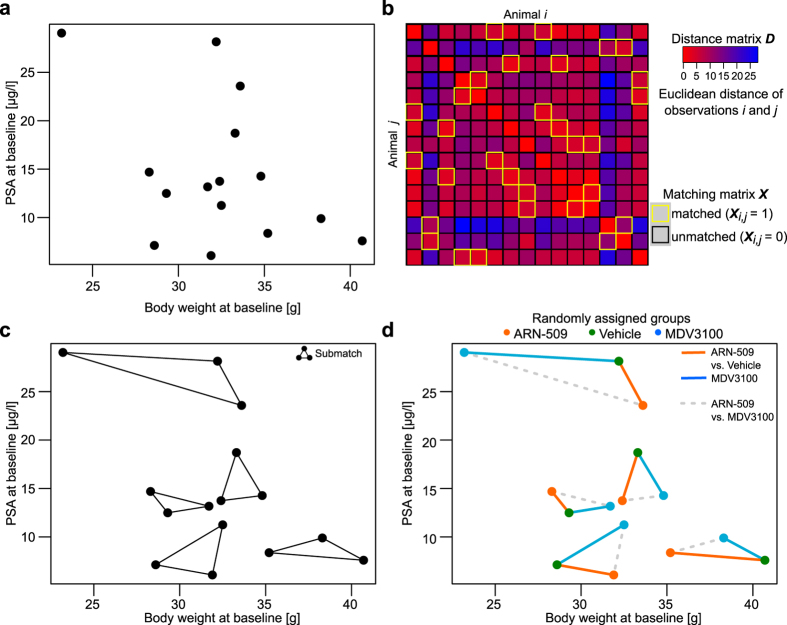
Optimal matching of animals in the case of orthotopic VCaP mouse xenografts. The original task was to randomly assign 75 animals into five balanced intervention groups (one control and four treatment groups, each consisting of 15 animals), but here we focus on two of the treatments only (ARN-509 and MDV3100), using a sub-sample of the complete data matrix (see Supporting Fig. S3). (**a**) Bivariate observations sampled from the VCaP study, illustrating the two selected baseline variables (body weight and PSA). (**b**) 15 × 15 dimensional distance matrix ***D*** calculated based on the baseline variables was used as an input to the matching procedure, which solves the optimal animal matching matrix ***X***. (**c**) The optimal submatches from the branch and bound algorithm, which guarantees a globally optimal solution (see Supporting Fig. S7). (**d**) The optimally matched animals were randomized into the intervention groups via blinded treatment label assignments (coloured points). The baseline matching information was used in the statistical testing of the treatment effects, mainly through paired comparisons between the treated and control animals (solid lines). Alternatively, the model also allows for direct comparisons between the two treatments (dotted lines).

In a given pool of animals, the matching solution provides an optimal intervention group allocation of animals (or tumors) based on several baseline characteristics (Fig. 2). Rather than considering only the optimal pairing of individual animals, the solution can be used also to identify optimal matches among a number of features, animals or tumors, e.g., triplets, quadruplets, or more (see Methods for the mathematical formulation of the matching problem). Such optimal combinations, referred here to as *submatches*, are constructed by minimizing the sum of all the pairwise distances between the members of each submatch, illustrated here by pairwise connecting edges (Fig. 2). Since the non-bipartite matching procedure does not require pre-defined group labels, the control group can be selected without any guidance from the experimenters (Supplementary Fig. S7b). Instead, the animal allocation is performed objectively within each submatch by distributing its members randomly to separate treatment arms, hence enabling fully-blinded intervention group allocation through separate matching and randomization phases (Figs 1 and 2c,d). In the present study, we demonstrate how the matching information does not only improve the pre-intervention design, such as baseline animal group balancing and allocation, but it also improves the post-intervention statistical power to detect true treatment effects.

### Matching normalizes baseline variability in confounding variables

The first VCaP xenograft case study was originally conducted based on the matching procedure^23^, where it showed its added value in complex designs with batch/cage effects and multiple treatment groups (*n* = 15 animals per group). While the full matching included four baseline variables, we illustrate the methodology first using two key animal characteristics (PSA and body weight at baseline; Fig. 2a–c). The optimal submatches were subsequently randomized and blinded for the experimenters to enable unbiased analysis across three intervention groups (ARN-509, MDV3100 and Vehicle) (Fig. 2d). The confounding variability from the two castration batches was normalized by treating these as two separate optimal matching problems (Supplementary Fig. S3a), which guaranteed that the two batches were allocated uniformly to the intervention groups through the use of submatches (Supplementary Fig. S3b). Notably, the matching distance matrices at baseline were also significantly correlated with the post-intervention RNA-seq profiling of a randomly chosen subset of individual tumours (*p* = 0.039, Mantel’s test, *n* = 4 animals per group; Supplementary Fig. S8c), suggesting that major trends in the characteristic baseline differences used in the animal allocation were still captured by their genome-wide transcriptional responses even after the interventions (Supplementary Fig. S8a,b).

To more systematically study the degree of confounding variability and its effects on the animal allocation, we tested the frequency of statistically significant differences in all the available baseline variables between the randomized treatment groups. A total of *n* = 100,000 animal allocations were simulated either totally at random (*unmatched randomization*) or using the matching information from the optimal submatch allocations (*matched randomization*). The baseline variables considered in the optimal matching were body weight and PSA at baseline, as well as PSA fold-change from previous week prior to allocation. With the unmatched randomization, 13.8% of the treatment groups represented significant differences with respect to at least one of the baseline variables (*p* < 0.05, one-way ANOVA). In contrast, only 0.018% of the treatment groups in the matched randomizations showed any baseline differences. This indicates that matching effectively eliminates baseline differences in the confounding variables, which unless carefully controlled during the allocation process, may contribute to the poor reproducibility of preclinical research findings^24^.

### Matching improves the statistical inference of treatment responses

**Figure 3:**
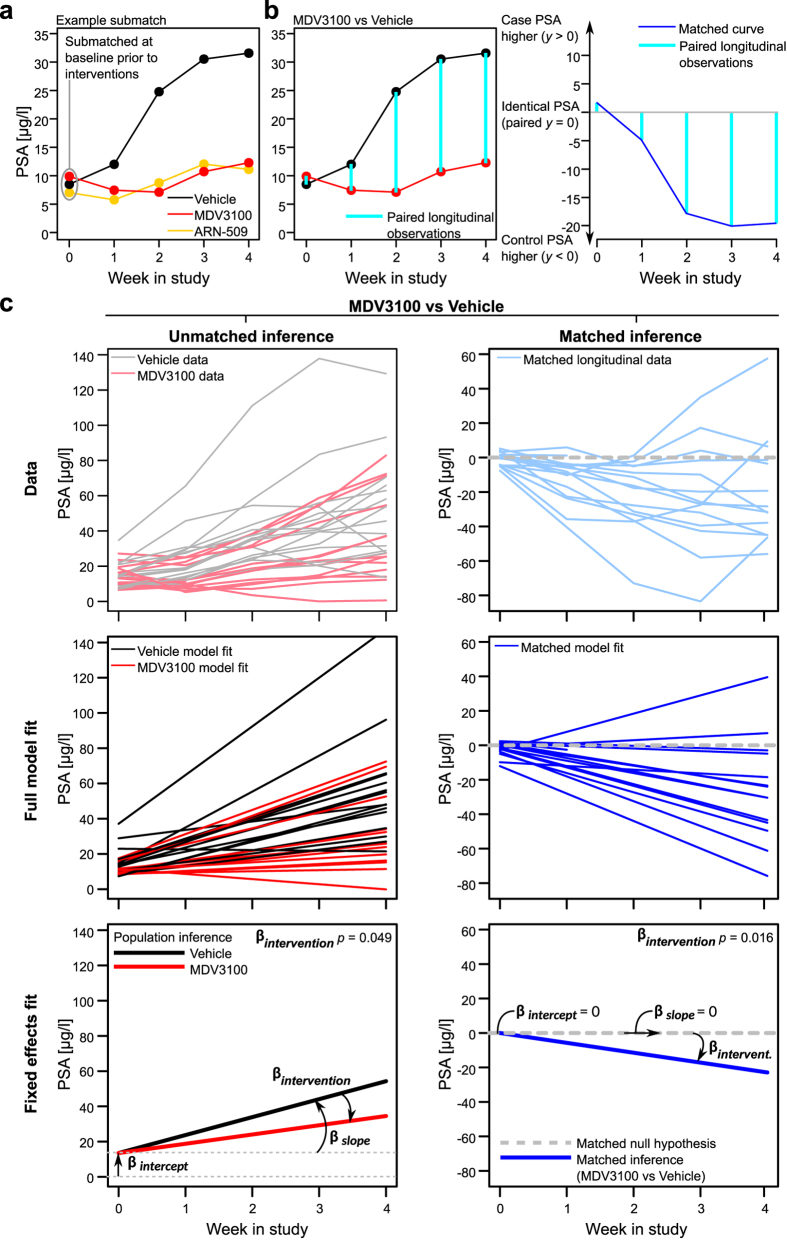
Statistical testing of the treatment effects using pairwise matched inference. (**a**) The matched inference makes use of the baseline matching information when testing the intervention effects by pairing the observed responses according to the optimal submatches at equal time points. (**b**) An example of the submatch-based pairing in the MDV3100 vs vehicle comparison, where the example trajectory was previously shown as a single estimate value in the original study^23^. Complex response differences are better captured when additional baseline information is incorporated into the statistical inference. The paired differences from the longitudinal observations (left panel) construct a single treatment curve for the pairwise matched mixed-effects modelling (right panel). (**c**) Comparison of the matched and unmatched statistical inference approaches in the MDV3100 vs vehicle comparison. Even if both inference approaches yield rather similar conclusion about the possible intervention effects, the matched approach improves the sensitivity of the detection (right panel). Different aspects of the mixed-effects modelling are visualized based on the observed data (top panel): the full model fit combining both the random and fixed effects (middle panel), and the population inference depicting only the fixed effects along with their interpretation (bottom panel). In the matched inference, the population of paired differences in the intervention effects (***β***_***intervention***_) is tested against a null hypothesis of no paired differences (*y* = 0 line). The statistical inference results of the intervention effects are summarized in Table 1, and the full model fits for the four treatment cases are shown in Supplementary Figs S5 and S6.

In the post-intervention analysis, we studied the benefits of using the matching information in the mixed-effects modelling of the treatment effects (see Methods for the model formulation), focusing first on the ARN-509 and MDV3100 treatments (Fig. 3a). The matched inference approach models the paired longitudinal differences in the intervention responses (PSA in the VCaP xenografts; Fig. 3b), based on the optimal submatches of the animals at baseline (Fig. 2c; Supplementary Fig. S3). The benefits gained by such matching-based paired testing became more evident with the MDV3100 case, where we observed that the animal body weight at baseline was inversely associated with the final PSA level (correlation coefficient *ρ* = −0.607, *p* = 0.021, Supplementary Fig. S2d). Such multivariate, longitudinal relationship between the baseline variables and treatment responses cannot be captured by the conventional, unmatched model, leading to reduced statistical sensitivity (Fig. 3c, left). The MDV3100 treatment effect became clearly significant when the baseline matching information was incorporated into the mixed-effects modelling (Fig. 3c, right). The more apparent ARN-509 intervention effect was detected both with the matched and unmatched statistical models (Table 1). Of note, the non-matched approach also benefitted here from the matched randomization of the original study^23^.

As another case study, we randomly allocated 100 VCaP mice using the matching algorithm into six intervention groups (Supplementary Fig. S4), out of which three are further investigated here (Control, orchiectomized (ORX) and ORX+Tx). As was expected, when compared to the intact control animals, both the matched and unmatched statistical models were able to detect the significant intervention effect from the ORX surgery (Table 1). However, the unmatched approach totally missed the additional effect from an undisclosed pharmaceutic treatment (Tx), while the ORX+Tx combination effect was found significant after using the baseline matching information in paired testing of the longitudinal intervention responses. In the combination case, the standard, non-paired analysis lacked the power to distinguish the complex response patterns between the intervention groups, in part due to the non-linear responses in the early time points (Supplementary Fig. S6). In contrast, the paired inference, enabled by the matching information, was able to capture these pairwise response differences, leading to subtle yet significant intervention-specific effect sizes (Table 1). These results support the improved statistical sensitivity gained by the baseline matching information in the detection of true treatment effects, especially when studying more complex and subtle intervention effects.

### Matching increases statistical power to detect true treatment effects

Since the intervention effects in the preclinical studies are often relatively subtle, statistical power calculations are critical for estimating the sufficient number of animals needed to detect a true effect. However, preclinical experiments pose specific requirements for the power calculations, due to the complex nature of longitudinal responses, relatively high frequency of missing values originating from animal health or other exclusion criteria, complex hierarchical designs, as well as multivariate baseline characteristics, which are beyond the capacity of any standard sample size estimation procedures. We addressed the above mentioned challenges by implementing a model-based power analysis calculation. The method first samples animals with replacement from an estimated mixed-effects model, and then uses these bootstrap datasets to re-estimate the specified statistical model (see Methods for the modelling details).

**Figure 4:**
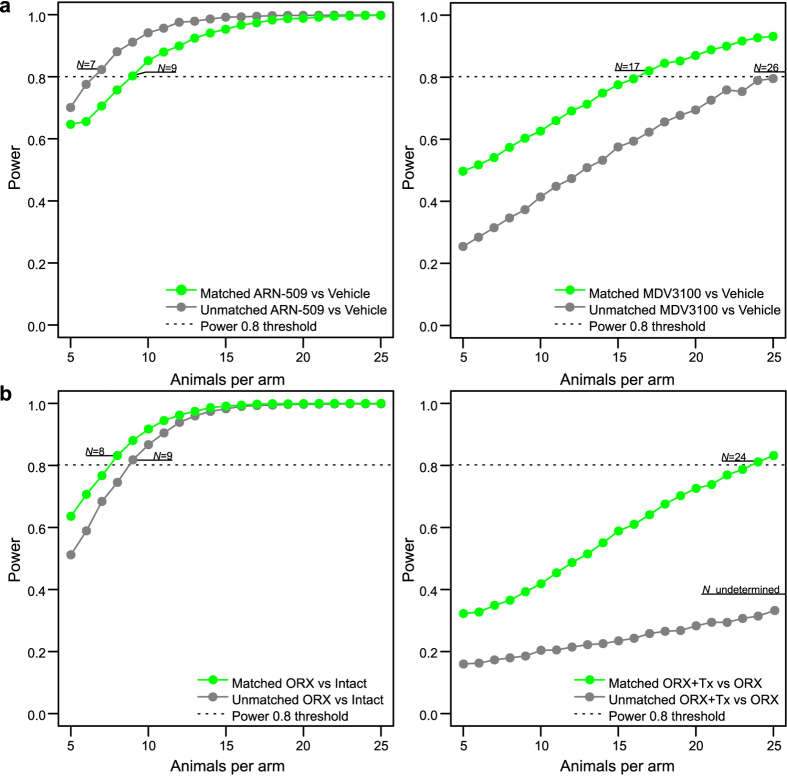
Model-based power calculations for sufficient sample size estimation. Statistical power (the likelihood that a true treatment effect is detected) as a function of the sample size (animals per treatment arm). Power calculations were computed by bootstrap re-sampling, either without the matching information (unmatched) or using the information from the optimal pairs of matched samples (matched). The estimated sample sizes (*N*) are defined based on the conventional threshold of 0.8 power. (**a**) ARN-509 and MDV3100 intervention effects in the VCaP mouse xenografts. (**b**) ORX and ORX+Tx intervention effects in the orchiectomized (ORX) VCaP mouse xenografts.

When applied to the two VCaP xenograft studies, the model-based calculation enables one to estimate the study power as a function of tumors per treatment group. Although the power calculation can be done with respect to each of the terms in the mixed-effects model, we focused here on the intervention-specific term *β*_intervention_ (Fig. 3c). With the more prominent intervention effects from ARN-509 and ORX, the power calculation led to similar sample size estimates between matched and unmatched models (*n* < 10; Fig. 4, left panel). However, there were notable differences in the number of animals needed when more complex or subtle interventions effects were studied; with MDV3100, the matched analysis reached the conventional power level of 0.8 at much smaller sample size compared to the unmatched model (*n* = 17 vs. *n* = 26; Fig. 4a, right panel), whereas for the intervention effect from ORX+Tx combination, the unmatched analysis remained below the sufficient power level with any practically feasible number of animals (Fig. 4b, right panel).

Although the power simulations were performed here retrospectively, these results already demonstrate that statistical inference of the intervention effects is highly dependent on the expected effect size and within-group variation, suggesting that future experimental designs should be tailored for each case individually, using e.g. data from a pilot experiment, so that the power calculations will meet the expected response patterns. Given the relatively large difference in the number of animals needed to reach sufficient power using an unmatched or matched approach, especially with the less evident cases (MDV3100 and ORX+Tx interventions), it is recommended that post-intervention statistical procedures should be defined already before the initiation of the study using tools such as proposed here^25^. However, a more fair comparison point for the matched approach would require a new study, conducted without using the baseline matching information, but this was not carried out within our preclinical studies because of ethical reasons.

## Discussion

The importance of controlling for individual variation is well-acknowledged in human clinical studies, with the aim to increase the study validity and reproducibility^26^. Similarly, in preclinical studies, reproducibility of the findings is associated with transparent reporting and paying careful attention to the experimental issues, including balancing, randomization and blinding^2^,^3^. Even though preclinical experimental designs differ from the truly randomized treatment group testing applied in clinical trials, the preclinical studies should benefit from the best practices of human clinical trials to improve their translatability^11^,^15^. We demonstrated here that a more detailed animal matching and statistical modeling offers many benefits across the different phases of the preclinical intervention experiment (Fig. 1). Prior to the interventions, the baseline balancing makes the experimental and control groups as similar as possible, while the matching-based randomization ensures that all the animal groups are sufficiently representative of the underlying population. This should reduce confounding variability and false positives in the subsequent testing of the intervention effects. During interventions, blinding promotes comparable handling and treatment of the animals by experimenters, while the estimated model parameters can detect outliers and provide insights into dynamic changes in individual animals in response to the interventions, such as non-linear treatment effects in the intervention groups. This makes the outcome measurements more uniform and reduces bias when reporting the results. After the intervention period, the paired longitudinal analysis of the individuals or tumours that were similar at baseline can be utilized in more sensitive detection of treatment effects (analogous to the paired *t*-testing). This may reduce false negative detections, especially when testing more subtle or complex treatment relationships, such as the MDV3100 and ORX+Tx treatment responses considered in the present study. While demonstrated here in the context of orthotopic xenograft studies, the statistical analysis and design issues are widely applicable also to genetically-modified mouse models (GEMMs), and should be even more important with the use of patient-derived xenografts (PDX), where the tumor material is limited and unique to each patient case^20^.

### Power calculations in preclinical animal studies

Power calculations are routinely demanded in human clinical studies, and recent reports have called for more rigorous sample size estimation also in preclinical animal studies^9^,^20^. Our model-based simulations enable the full use of response data from a pilot study or similar studies in the literature when estimating the sufficient sample size, rather than guessing or predicting the key model parameters and their variance. Furthermore, sampling of observations from a pre-fitted mixed-effects model offers a possibility to also incorporate indirect intervention effects, such as censoring due to death or animal exclusion, which may be difficult or even impossible to infer otherwise when determining the model parameters. Finally, the mixed-effects model requires the experimenter to specify the tested population hypotheses and the particular model structure already in the study design phase, which effectively discourages exploratory cherry picking and fishing for the ‘optimal’ results, a practice which severely reduces the reproducibility of the findings^27^. We note that the power simulations carried out in the present study were performed retrospectively, and hence, are applicable to designing future studies only^28^. When testing for more subtle or complex treatment effects, such as the +Tx effects in the ORX mice, sufficiently large sample sizes were required to provide statistically robust results. Even if this may lead to unexpectedly high number of test animals, it is widely acknowledged that underpowered or otherwise poorly designed studies are not only unethical but also contribute to both delays and increased costs of drug development process^4^,^9^.

### Exploratory and confirmatory study design issues

Table 2 summarizes the experimental design issues that we feel are essential to consider while performing statistically robust preclinical intervention studies. These issues are important both for exploratory and confirmatory preclinical studies, in order to improve their generalizability and translatability toward human diseases^29^. Exploratory studies involve preclinical screening and pathophysiological hypothesis testing, placing therefore more focus on detection sensitivity, whereas confirmatory studies are geared more toward efficacy estimation and clinical translation, where specificity of the findings is often more important. These two study classes serve as examples of the various inferential aims of the preclinical studies, and we hope our considerations will complement the current ARRIVE guidelines^15^, in terms of the statistical design and analysis of intervention effects. However, there remain several other factors that are outside the scope of the statistical methods introduced here, which may have much bigger role in the generalizability and translatability of the preclinical findings. For instance, although the internal variation in the treatment response can be controlled to a large extent using the matching and randomization methodology, these cannot normalize the effects of external factors, such as the representativeness of the animal model of the actual human disease, its target population and heterogeneity^3^,^30^. Additionally, although the animal matching can be performed based on multiple prognostic preclinical variables, these are unlikely to directly translate into the clinical use due to differences in the preclinical and clinical experimentation and physiology. However, the success rate of the human clinical trials is likely to benefit from a more accurate modelling of the heterogeneous treatment responses already during the preclinical phase^3^.

### Additional simulations of the model performance

An important practical question is how many and what type of baseline covariates should be used for animal matching. To address this question, we performed extensive simulation study (see Supplementary Methods), which confirmed and extended the results from our real case studies, showing that the matching information and paired analysis improves the statistical inference beyond the conventional approaches; this improvement was systematically observed across the number and type of covariates in terms of both detection sensitive and specificity (Supplementary Fig. S9). The largest benefits of the matching was gained with a selected panel of predictive baseline markers (e.g. 3–10 most informative covariates), in relatively small-sized studies (*N* = 5 to 10), but even if performed using non-informative markers and in larger studies, matching did not lead to reduced sensitivity or specificity. We therefore recommend preclinical researches to use several expert-curated baseline variables to improve the animal allocation and statistical testing, with a focus on the most relevant markers for the particular inference task (Table 2).

We further performed simulation studies to assess the relative advantages of the matched regression modelling in comparison to a more standard adjusted regression modelling, where the baseline confounders are incorporated as covariates in the post-intervention testing phase (Supplementary Material). Such post-intervention adjustments in the regression modeling may suffer from confounders interacting with the intervention effect, which may be difficult to track down and control for retrospectively in the intervention effect testing, as well as from an uncertainty about which confounders should be incorporated as the regression coefficients. We noticed that a matched-based animal allocation systematically improved over the adjusted regression, while the use of the pairwise matching information in the regression modeling led to the overall best sensitivity and specificity (Supplementary Fig. S10). Taken together, these simulation results show that the matching-based design and statistical analysis generally outperforms the more conventional approaches that do not use the baseline matching information.

### Current limitations and future perspectives

The presented methodology has certain limitations and potential caveats that should be understood. First of all, our specific focus here was on preclinical *in vivo* animal models, while the other forms of preclinical research are beyond the scope of this work. However, similar methods could be used also for *in vitro* experiments, where genetic and chemical perturbations and interventions are extensively modeled using dissimilarity-based methods analogous to the matching-procedure presented here^31^,^32^. Further, our methodology is implemented in the context of conventional preclinical study period, where animals are first selected for study inclusion, then baseline variables are measured based on which all the animals are randomly allocated into intervention groups (Fig. 1). Although the mixed-effect statistical model effectively captures the dynamic changes in the intervention responses, the baseline-based dissimilarity metrics do not typically consider time-dependent covariates; however, one can carry out also a longitudinal randomization procedure using, for instance, dynamic allocation methods that take into account dynamic cohort additions, covariate structures and intervention responses^33^. Finally, although both of our example cases were longitudinal intervention analyses of the tumor growth as a function of time modeled using linear mixed-effects models, the experimental design approach is also applicable to single end-point comparisons as a special case. We demonstrated this through the use of multivariate extension of the standard *t*-test (so-called Hotelling’s *T*^2^ test) in the VCaP xenografts, where we observed that the PSA surrogate marker correlated well with the primary outcome of tumor volume. Importantly, it was shown that the detection sensitivity of the subtle treatment effect of MDV3100 was increased when the end-point markers were coupled with the matching information through paired *T*^2^-testing (Supplementary Fig. S11).

As a future work, it will be important to perform a more systematic review and evaluation of the practices and factors that affect treatment assessment in preclinical intervention studies *in vivo*. These include experimental factors, such as measurement frequency, structured missing information due to both lower censoring at response detection limit and right-censoring at death, extent of pseudo-replication and confounding variability due to correlated structures (e.g., multiple tumors), as well as dynamic changes in the treatment effects over time. In particular, non-random missing values pose challenges to any statistical testing approaches, including our matching-based post-intervention testing procedure, which assumes that both of the paired individuals have been observed in order to effectively model the pairwise treatment differences. Such procedure creates the caveat that highly aggressive tumor groups, which are often being censored due to ethical reasons, may fail to provide representative animal/tumor pairs with those individuals with fully-observed longitudinal response profiles. This aspect of the pairwise matching procedure may actually provide also an advantage compared to the standard statistical methods, which often treat all the missing data as missing-at-random (MAR), as censoring removes pairwise differences from both of the animals that have a matched baseline profile; therefore, right-censored missingness will not accumulate only to aggressively growing groups. Although it is possible that this allows for less-biased estimates, provided that the prognostic matching covariates can accurately predict the response, this potential advantage may come at the expense of decreased power to detect the longitudinal intervention differences as dominant right-censoring may result in insufficiently short pairwise longitudinal trajectories. Due to the complex nature of non-random missingness in the post-intervention testing, systematic evaluation of these effects warrants a separate future work in various preclinical models and experimental setups.

## Methods

### Optimal non-bipartite matching problem formulation

Matching was used to allocate individual animals into homogeneous subgroups according to a pre-defined dissimilarity criterion^34^ (Fig. 2; Supplementary Fig. S7a,b). Multiple baseline variables that may have either prognostic or confounding contribution to the treatment response were simultaneously used for balancing the treatment and control groups through the pre-selected dissimilarity metric (Supplementary Table 1). By incorporating such baseline information, the experimental design allows for more sensitive and specific detection of effects that are due to the interventions alone^35^. In theory, matching should not introduce loss of statistical power even when performed on irrelevant covariates^34^. Since purely deterministic allocation methods have been criticized for the risk of introducing experimental biases due to, for instance, the lack of masking^36^, our constrained randomization procedure incorporated also a stochastic component, making it fully compatible with the current clinical recommendations of random allocation and balancing at baseline. The matching-based randomization approach refines all possible allocations from a single pool of individuals, and then randomly picks one of these most feasible allocation solutions. As such, the procedure greatly resembles the randomized block design, which is used in the clinical field to adjust for pre-intervention randomizations by stratifying for categorical factors (e.g. gender) or bins of numeric values (e.g. adolescent/adult/elderly), especially in studies with small or moderate sample size^37^.

Expanding the previous formulation^35^ (Supplementary Fig. S7b), the optimal non-bipartite matching problem can be formulated as follows. Let us consider binary symmetrical matching matrices ***X*** of size *N* × *N* where:


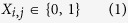



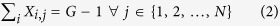



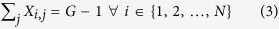



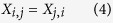



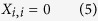


The two sum constraints in equations (2 and 3) guarantee that the number of edges originating from a single observation equals the number of desired groups minus 1. Here, *G* denotes the number of desired members per each matching structure, and is equal to the number of desired intervention groups. This means that all the rows sum to *G*-1 and columns to *G*-1 in the binary matching matrix ***X*** (Fig. 2b). Dissimilarity matrix ***D*** of size *N* × *N* is defined as:


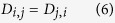



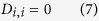


Each element *D*_*i,j*_ is computed according to the chosen dissimilarity metric with possible alternatives summarized in Supplementary Table S1. The interpretation of the constraints are follows; in equation (1): For each possible pairs of individuals *i* and *j*, the pair is either matched (value 1, connected with an edge) or not matched (value 0); (2): Each individual *j* is connected to other *G*-1 matched individuals; (3): Each individual *i* has *G*-1 other individuals that are matched to the individual *i*; (4): If individual *i* is matched to individual *j*, then individual *j* is also matched to individual *i* (no single direction relationships allowed); (5): An individual may not be matched to itself; (6): The similarity of individual *i* to individual *j* is as great as similarity of individual *j* to individual *i* (no directionality allowed). (7): An individual is always perfectly similar to itself.

The existing optimal non-bipartite matching algorithms, for example, the one presented in the R-package ‘*nbpMatching*’^35^, consider paired non-bipartite matching, where:


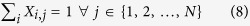



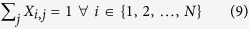


We expanded upon this problem and developed a global optimization algorithm for solving this generalized problem. In order to introduce multigroup matches, we define fully connected structures called *submatches*. Matches are considered as graphs {*V, E*}, where *V* is vertex (node) and *E* is an edge between vertices. If observations *i* and *j* have not been matched, their edge is non-existing (*X*_*i,j*_ = 0). Each submatch *M*_*k*_ is a subgraph of *V*, where the number of vertices belonging to the *k*:th submatch *M*_*k*_ equals to *G*, that is, the number of desired groups. The matching matrix has to have edges between all of the elements belonging to *M*_*k*_, that is:


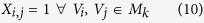


Furthermore, the submatches are non-overlapping, in the sense that no edges are allowed to exist between these substructures:


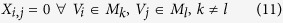


The total number of these substructures is *N*/*G* in the matching solution. Supplementary Fig. S3 shows the matching problem in the ARN-509/MDV3100-experiment with the desired number of groups *G* = *5*, which illustrates the increase in computational complexity as the number of edges within a submatch increases per binomial coefficients. The optimal matching problem is:


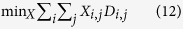


The optimization problem in equation (12) is used to identify the matching matrix ***X*** that minimizes the sums of distances that fulfill the constraints in equations (1, 2, 3, 4, 5) for a given distance matrix ***D*** with desired submatch size *G*. These identified submatches may then be used to allocate the intervention groups (Fig. 2), with possible additional constraints as described in Supplementary Methods.

### Mixed type baseline information in the matching

We used categorical variables alongside numerical variables in the matching problem. We divided this into two options: (i) *relaxed*, where the categorical information increments distance at *D*_*i,j*_ by a certain amount if the two observations *i* and *j* originated from different categorical labels, and (ii) *strict*, where observations with separate categorical labels may never be matched by setting their relative distance to infinity (*D*_*i,j*_ = ∞). Observations of *relaxed* type may be part of the same submatch even if they have different labels, provided that their similarity in the numerical dimensions dominates over the categorical difference. Whether or not this happens, depends on the chosen distance metric (Supplementary Table S1); for example, the Gower’s dissimilarity^38^ is a popular choice for combining mixed type information, but also other metrics have been proposed^39^,^40^,^41^. In the *strict* approach, two observations with different categorical labels may never be part of the same submatch, and therefore this option eliminates a large fraction of possible solutions by limiting the search to a smaller solution space due to infinite values in ***D***. This approach also forces each intervention group to contain an equal number of members from each sub-strata.

### Branch and bound algorithm (exact optimization)

The number of possible ***X*** binary matching matrix solutions that fulfill the constraints set in equations (1, 2, 3, 4, 5) increases exponentially as a function of the number of individuals participating in the matching. For detection of the global optimum of equation (12) in the discrete optimization task, a branch and bound algorithm relies on implicit exhaustive enumeration of all possible combinations in a tree-like structure. Within this structure, however, massive amounts of solutions are omitted based on knowledge that the omitted solutions could theoretically not be better than the current best found solution. If a branch of solutions may include a solution better than the current best found solution, it has to be searched through enumeration. The algorithm itself may be depicted as traversing a tree-like structure using alternating steps called the branching step that expands the current search tree, and the bounding steps that omit large non-optimal areas of the search tree (Supplementary Fig. S7c,d). These *branch* and *bound* steps are described in detail in our Supplementary Material, along with an alternative heuristic Genetic Algorithm (GA) that provides a faster non-exact optimization alternative for large studies.

### Matched mixed-effects modeling of treatment effects

In order to evaluate the effect of interventions in a longitudinal study, we assumed that the response variable *y* (e.g. PSA concentration) for the *i*:th tumor from the intervention group *g*_*1*_ or *g*_*2*_ grows according to the following linear model:


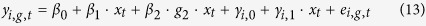


Here, variable *x*_*t*_ ∈ ℕ_0_ indicates the *t*:th time point in the study (e.g. day or week since starting the interventions). Fixed effects *β*_0_ and *β*_1_ correspond to be population based effects, where *b*_0_ models the initial average response value at baseline (*x*_*t*_ = 0), and *b*_1_ models the longitudinal expected linear growth pattern of the tumor, while *β*_2_ includes a binary indicator *g*_2_ which obtains value 1 if the *i*:th tumor belongs to the group *g*_*2*_and 0 otherwise. Random effects γ_*i,0*_ and γ_*i*,1_ model variation of the *i*:th individual in the initial response levels or in the growth rate patterns, respectively, and these are analogous to the fixed effects *β*_0_ and *β*_1_. We modeled random noise with the error term *e*_*i,t*_, and the error and random effects are assumed to be normally distributed:


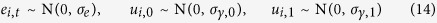


The unmatched model in equation (13) does not incorporate supporting prognostic matching information beyond the baseline levels of the main response *y*, although tailored modeling approaches exist for similarly formulated models^42^,^43^. Therefore, we propose a matched mixed-effects model, which incorporates the matching information obtained from the matching of pairs {*i,j*} before the interventions:


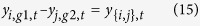


where the submatched individuals *i* and *j* have been allocated to different intervention arms *g*_*1*_ and *g*_*2*_ as described in (Figs 1 and 2). The resulting time point specific pairwise observations are then modeled longitudinally using a mixed-effects model:


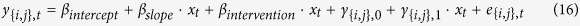


where by default we propose setting *β*_*intercept*_ = 0 and *β*_*slope*_ = 0 due to their redundancy in the matched curves (see Fig. 3c bottom panel for the visual interpretation). While γ_0_ effectively models the baseline (*x*_*t*_ = 0) individual level random intercept for the response variable *y*, the model term γ_1_ allows pairwise variation in the growth slopes. This allows prognostic inference for the population effects, especially for the inter-group growth difference in the fixed effect *β*_*intervention*_, since additional baseline experimental factors are incorporated through the matching {*i,j*}.

The mixed-effects model fitting was performed using the *lme4*-package^44^ in the R statistical software^45^. In Table 1, the *p*-values for fixed effects *β* were computed using Satterthwaite’s approximation for degrees of freedom using the *lmerTest*-package^46^, while significances of random effects *u* can be tested using log-likelihood ratio tests as proposed in literature^47^. The concept of matching-based mixed-effects modeling is presented in Fig. 3. Example *Unmatched* equation (13) and *Matched* equation (16) model fits are shown for the Control vs. MDV3100 comparison (Single submatch visualized in Fig. 3a of the total 15 pairs). Due to incorporating prognostic submatch-information to the modeled curves (Fig. 3a,b), the matched inference resulted in an increase in sensitivity (Table 1, Fig. 4). Complete visualizations of the model fits are given in the Supplementary Figs S5 and S6. Interestingly, prognostic accuracy in the intervention testing was most likely allowed by the pairing of similar curves in ORX+Tx vs ORX testing (Supplementary Fig. S6b), where the matched curves retained approximately linear trends despite the lack of an early PSA nadir.

### Power simulations from experimental datasets

Power analysis is important to ensure statistical validity of the experimental findings. So far, reliable resources have not been available for the preclinical studies in which the experiments pose a number of specific requirements, namely the complex nature of longitudinal responses, right-censoring occurs due to death of animals, limited number of individuals, batch-wise effects, and multivariate baseline characteristics. We addressed these challenges by offering a sampling based power analysis tool that samples individuals with replacement (bootstrapping) from a pre-fitted mixed-effects model, and then re-fits the specified statistical model to the sampled datasets. The method then provides a power curve as a function of *N* in respect to each of the tested population hypotheses. We draw inspiration for this simulation approach from literature^48^, although we propose sampling by bootstrapping the data, rather than based on the mixed-effects model parameters.

There are a number of advantages in evaluating the power of a study through simulations: (**i**) Data based simulations do not force the experimenter to perform an expert guess on an often non-intuitive model parameter and its variance to assess required sample amounts. Instead, the experimenter may provide artificial data, e.g. data observed in literature or in pilot studies. This approach is drastically more concrete and expert curated approach to the task. (**ii**) By sampling observations from a pre-fitted mixed-effects model, our approach offers possibility to incorporate also indirect effects, such as censoring due to unexpected death of animals during the study, which may be otherwise difficult or impossible to infer directly for the model parameters. (**iii**) The sampling function relies on a readily fitted mixed-effects model for data input, automatically identifies a suitable sampling unit, and then re-fits the statistical model to the sampled datasets. This feature requires the experimenter to readily specify tested population hypotheses and the structure of the mixed-effects model already in the design phase of the experiment. By requiring such pre-experiment coordination of the tested hypotheses and pre-specified structure of the model, our method encourages well specified á priori hypotheses.

### Ethics Statement

All mice were handled in accordance with the institutional animal care policies of the University of Turku (Turku, Finland). The animals were specific pathogen-free, fed with complete pelleted chow and tap water *ad libitum* in a room with controlled light (12 h light, 12 h darkness) and temperature (21 ± 1 °C). The two animal experiments were approved by the Finnish Animal Ethics Committee (licenses ESAVI/1993/04.10.03/2011 and ESAVI/7472/04.10.03/2012). The institutional policies on animal experimentation fully meet the international requirements as defined in the NIH Guide on animal experimentation. Supplementary Methods provide further details of the intervention experiments and Supplementary Note *the ARRIVE guideline checklist* for the two animal studies.

## Additional Information

**How to cite this article**: Laajala, T. D. *et al*. Optimized design and analysis of preclinical intervention studies *in vivo*. *Sci. Rep*. **6**, 30723; doi: 10.1038/srep30723 (2016).

## Supplementary Material

Supplementary Information

## Figures and Tables

**Table 1:** Mixed-effects model fits for the fixed effects (population inference) and random effects (individual effects and the random error term).

		Fixed effects (*p*-value)			Random effects (SD)		
		Model		β_*intercept*_	β_*slope*_	β_*intervention*_	γ_*intercept*_	γ_*slope*_	ε_*error*_
ARN-509 vs Control	*Unmatched*	14.311 (<0.001)***	10.062 (<0.001)***		8.234	5.163	5.749
	*Matched*	0 (−)	0 (−)	−7.962 (0.0047)**	7.053	8.894	8.399
MDV3100 vs Control	*Unmatched*	13.536 (<0.001)***	10.188 (<0.001)***	−4.940 (0.0494)*	7.635	6.259	6.395
	*Matched*	0 (−)	0 (−)	−5.729 (0.0160)*	7.013	7.401	11.247
ORX vs Intact	*Unmatched*	14.548 (<0.001)***	1.336 (<0.001)***	−1.265 (0.0034)**	14.578	0.997	8.518
	*Matched*	0 (−)	0 (−)	−1.931 (0.0063)**	4.251	2.157	9.522
ORX+Tx vs ORX	*Unmatched*	9.998 (<0.001)***	0.122 (0.0675)N.S.	**−0.101 (0.2704)N.S.**	10.476	0.167	9.977
	*Matched*	0 (−)	0 (−)	−0.112 (0.0457)*	2.381	0.155	4.618

Model estimates and their significance levels using the conventional unmatched and matching-based pairwise models are presented for each intervention comparison separately.

The model term that explicitly tests for an intervention effect is highlighted in bold. N.S., not significant; *p < 0.05; **p < 0.01; ***p < 0.001.

**Table 2:** Experimental design issues in exploratory and confirmatory preclinical studies.

Design issue	Exploratory study	Confirmatory study	Aims and benefits
Study objective (focus on sensitivity/precision or specificity/generalizability)	Preclinical screening and pathophysiological hypothesis testing (*sensitivity*)	Estimating effect size and ensuring clinical translation (*specificity*)	Sensitivity allows effective search for intervention candidates, while specificity emphasizes translational aspects. Notably, mere statistical significance in preclinical testing does not yet guarantee clinical relevance
Example animal models^19^	Traditional cost-efficient models, e.g. subcutaneous xenografts	Translational models, e.g. orthotopic xenografts, PDX, GEMM	Seeking a balance between cost-efficiency and translatability
Number of intervention groups (Parameter *G*)	High number of candidate intervention groups (Prefer *G* over *N*)	Carefully selected interventions to be validated (Prefer *N* over *G*)	High *G* allows effective exploration of novel candidates for downstream confirmatory studies
Number of animals in each intervention arm (Parameter *N*)	Focus on testing multiple candidate intervention groups at sufficient sample size (medium *N*)	High confidence required for true positive effects as well as for effect size estimate (high *N*)	Well-characterized animals and sufficient *N* allows better translation to the target population and improved generalizability
Number of covariates *d* in matching (Data dimension *d*)	Many possible confounding covariates, with suspected effect on the primary response (flexible *d*)	Ideally only few selected confounding covariates, which affect the representative intervention outcome (low *d*)	Matched animals in separate treatment arms allows more accurate inference both in terms of sensitivity and specificity
Estimation of sample size for the study and effect sizes for the interventions	Often difficult due to lack of pilot studies for the candidate interventions	Key ingredient in ensuring sufficient statistical power^1^,^9^	Sufficient statistical power to identify true intervention effects and reject false effects. Accurate effect size estimation assists in evaluating clinical significance
Maximization of the consistency in handling of the individual animals and/or tumours	Relevant in all study aims	Relevant in all study aims	Prevent undesired stratification and false detections due to potential batch-effects
Taking into account potential dependence structures (e.g. tumours within the same animal)	Highly dependent on the number of *G* in relation to *N*. Some degree of compromise is acceptable to maximize sensitivity	Highly relevant, e.g. cage-effects are attributed to high attrition rates of preclinical findings 6,12,13,14	Prevents over-estimation of the required sample size due to so-called pseudo-replication^15^

Exploratory and confirmatory study aims adopted from Kimmelman *et al*.^29^
