# Supplementary material for: Optimized design and analysis of preclinical intervention studies in vivo
Source: Sci Rep. 2016 Aug 2;6:30723. doi: 10.1038/srep30723 (PMC4969752; doi:10.1038/srep30723)
Supplement: Supplementary Information [file srep30723-s1.pdf]

Supplementary Material for

Optimized design and analysis of preclinical intervention studies *in vivo*

Teemu D Laajala, Mikael Jumppanen, Riikka Huhtaniemi, Vidal Fey, Amanpreet Kaur, Matias Knuuttila, Eija Aho, Riikka Oksala, Jukka Westermarck, Sari Mäkelä, Matti Poutanen, Tero Aittokallio.

Supplementary Figures
Pages 2-12: Supplementary Figures S1-S11.

Supplementary Table S1
Page 13: Common dissimilarity measures and their key properties.

Supplementary Methods
Pages 14-20: Additional details for the preclinical experiments and algorithms.

Supplementary Note S1
Pages 21-46: Hamlet R-package: step-by-step user instructions.

Supplementary Note S2
Pages 47-61: R-vivo user instructions.

Supplementary Note S3
Pages 62-64: The ARRIVE checklist for the two animal treatment studies.

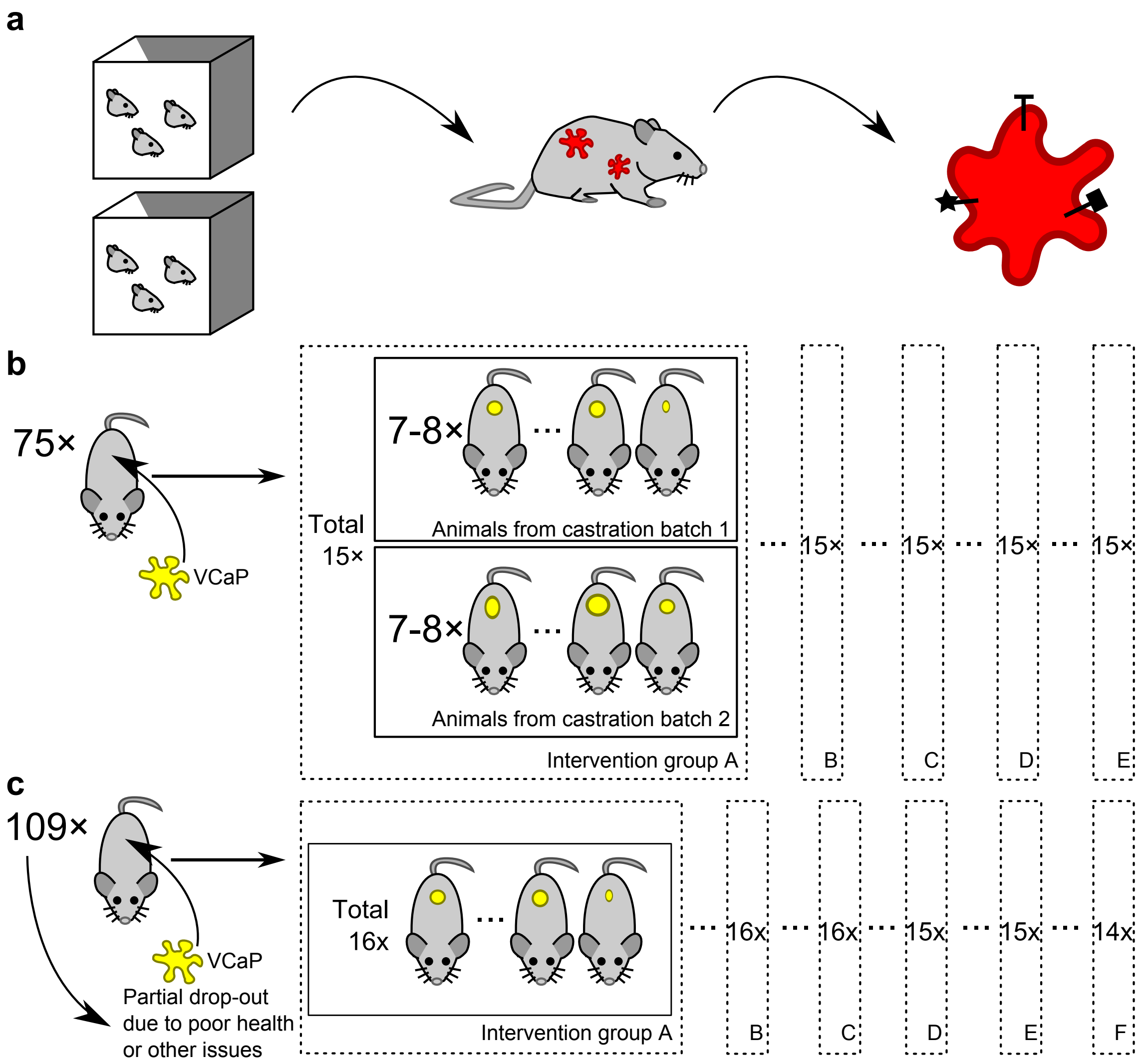

Supplementary Figure S1: Schematic illustrations of the common hierarchical structures that need to be taken into account in animal allocation and the experiment designs of the two case studies. (**a**) Preclinical cancer studies commonly incorporate a layered hierarchical design, where multiple nested animals may originate from a single batch or a cage, while multiple tumors may be located in a single animal. (**b**) ARN-509/MDV3100 -intervention study with orthotopic VCaP prostate cancer cells in male immunodeficient mice (HSD: Athymic Nude Foxn 1nu). According to the experimental procedure, orthotopic tumors were generated by injecting the cancer cells into the prostate of each animal. The growth of the tumors was followed by weekly measurements of the serum PSA indicating the tumor burden. The animals were castrated in two separate batches on subsequent weeks, resulting in two substrata with different tumor growth characteristics. The mice were followed by serum PSA measurements and after the re-appearance of the tumors the mice were allocated into several CRPC treatment arms . Hierarchical allocation procedure based on the global matching algorithm ensures that the substrata are evenly distributed among the intervention groups. (**c**) ORX/ORX+Tx -intervention study with analogous subcutaneous VCaP xenografts. A single substrata of animals was allocated into several intervention groups (out of which Control, ORX and ORX+Tx are presented in this paper), while some animals had to be dropped out due to ethical reasons.

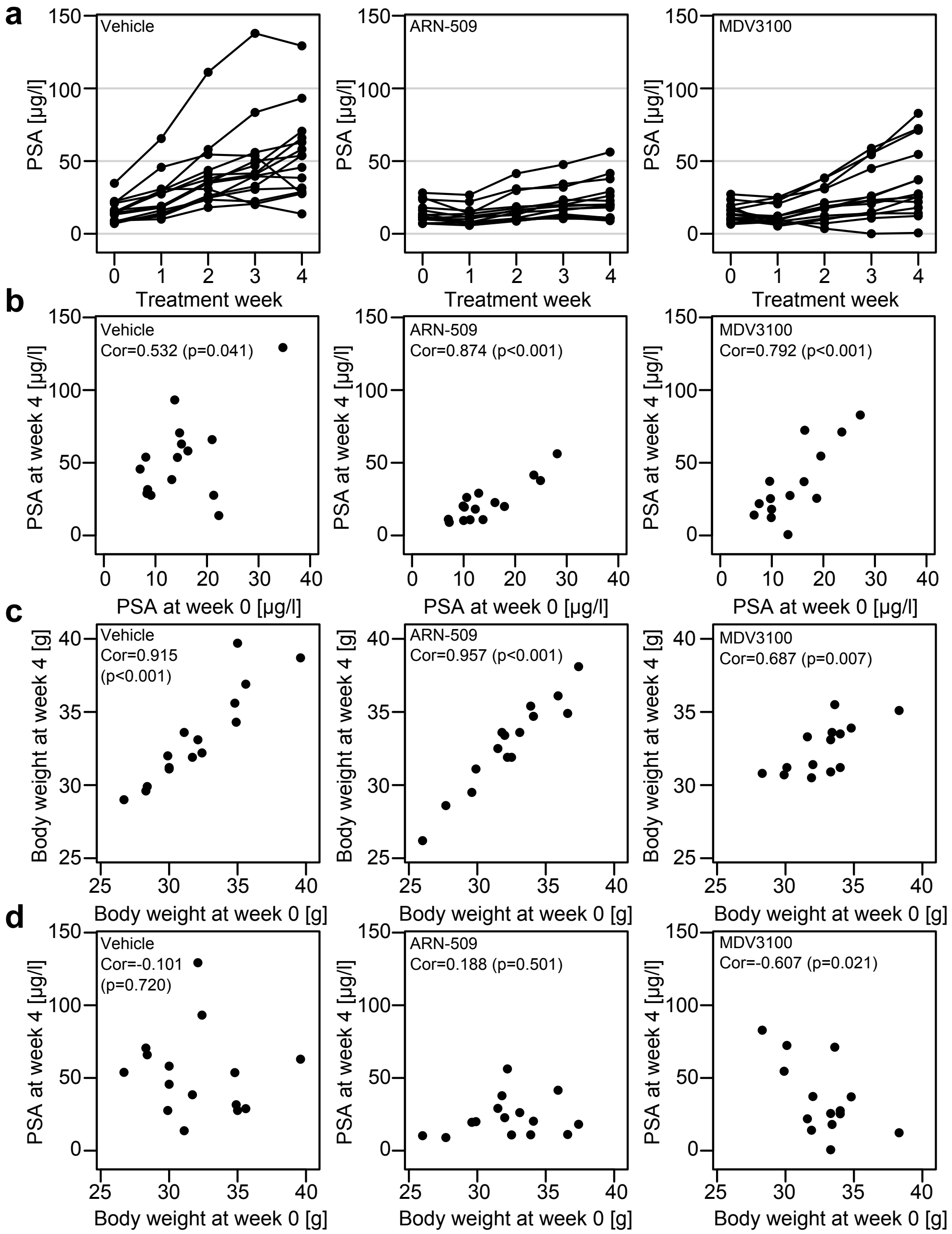

Supplementary Figure S2: Experimental data of the VCaP study. (**a**) Two selected treatment alternatives, ARN-509 (n = 15) and MDV3100 (n = 15), were compared to the vehicle group (n = 15). (**b**) As expected, the initial PSA level at baseline was predictive of the PSA value measured after 4 weeks of treatment. (**c**) Similarly, body weights of the animals at baseline were correlated with the body weights after 4 weeks of treatment. (**d**) Interestingly, the initial body weight showed a borderline inverse correlation with the PSA level after 4 weeks of treatment in the MDV3100 group (p=0.021), while this relationship was not seen in the other groups. Such a multivariate association between the treatment response (final PSA) and an initial animal characteristic (body weight at baseline) would be missed with simple univariate animal matching procedures.

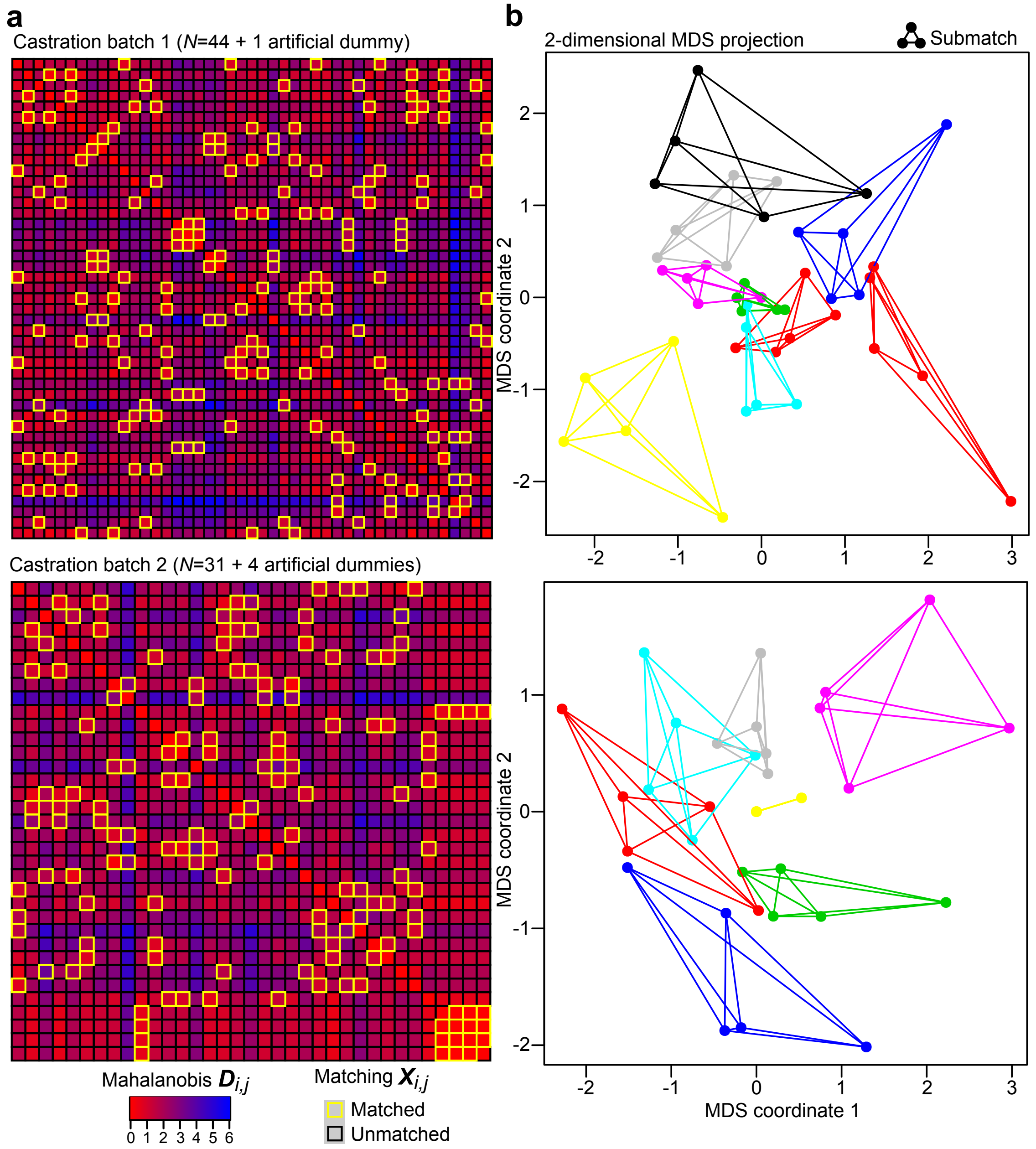

Supplementary Figure S3: Solving the non-bipartite submatching problem in the MDV3100/ARN-509 intervention study. (**a**) The animals (n = 75) were divided to two different castration sub-strata, which were separately submatched only within a strata and subsequently allocated evenly to the intervention arms (see **Supplementary Fig. S1b**). The matrix colors indicate dissimilarities in the baseline characteristics, and the box color indicates animals being part of same submatch. (**b**) Multidimensional Scaling (MDS) 2-dimensional projection of the complex baseline characteristics, with each submatch indicated with connecting edges and different coloring.

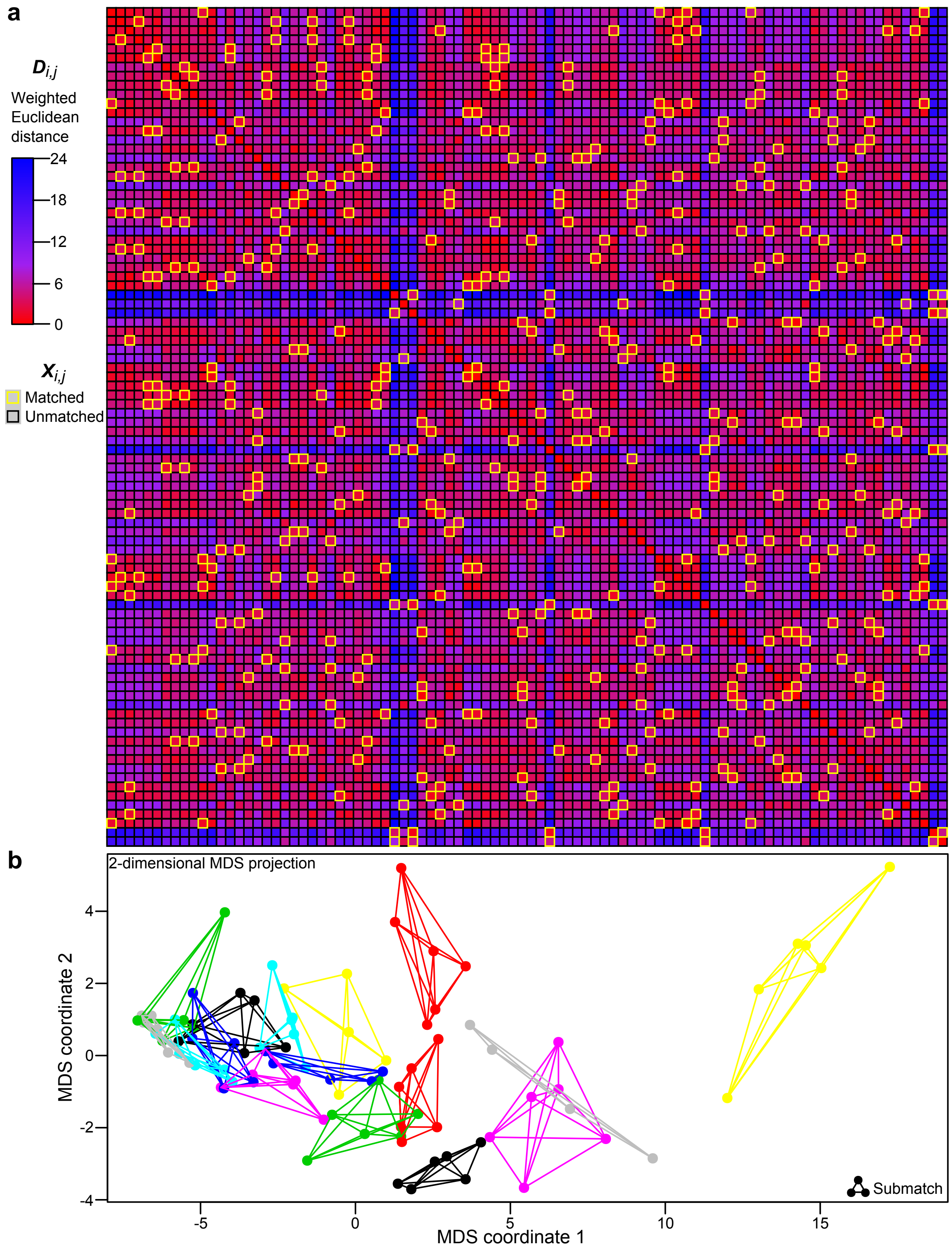

Supplementary Figure S4: Solving the non-bipartite submatching problem in the ORX/ORX+Tx intervention study. (**a**) The animals ($n = 109$) were matched to submatches of size 6, and subsequently allocated to different intervention arms within each submatch. Only three of the intervention groups are analyzed here (Control, ORX, ORX+Tx). The matrix colors indicate dissimilarities in the baseline characteristics, and the box color indicates two animals being part of same submatch. (**b**) Multidimensional Scaling (MDS) 2-dimensional projection of the complex baseline characteristics, with each submatch indicated with connecting edges and different coloring.

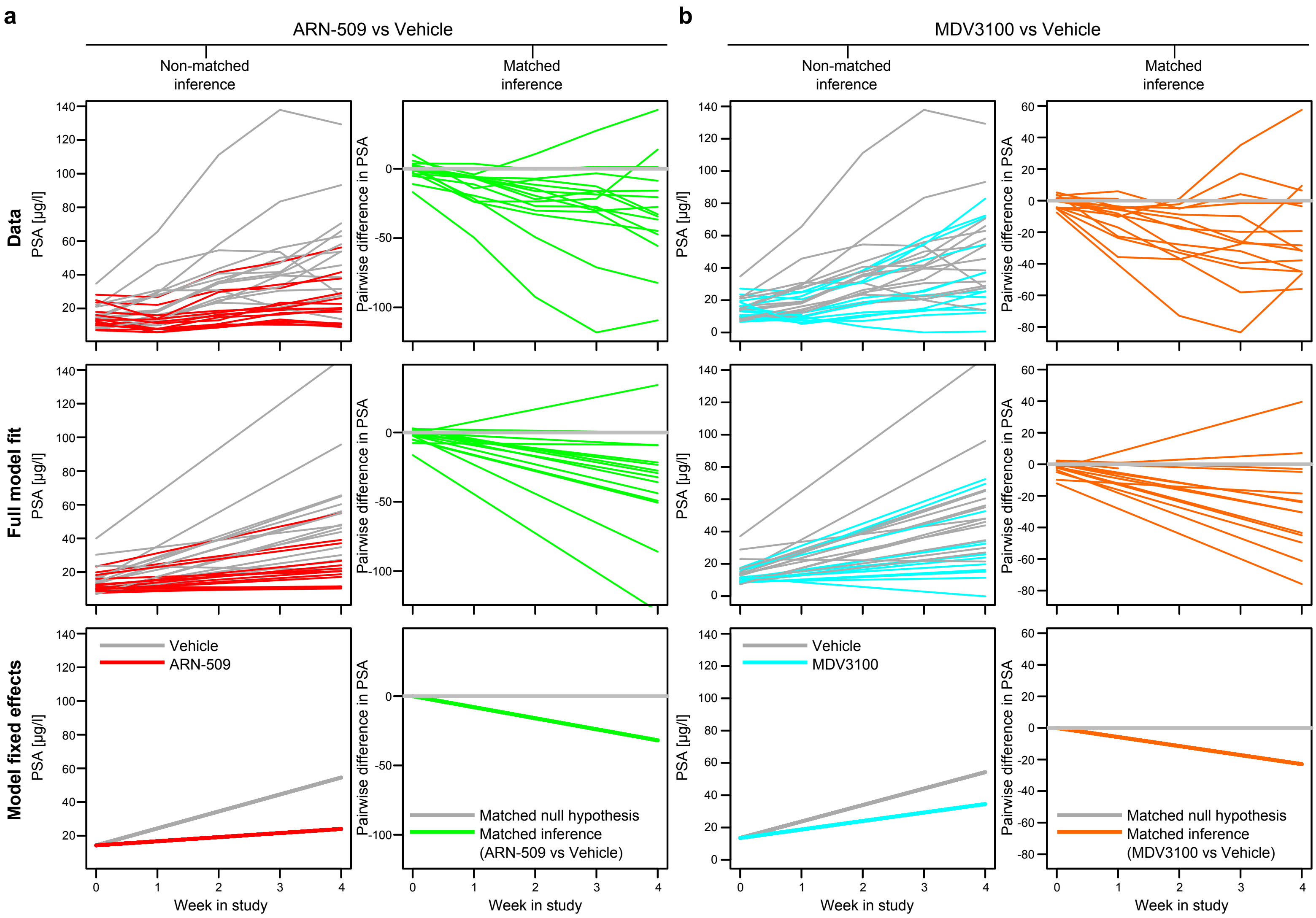

Supplementary Figure S5: Mixed-effects model fits in the ARN-509/MDV3100 intervention study. Top panel: response data; middle panel: full model fit; bottom panel: fixed effects fit. (**a**) ARN-509 versus Vehicle. Left panel: unmatched inference; right panel: matched inference. (**b**) MDV3100 versus Vehicle. Left panel: unmatched inference; right panel: matched inference. Model coefficient estimates, standard deviations and p-values are presented in **Table 1**.

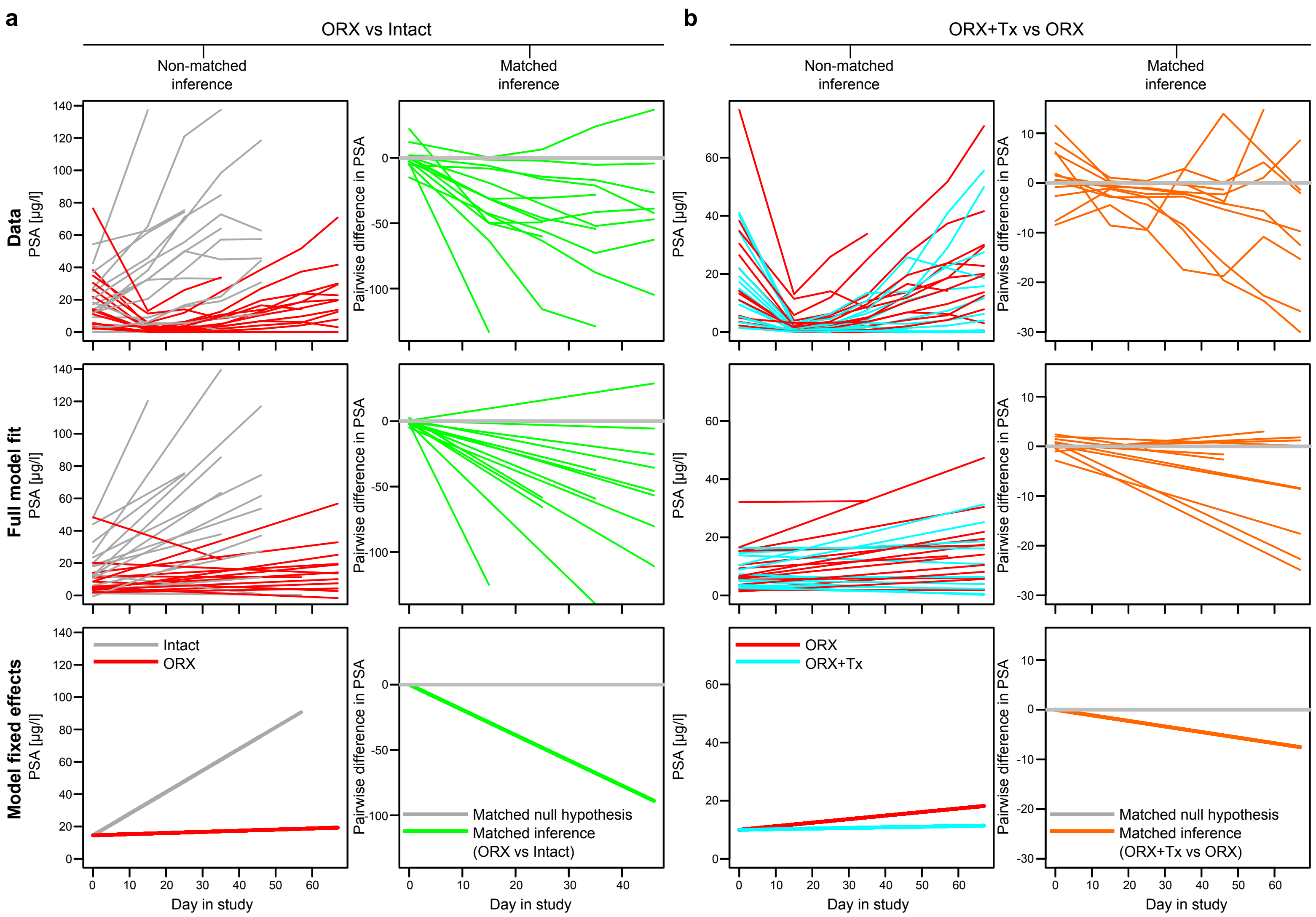

Supplementary Figure S6: Mixed-effects model fits in the ORX/ORX+Tx intervention study. Top panel: response data; middle panel: full model fit; bottom panel: fixed effects fit. (**a**) ORX versus Control. Left panel: unmatched inference; right panel: matched inference. (**b**) ORX+Tx versus ORX. Left panel: unmatched inference; right panel: matched inference. Model coefficient estimates, standard deviations and p-values are presented in **Table 1**.

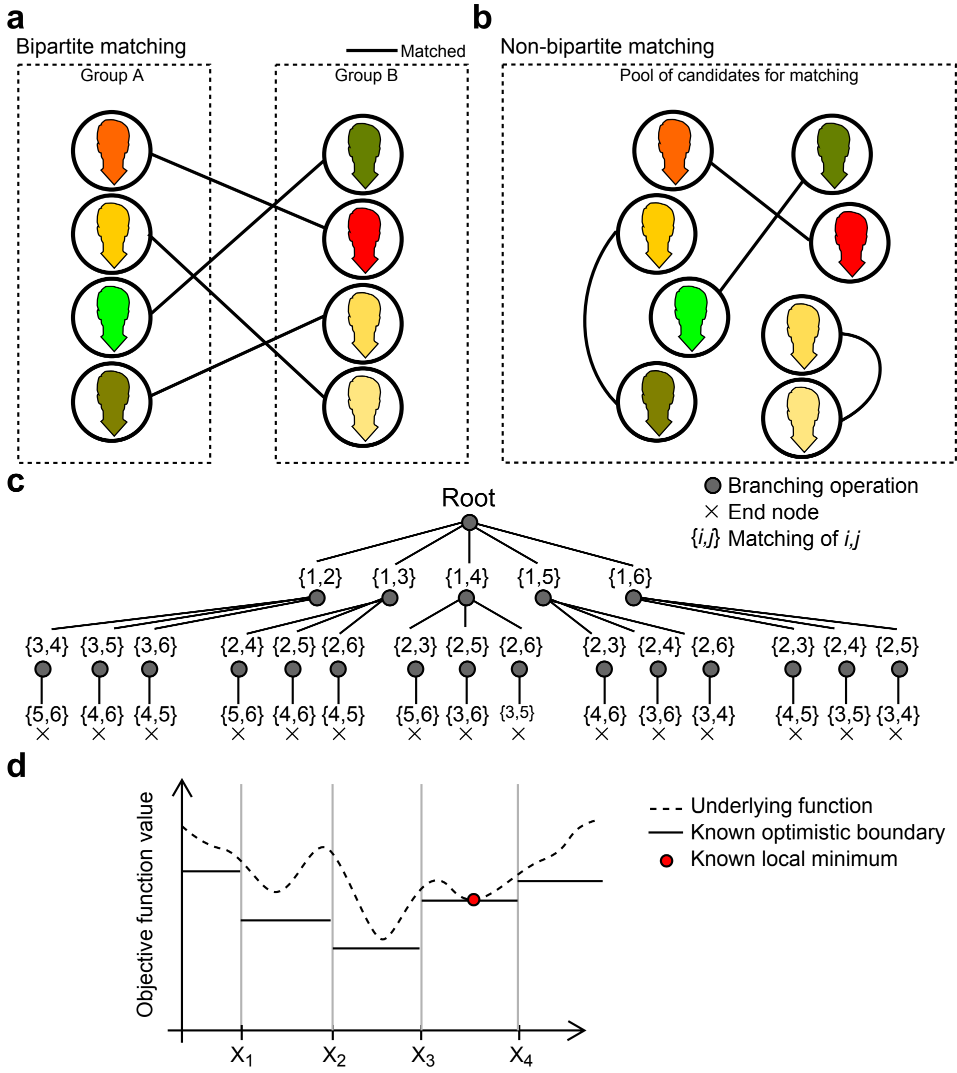

Supplementary Figure S7: The difference between bipartite and non-bipartite matching, and a graphical representation of the steps in the branch and bound algorithm for solving the non-bipartite problem. (**a**) A bipartite matching problem, where the matching is identified between two pre-defined groups. (**b**) In the preclinical cancer context, the non-bipartite matching enables detection of comparable individuals from a single pool of animals, based on similarities in their baseline characteristics. (**c**) Branching implicitly enumerates all possible combinations of matches in the solution. In this particular example, the branching structure is presented for matching of pairs ($G = 2$) for 6 individuals. (**d**) Concept of the bounding function in a continuous minimization task (lower objective function values are preferred). A bounding function is utilized to discard branches in the tree-like structure (panel **c**), by concluding that a certain range (branch) of solutions cannot improve the current best solution. In this example, ranges $x \leq X_1$ and $X_4 \leq x$ do not have to be searched, as the bounding function hints that the current best solution (indicated in red) cannot be improved in this solution range. However, solutions in $X_1 \leq x \leq X_2$ and $X_2 \leq x \leq X_3$ have to be tested, since the bounding function suggests a possible lower theoretical boundary in these solution ranges.

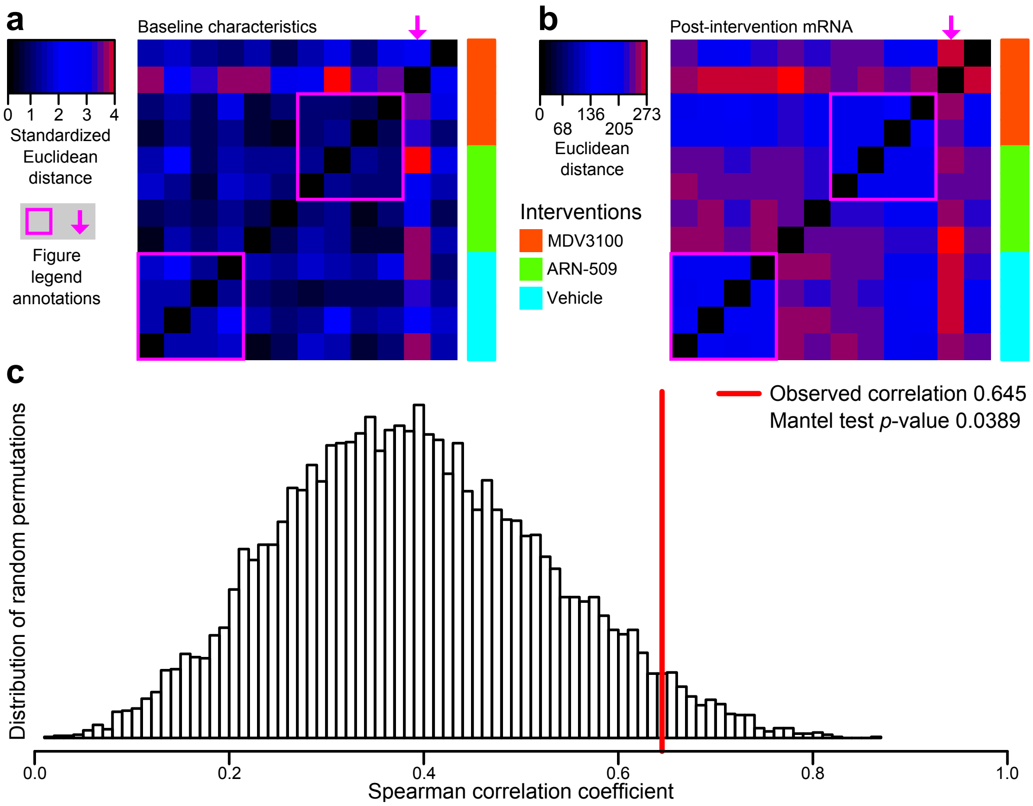

Supplementary Figure S8: Evaluation of the animal allocations in the ARN-509 / MDV3100 VCaP xenograft study using Mantel's test that compares the pre-intervention dissimilarity matrices of the baseline animal characteristics to the post-intervention mRNA gene expression profiles of the treated tumors. By visual inspection, two interesting dissimilarity sub-groups were identified (pink boxes). Further, one exceptional baseline animal remained an outlier also at the tumor mRNA-level (pink arrow). (**a**) Dissimilarity matrix of the baseline characteristics for the sequenced animals ($n = 12$) was calculated using standardized Euclidean distance. (**b**) Dissimilarity matrix of the RNA-seq expression profiles (fragments per kilobase of exon per million mapped reads, FPKM) was calculated using Euclidean distance. (**c**) Distribution of the permutated correlation statistic. Statistically significant Spearman correlation was observed between the baseline characteristics and post-intervention mRNA expression (red line), by conducting $n = 10,000$ permutations of the dissimilarity matrices (Mantel's test).

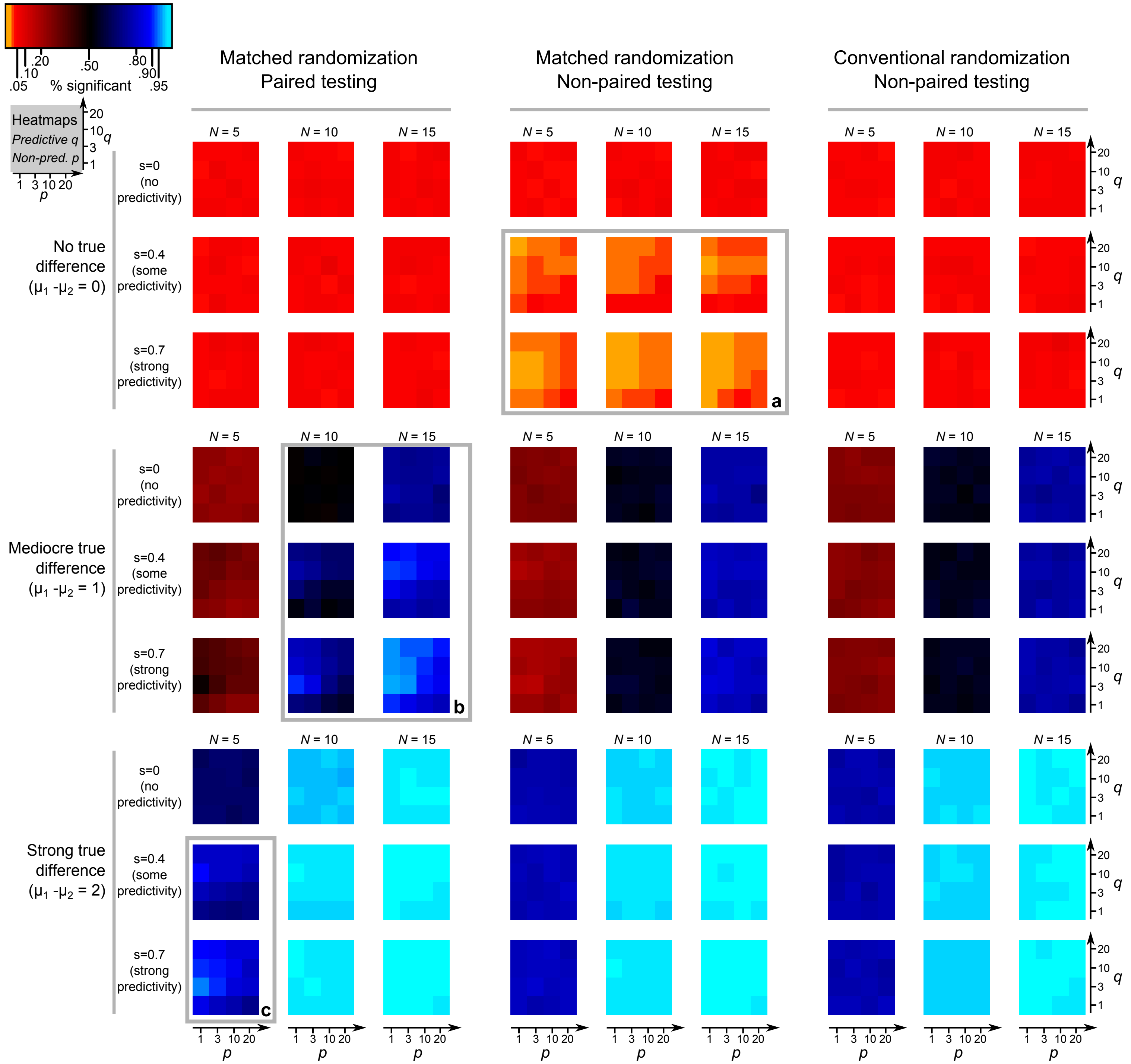

Supplementary Figure S9: A simulation run of 1,000 matched 2-group datasets were generated for each combination in the parameter grid, resulting in a total of 432,000 datasets for which matching was conducted and data drawn from mulvariate normal distributions with given parameters. The matching procedure was used as in the manuscript, and conventional randomization randomly allocated groups of equal size ignoring baseline information to both experiment groups. Paired or non-paired t-test was used to determine whether there was a difference with $\alpha = 0.05$ significance threshold. The following parameters were varied: Magnitude of true group difference $\mu_1 - \mu_2 \in \{0, 1, 2\}$; Sample size per group $N \in \{5, 10, 15\}$; Magnitude of informativeness in (parameter q) predictive baseline variables $s \in \{0, 0.4, 0.7\}$; Count of predictive baseline variables $q \in \{1, 3, 10, 20\}$; Count of non-predictive baseline variables $p \in \{1, 3, 10, 20\}$. Few interesting key results were annotated in the simulation results: (**a**) Interestingly, when matched allocation was used, the specificity in testing was highly increased in the case when no true group difference was present. This phenomenon persistent in the non-paired testing, highlighting that matching-based allocation also serves to improve specificity and that non-paired testing can be benefit even if the matching information is not utilized in the post-intervention testing. (**b**) A benefit in sensitivity was observed in the small group-wise different ($\mu_1 - \mu_2 = 1$) in comparison to the non-matched testing as long as the number of predictive markers was greater than non-informative baseline markers ($q \geq p$). As expected, this advantage was lost if no informative markers were present ($s = 0$), but no loss of accuracy was observed in comparison to the conventional methods. (**c**) In small explorative studies ($N = 5$), a slight advantage in sensitivity was observed especially if the baseline markers were highly predictive ($s = 0.7$), highlighting that predictive markers may help narrow down candidates more effectively in such explorative studies with typically smaller sample sizes N.

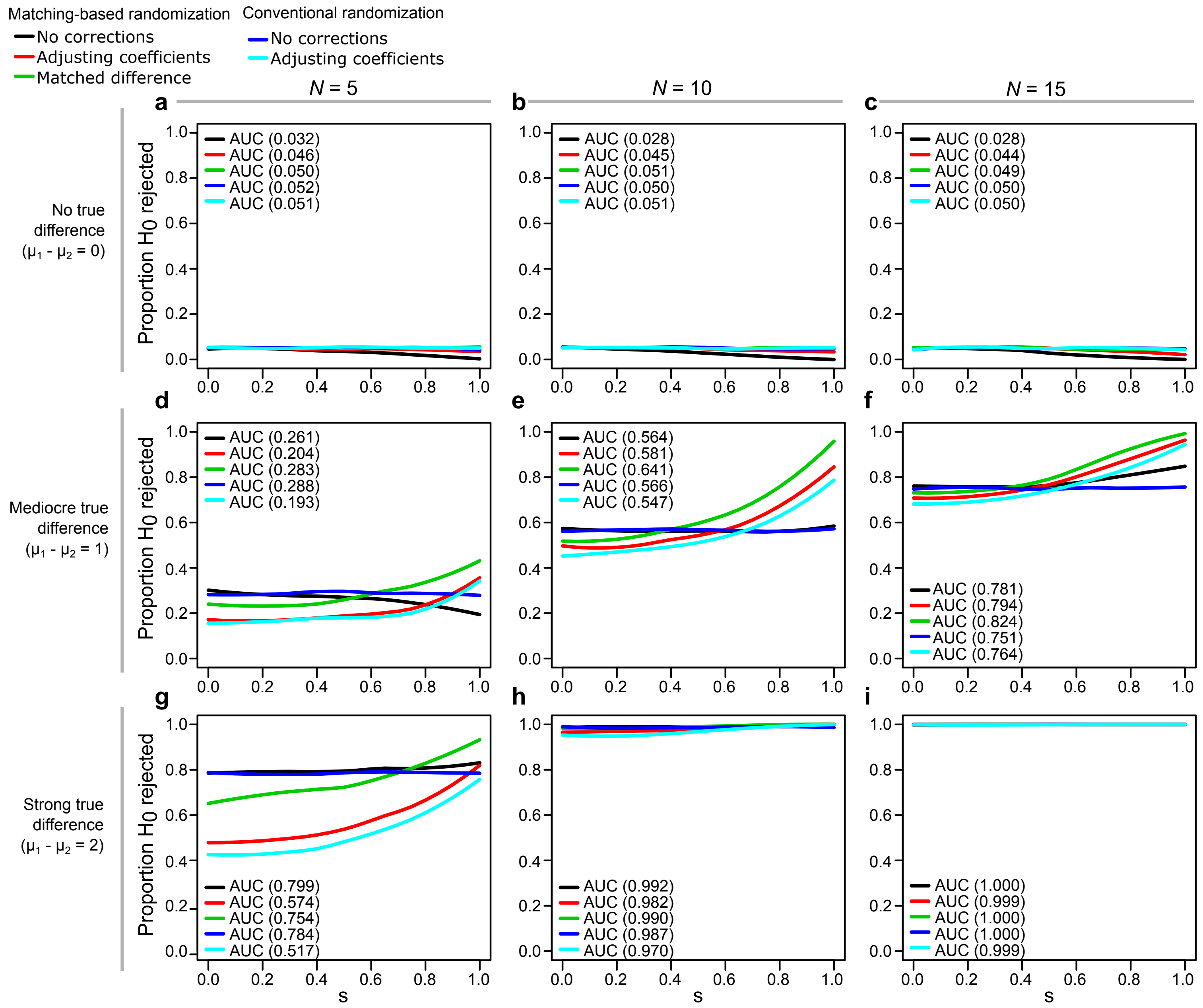

Supplementary Figure S10: Simulation results as a function of the predictive s parameter, with default R loess smoothing applied for the visualization of the curves. Positive detection was defined using the conventional significance threshold of $p < 0.05$ for the multiple regression term to test differences between the two simulated groups. The overall performance of each modeling strategy was assessed with the area under curve (AUC) over the whole range of correlation of the covariate with the outcome (s), which summarizes the findings over the whole correlation spectrum both where there was no predictive baseline information (low s) or where the single covariate had strong predictive power (high s), but was confounded by the three additional random confounder-covariates. The three columns indicated the different sample sizes $N \in \{5, 10, 15\}$. (**a-c**) Simulations when no group difference was present. (**d-f**) Mediocre group difference. (**g-i**) Strong group difference.

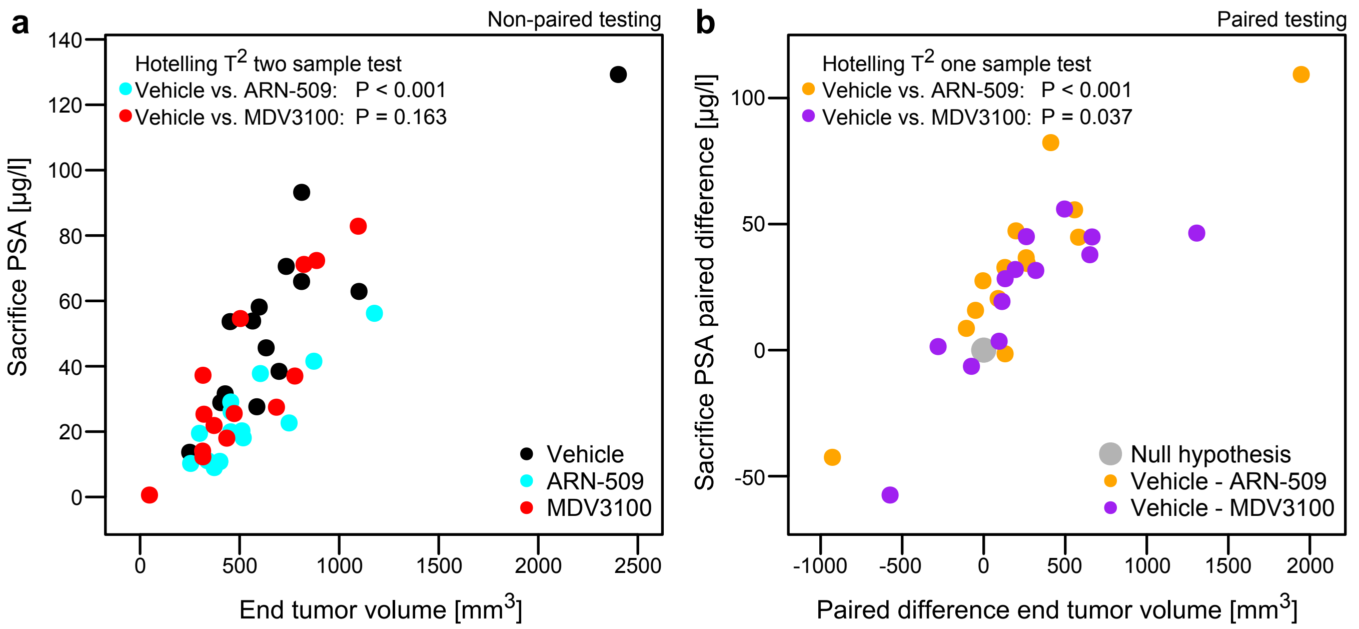

Supplementary Figure S11: A single end-point testing example from the VCaP ARN-509 / MDV3100 -study. Hotelling's T^2 multivariate extension of the t-test was used to illustrate how two end-point markers can be tested with or without the matching information. In this case the two end-point markers were highly correlated, illustrating that the PSA was a feasible surrogate marker to serve as a proxy for the actual tumor size in the orthotopic VCaP animal model. (**a**) In the non-paired case, MDV3100 was to some extent overlapping with the sacrifice measurements from the Vehicle group. (**b**) Pairing the end-point markers and comparing to the null hypotheis that the multivariate normal distribution $\mu = \{0, 0\}$. The paired adjustment revealed difference between Vehicle and MDV3100, which was consistent with the results observed in the longitudinal PSA analysis (Table 1).

Supplementary Table S1. Distance/dissimilarity measures for capturing similarities between two d-dimensional variable vectors $\boldsymbol{x}$ and $\boldsymbol{y}$. The symbol s_i denotes the standard deviation of the associated i:th variable; $\boldsymbol{S}$ denotes the $d \times d$ -dimensional covariance-variance matrix computed between the variables, thus incorporating also inter-variable correlations; R denotes the range of the variable. Some of the measures can be obtained as special cases of Minkowski or Mahalanobis (listed as footnotes).

Distance measure	Formula
Minkowski †	$\left(\sum_{i=1}^{d} \lvert x_i - y_i \rvert^r\right)^{\frac{1}{r}}, r \geq 1$
Mahalanobis	$\sqrt{(\boldsymbol{x}-\boldsymbol{y})\boldsymbol{S}^{-1}(\boldsymbol{x}-\boldsymbol{y})^T}$
Euclidean [a,b,]†	$\sqrt{\sum_{i=1}^{d} (x_i - y_i)^2}$
Standardized Euclidean [c]	$\sqrt{\sum_{i=1}^{d} \left(\frac{x_i}{s_i} - \frac{y_i}{s_i}\right)^2}$
Manhattan [d,]†	$\sum_{i=1}^{d} \lvert x_i - y_i \rvert$
Maximum [e,]†	$\max_{1 \leq i \leq d} \lvert x_i - y_i \rvert$
Gower dissimilarity *	continuous: $\lvert x_i - y_i \rvert / R_i$<br>binary/categorical: 0 if $x_i = y_i$, 1 otherwise

[a] obtained as a special case of Minkowski when $r = 2$; [b] obtained as a special case of Mahalanobis when S is a unit diagonal matrix; [c] obtained as a special case of Mahalanobis when S is a diagonal matrix; [d] obtained as a special case of Minkowski when $r = 1$; [e] obtained as a special case of Minkowski when $r \rightarrow \infty$; † is not scale-invariant, thus data normalization should be considered; * Suitable for mixed-type data. Gower's dissimilarity coefficient is obtained by summarizing over all the available variables i=1,2,..., d.

Supplementary Methods for

Optimized design and analysis of preclinical intervention studies *in vivo*

Teemu D Laajala, Mikael Jumppanen, Riikka Huhtaniemi, Vidal Fey, Amanpreet Kaur, Matias Knuuttila, Eija Aho, Riikka Oksala, Jukka Westermarck, Sari Mäkelä, Matti Poutanen, Tero Aittokallio.

Orthotopic VCaP xenograft in immunodeficient mice: ARN-509 and MDV3100 interventions

We used our recently established orthotopic VCaP xenograft model that enables one to model the characteristics of castration resistant prostate cancer (CRPC) growth *in vivo*, and to study the intratumoral androgen biosynthesis in CRPC[22]. Similar to clinical CRPC, androgen receptor (AR) expression is restored in the VCaP model, despite undetectable serum androgen levels after castration. Furthermore, the AR-mediated signaling continues to play a key role in tumor growth, as indicated by the response to anti-androgen treatments as measured by serum PSA measurements. In the analyzed experiment, we tested the effects two AR antagonists on the castration resistant growth of the VCaP cells. Both of the antiandrogens (MDV3100 and ARN-509) block binding of the endogenous ligand to AR. Furthermore, the MDV3100 promotes also AR degradation, while the ARN-509 especially inhibits the nuclear import and DNA-binding of AR.

The study has been described in detail in[22]. Briefly, adult male immunodeficient mice (HSD: Athymic Nude Foxn 1nu, Harlan Laboratories, 6 to 8 weeks of age) were housed in individually ventilated cages under controlled conditions of light (12h light /12h dark), temperature (21 ±3ºC), and humidity (55% ±15%) in specific pathogen-free conditions at the Central Animal Laboratory, University of Turku. The mice were given irradiated soy-free natural-ingredient feed (RM3 (E), Special Diets Services) and autoclaved tap water *ad libitum*. One million VCaP cells in 20 µl medium were inoculated orthotopically into the dorsolateral prostate through an abdominal incision. Isoflurane (Baxter) was used to induce anesthesia. For pain relief, mice were injected s.c. with buprenorphine (Temgesic, Reckitt Benckiser Healthcare, 0.05-0.1 mg/kg) and carpofen (Rimadyl, Pfizer, 5 mg/kg) before and after the operation, respectively.

Tumor growth was followed by weekly serum prostate specific antigen (PSA) measurements until the mice were sacrificed. Tumors were allowed to grow for 4-5 weeks, until serum PSA had reached at least 5 µg/l in 60% of the animals, and the mean serum PSA value was approximately 15 µg/l. Thereafter, all mice with tumors were castrated within two subsequent weeks (week 4 and 5). This resulted in a dramatic reduction in the serum PSA concentration in all mice, while after a few weeks the castration resistant tumors emerged in 83% of those mice that had increased PSA levels prior to castration. The mice were then allocated to multiple treatment arms while retaining a balance between the groups based on the PSA levels measured at week 10, the change of the PSA level from week 9 to 10, body weights on week 9, cage placement, and the week castration took place. Subsequently, mice were randomized to masked treatment arms, each containing 15 matched animals, in order to guarantee that prognostically comparable mice were available for treatment effect assessment. All the groups were constrained to have an equal number of mice castrated on week 9 or 10, to prevent stratification in respect to this factor. Animals were treated with vehicle or novel antiandrogens of MDV3100 or ARN-509 (20 mg/kg/day). Vehicle and the

antiandrogens were administered by gavage once a day for 28 days. The antiandrogens were synthesized at Orion Pharma (Finland). The vehicle contained 50% PEG300 (Merck KGaA) + 35% 100 mg/ml glucose solution (Baxter) + 10% Tween80 (Merck KGaA) + 5% DMA (Merck KGaA). The study was conducted in accordance with the Animal Experiment Board in Finland (ELLA) for the care and use of animals under the license ESAVI/1993/04.10.03/2011.

Subcutaneous VCaP xenograftin immunodeficient mice: ORX and ORX+Tx interventions

Male immunodeficient mice (Athymic Nude-*Foxn1*^{nu}, Harlan Laboratories, initially 5 to 6 weeks of age) were housed in individually ventilated cages under controlled conditions of light (12h light /12h dark), temperature (22 ±2ºC) and humidity (55% ±15%) in specific pathogen-free conditions at the animal facilities of Orion Corporation, Orion Pharma, Finland. The mice were given irradiated soy-free natural-ingredient feed (RM3 (E), Special Diets Services, England) and filtered, UV treated, tap water *ad libitum*.

Two million VCaP cells in 150 µl of RPMI medium (Gibco®, Life Technologies, Canada) complemented with Matrigel™ (1:2, BD Biosciences, Belgium) were inoculated subcutaneously (s.c.) to the right flanks of the mice. Development of the tumors was monitored by measuring their volume twice a week, and by measuring the serum concentration of prostate specific antigen (PSA) every ten days. The volume of the tumors was calculated according to following formula: $W^2 * L / 2$ (W = shorter diameter, L = longer diameter of the tumor). For PSA measurements, approximately 100 µl of blood was collected by saphenous vein puncture, and the PSA was measured with time-resolved fluorometer (Wallac, PerkinElmer Analytical Life Sciences) as described previously[49]. Reagents for the PSA fluorometric assay were provided by Kim Petterson (University of Turku, Finland). Tumors were allowed to grow for 7 weeks, until the mean volume of the tumors reached approximately 300 mm3, and the mean serum PSA value was approximately 20 µg/l. Animals were randomized in three groups taking into account the animal weight, tumor size and PSA concentration. Two thirds of the animals were castrated and the remaining was left as intact controls. Castrations (ORX and ORX+Tx, where *Tx* is an undisclosed intervention) were carried out under the isoflurane (2-3%, Baxter S.A., Belgium) induced anesthesia. For pain relief, mice were injected s.c. with buprenorphine (0.1 mg/kg, Temgesic® 0.3 mg/ml, Reckitt Benckiser Healthcare Ltd.,United Kingdom) and carpofen (5 mg/kg, Vet Rimadyl® 50 mg/ml, Pfizer SA, Belgium) before and after the operations. Animals were treated with vehicle (2 ml/kg) administered by gavage once a day, from day 50 onwards, until the end of the study (8 weeks). The vehicle contained 0.5 % methylcellulose in water and 0.1 % Tween® 80 (Merck, Germany) solution. The study was conducted in accordance with the Animal Experiment Board in Finland (ELLA) for the care and use of animals under the license ESAVI/7472/04.10.03/2012.

Constrained randomization in the optimal matching

Let N be the number of individuals participating in the experiment to be allocated to G equally sized experimental groups. Lower case letters $g = \{a,b,c, \dots\}$ are used to annotate the groups. Vector $f = \{f_1, f_2, f_3, f_4, \dots\}$ of length N describes allocation per each individual $f_i \in g$, where f_1 is the masked group label given for the 1st individual, and so on. We assume that the desired groups are balanced in size, thus, each label from g occurs in f total N/G times. Since the labels are not fixed to specific actual intervention groups, the annotated labels $a,b,c, \dots$ are interchangeable and only separate class boundaries. As an example, allocation of 4 animals to two groups is done with a vector f of length 4, and $g = \{a,b\}$ where a and b

indicate the masked group labels. Enumerating all the possible combinations for allocating 4 individuals to two groups gives the following list: {*a, a, b, b*}, {*a, b, a, b*}, {*b, a, a, b*}, {*a, b, b, a*}, {*b, a, b, a*}, {*b, b, a, a*}. The solutions {*a, b, b, a*} is equal to {*b, a, a, b*} prior to assigning true treatment labels to the masked labels *g,* which is ideally performed by a person other than the randomizer. Suppose that the optimal matching has resulted in matching of individuals {1,3}. This indicates that individuals 1 and 3 should be allocated to different groups. Similarly, the match {2,4} in the same optimal matching solution suggests that individuals 2 and 4 should be allocated to different groups. Therefore, the allowed allocations are constrained to solutions where $f_1 \neq f_3$ and $f_2 \neq f_4$ based on the optimal matching. This criterion is fulfilled in the following allocations: {*a, a, b, b*}, {*b, a, a, b*}, {*a, b, b, a*}, {*b, b, a, a*}. Out of the above set of 4 feasible solutions, one is finally picked at random with equal probabilities for each instance. This will produce the final chosen allocation vector, which is then given as the masked labels for experimental investigators.

Additional constraints are trivial to add to the above methodology. For instance, in the ARN-509/MDV3100 -experiment, a potential cage effect was normalized out by setting additional constraints in the matching based constraints. This stated that for each pair of individuals *i* and *j* that originated from the same cage, the allowed solutions fulfilled the criterion $f_i \neq f_j$.

Branch and bound algorithm (exact optimization)
The branch and bound steps in the algorithm for global solution are defined as follows:
Branching step: The branching step must implicitly enumerate all possible paths in the branch and bound tree, if global optimum is to be guaranteed. We chose a strategy in which every branching step, the submatches are enumerated for each available un-matched individual (**Supplementary Fig. S7c**). This enumeration is structured so that potentially more similar submatches are prioritized in the tree search. The search tree begins with an empty node, where no individuals have yet been matched (root). Then, at the first step all possible matches that include the first index are enumerated. Similarly further down in the branching tree, always the first free index is enumerated. This spans a search tree that would, if needed, include all possible combinations once.

Bounding step: In order to reduce the size of the search tree (**Supplementary Fig. S7c**), branches of the tree need to be discarded based on an optimistic bounding function. **Supplementary Fig. S7d** presents the concept of a bounding function in a minimization optimization task for a continuous variable *x*, which generalizes to the discrete optimization task at hand. Presumably we know some feasible solution (known local minimum) to the optimization task, which may be used as a starting point. We know the boundary of best possible solutions found in a certain part of the solution space based on a bounding function. For example, in the **Supplementary Fig. S7d**, it is known that the local minimum lies inside $X_3 < x \leq X_4$ along with the value of that local minimum $f(x)$. Based on the bounding functions and our current best known minimum, solutions found from space $x \leq X_1$ may be discarded, as the optimistic bounding functions suggests that the best found solution in this range will not improve our current best known solution. Similarly, solutions found from $X_4 \leq x$ may be discarded. Then, solutions from the space $X_1 < x \leq X_2$ need to be tested, since our bounding function does not guarantee that solutions in this range can be discarded. If the bounding function had been better, i.e. closer to the actual function that is minimized, this range could have been discarded, as it does not truly include a solution better than the current minimum. Furthermore, range $X_2 < x \leq X_3$ needs to be tested based on the bounding function, and in this case the global optimum would be found in this range.

Bounding function is similarly utilized in the branch and bound algorithm to discard large amounts of solutions that cannot improve the current best known matching. After each branching step, a bounding function is computed for each branch through relaxation. For this purpose, the optimization problem in equations (1A-E) is temporarily modified by omitting either the constraint (1B) or (1C). Omission of either constraint causes matches to more than G -1 individuals. For each individual index that has not yet been fixed in the search tree, the corresponding rows and columns in $\boldsymbol{D}$ are checked. Per each row, the G - 1 lowest distances are then picked. These distances are then summed over all the non-fixed rows, which yields the lowest sum of distances available for the non-fixed indices. Notice that due to the relaxation, the found boundary may not be a truly feasible solution to the real optimization task, but it is as low or lower than minima that fulfill the criteria set in equations (1B-C). Therefore it is used as an optimistic bounding function for the branch and bound algorithm.

Genetic algorithm (heuristic optimization)

In order to provide a faster local optimization algorithm in addition to the global optimizing Branch & Bound optimization algorithm, we provided a genetic algorithm inspired by similar usage of this family of methods in improving experimental design[22]. The genetic algorithm (GA) is an open-ended framework for mimicking evolutionary behavior in solving an optimization task. The user provides a desired population size of candidate solutions, and then new generations of solutions are generated using pre-defined evolutionary mechanics. In our application, the fitness of a solution is better if the optimal matching solution has a lower target function value, and thus it is more likely to produce offspring or live to the next generation of solutions. Our algorithm consists of the following evolutionary events (with experimentally observed feasible default tuning parameters):

i. The initial random populations are randomly generated by creating a simple legal matching matrix and randomly permuting the rows and columns of such a legal solution, creating a new randomized legal solution fulfilling constraints set in equations (1A-E).

ii. Point mutations are randomly introduced in each generation by randomly performing either a swap of two rows or two columns in a single existing matching matrix $\boldsymbol{X}$ to randomly chosen individual solutions.

iii. New generations are bred by two parents (two solution matrices $\boldsymbol{X}_1$ and $\boldsymbol{X}_2$) by combining the common identified matches by the parents. Each element $X_{i,j} = 1$ that is shared by both the parents is propagated to the offspring as it is, while the remaining elements where either both parents had $X_{i,j} = 0$ or only one parent had $X_{i,j} = 1$ are randomly permutated to generate the rest of the solution.

iv. Solutions with a worse fitness, i.e. higher optimal target function value in equation (5), are more likely to die per each simulated generation and are replaced by the breeding of new solutions.

Data and code availability

The utilized R-package *hamlet*[25] along with its source code is available in the Comprehensive R Archive Network (CRAN), by typing "*install.packages(hamlet)*" to the R-terminal. After loading the R-package using "*library(hamlet)*", all the measurements for the ARN-509 / MDV3100 –study for the pre-intervention baseline or the post-intervention longitudinal data are fully available from the R-package with commands "*data(vcapwide)*" and *data(vcaplong)*", respectively. Similarly, all the measurements for the ORX / ORX+Tx – study for the pre-intervention baseline or the post-intervention longitudinal data are fully available with the commands "*data(orxwide)*" and "*data(orxlong)*", respectively. The RNA-sequencing files for the ARN-509 / MDV3100 –study have been deposited in the European Nucleotide Archive (ENA) with the identification code PRJEB11552.

Graphical User Interface (GUI)

To enable the wider use of these algorithms, also by researchers without bioinformatics skills or resources, we have implemented the core set of the functions also as part of a web-based graphical user interface (GUI), named *R-vivo*. The web-interface was implemented using the R-Shiny software platform (R-Studio, Inc.), and it is freely accessible at our in-house computer server (http://biomedportal.utu.fi:3838/utu-apps/Rvivo/). In addition to the power calculations, *R-vivo* includes user-friendly options and visualizations both for the pre-intervention analyses (animal matching, randomization and treatment group allocation), as well for the post-intervention analyses (including statistical testing of the treatment effects). In its simplest form, the user can input the raw baseline measurements of animals/tumours, compute local or global matching with or without randomization, and output relevant information such as sub-matches or blinded group labels to be further processed by the experimental investigators. A practical challenge in the sampling-based power calculation is that since the computations are not performed on closed-form parameter distributions, the simulations can be relatively time consuming. However, the user has options for quick pilot simulations to gain insight into the magnitude of the required sample size, and then perform a more detailed simulation once the exact sample numbers are to be proposed. In the GUI, this precision is controlled by the number of bootstrap samples within each sample size (n). Response measurements and baseline variables from the VCaP xenograft experiments are available both within the hamlet R-package and in *R-vivo* interface, for testing purposes.

Simulation study for predictive baseline covariates

In order to examine the effects of matching as a function of the number and type of confounding baseline variables, the following single end-point schema was simulated:

Sample generation

The samples z were drawn from the multivariate normal distribution:

$$Z \sim MVN(\mathbf{0}, \textstyle\sum)$$

where the covariance structure was constructed as follows:

$$\sum = \begin{bmatrix} \mathbf{1} & \boldsymbol{\delta} & \boldsymbol{\delta} & \cdots & \boldsymbol{\delta} & \boldsymbol{\delta} & \mathbf{0} & \mathbf{0} & \mathbf{0} & \cdots & \mathbf{0} & \boldsymbol{s} \\ \boldsymbol{\delta} & \mathbf{1} & \boldsymbol{\delta} & \cdots & \boldsymbol{\delta} & \boldsymbol{\delta} & \mathbf{0} & \mathbf{0} & \mathbf{0} & \cdots & \mathbf{0} & \boldsymbol{s} \\ \boldsymbol{\delta} & \boldsymbol{\delta} & \mathbf{1} & \cdots & \boldsymbol{\delta} & \boldsymbol{\delta} & \mathbf{0} & \mathbf{0} & \mathbf{0} & \cdots & \mathbf{0} & \boldsymbol{s} \\ \vdots & \vdots & \vdots & \ddots & \boldsymbol{\delta} & \boldsymbol{\delta} & \mathbf{0} & \mathbf{0} & \mathbf{0} & \cdots & \mathbf{0} & \boldsymbol{s} \\ \boldsymbol{\delta} & \boldsymbol{\delta} & \boldsymbol{\delta} & \boldsymbol{\delta} & \mathbf{1} & \boldsymbol{\delta} & \mathbf{0} & \mathbf{0} & \mathbf{0} & \cdots & \mathbf{0} & \boldsymbol{s} \\ \boldsymbol{\delta} & \boldsymbol{\delta} & \boldsymbol{\delta} & \boldsymbol{\delta} & \boldsymbol{\delta} & \mathbf{1} & \mathbf{0} & \mathbf{0} & \mathbf{0} & \cdots & \mathbf{0} & \boldsymbol{s} \\ \mathbf{0} & \mathbf{0} & \mathbf{0} & \mathbf{0} & \mathbf{0} & \mathbf{0} & \mathbf{1} & \vdots & \vdots & \cdots & \mathbf{0} & \mathbf{0} \\ \mathbf{0} & \mathbf{0} & \mathbf{0} & \mathbf{0} & \mathbf{0} & \mathbf{0} & \cdots & \mathbf{1} & \mathbf{0} & \cdots & \vdots & \vdots \\ \mathbf{0} & \mathbf{0} & \mathbf{0} & \mathbf{0} & \mathbf{0} & \mathbf{0} & \cdots & \mathbf{0} & \mathbf{1} & \cdots & \mathbf{0} & \mathbf{0} \\ \vdots & \vdots & \vdots & \vdots & \vdots & \vdots & \vdots & \vdots & \vdots & \ddots & \vdots & \vdots \\ \mathbf{0} & \mathbf{0} & \mathbf{0} & \mathbf{0} & \mathbf{0} & \mathbf{0} & \mathbf{0} & \cdots & \mathbf{0} & \cdots & \mathbf{1} & \mathbf{0} \\ \boldsymbol{s} & \boldsymbol{s} & \boldsymbol{s} & \boldsymbol{s} & \boldsymbol{s} & \boldsymbol{s} & \mathbf{0} & \cdots & \mathbf{0} & \cdots & \mathbf{0} & \mathbf{1} \end{bmatrix} \begin{matrix} \vdots \\ \vdots \\ \boldsymbol{q} \\ \vdots \\ \vdots \\ \vdots \\ \vdots \\ \boldsymbol{p} \\ \vdots \\ \vdots \\ \vdots \\ \boldsymbol{y} \end{matrix}$$

Here, the symmetric covariance matrix $\sum$ with dimensions $(q + p + 1) \times (q + p + 1)$ was separated into 3 components to simulate different types of baseline covariates:

i) $\boldsymbol{q}$ predictive rows/columns with cross-covariances $\boldsymbol{\delta}$, unit variance and predictive covariance of magnitude $\boldsymbol{s}$ in connection to the final prediction vector $\boldsymbol{y}$

ii) $\boldsymbol{p}$ non-predictive rows/columns with zero cross-covariances and unit variance

iii) The final simulated prediction vector $\boldsymbol{y}$ which corresponded to the (q+p+1):th dimension in the covariance-variance matrix

The cross-covariance structure in $\boldsymbol{\delta}$ was determined using the Higham method[50] to make $\sum$ the closest possible positive definite covariance matrix, with the objective of being able to sample from the multivariate normal distribution.

Parameter grid

The following set of parameters was varied in the simulations:

1. The number of predictive covariates in the data: $q \in \{1, 3, 10, 20\}$
2. The number of non-predictive covariates in the data: $p \in \{1, 3, 10, 20\}$
3. The prognostic component within the covariance matrix: $s \in \{0, 0.4, 0.7\}$
4. The number of samples drawn from the distribution Z per group: $N \in \{5, 10, 15\}$
5. The introduced between-group difference (two groups): $\mu_1 - \mu_2 \in \{0, 1, 2\}$

Pseudoalgorithm

The following algorithm was then run 1,000 times over the parameter grid with given q, p, s, N and $\mu_1 - \mu_2$:

A. Sample $2N$ observations from the multivariate normal distribution Z specified by $q, p,$ and s
B. Set aside the response vector y from the $(q + p + 1)$:th dimension. The remaining $(q + p)$ dimensional observation matrix was utilized as the baseline matching information.
C. 1) If no matching was utilized, randomly allocate y to 2 sample groups of equal size N using simple permutation
   2) If matching at baseline was utilized, then match based on the $(q + p)$ dimensional confounder matrix and randomly allocate the submatches to 2 sample groups of size N as described in the manuscript
D. Increment the y belonging to different sample groups according to desired $\mu_1 - \mu_2$
E. Perform statistical testing using either non-paired or paired t-testing with statistical significance threshold $\alpha = 0.05$:
   1) If no matching information was to be utilized, perform conventional non-paired t-testing between y belonging to the different group labels
   2) If matching was to be utilized, perform paired t-testing where the matching couples pairs of y that were matched at step C2.
F. Compute the proportion of significant findings over all runs and report the findings in the parameter grid

This led to three different approaches:

i) Matched randomization, paired testing: steps C2 and E2
ii) Matched randomization, non-paired testing: steps C2 and E1
iii) Conventional randomization, non-paired testing: steps C1 and E1

The sampled observations were kept constant for each of the different method approaches and the resulting significant findings are reported in **Supplementary Fig. S9**.

Simulation study for baseline-adjusted or matched regression models

In the simulations showing how the matched inference based on baseline matching of animals differs from a standard multiple regression model that adjusts for baseline differences after interventions, we performed the random allocation according to 1) the matched random allocation and 2) by conventional randomization without using the matching information. We inspected three types of regression models: (*i*) A non-adjusted model that only included a group-difference term and which considered y without any pairing (even if the matching was used in the allocation); (*ii*) A covariate-adjusted multiple regression model where all the covariates were all included as coefficients to compensate for possible baseline differences, and where the non-paired y was modeled as the response. This was run both with and without baseline matching randomization; and (*iii*) A matched model where the baseline matched pairwise differences of y were modeled through the intercept of a simple linear regression model. This resulted in a total of 5 different modeling strategies (**Supplementary Fig. S10**).

In order to simulate a difficult scenario, where the researcher may have to choose between different confounders and may end up including even spurious ones, we simulated the case where 3 out of the 4 baseline covariates were randomly sampled from a standardized normal distribution with no cross-covariance to the outcome whatsoever, while the 4th covariate was sampled to have a given correlation (s) with the outcome y. The outcome y was also sampled from a standardized normal distribution and then shifted according to a matched randomization based group assignment or by conventional randomization. This was similar to the procedure used in above "*Simulation study for predictive baseline covariates*" for $q = 1$ and $p = 3$ with a continuous range of predictive s. A total of 1,000 datasets were simulated in this parameter grid, with known group difference ($\mu_1 - \mu_2 \in \{0,1,2\}$ for none, mediocre, or strong group difference), sample size per group ($N \in \{5,10,15\}$), and with added unit variance for all the covariates (i.e., variance equals to one).

References (continued)

49. Lövgren, T. *et al*. One-step all-in-one dry reagent immunoassays with fluorescent europium chelate label and time-resolved fluorometry. *Clin Chem* **42**: 1196-201 (1996).
50. Higham, N. Computing the nearest correlation matrix - a problem from finance. *IMA J Numer Anal* **22**: 329–43 (2002).

Hamlet R-package: step-by-step user instructions

Teemu Daniel Laajala

March 1, 2016

Contents

1 Introduction **2**
1.1 Analysis workflow 2

2 Pre-intervention analyses **2**
2.1 Loading data into R 2
2.2 Excel format data 4
2.2.1 CSV-files 4
2.3 Distance and dissimilarity functions 5
2.4 Non-bipartite optimal matching of animals at baseline (BB) . . . 6
2.5 Non-bipartite optimal matching of animals at baseline (GA) . . . 7
2.6 Randomization based on matched individuals 9
2.7 Visualizations for pre-clinical data 10

3 Power analysis **13**
3.1 An artificial example 13
3.1.1 Structure of a mixed-effects model 15
3.1.2 Bootstrap simulations 17
3.2 An ARN-509 example 19

4 Post-intervention analyses **22**
4.1 Long format and the presented datasets 22
4.2 Collating to pairwise submatched observations 23
4.3 Fitting conventional and pairwise matched mixed-effects models . 23

1 Introduction

`Hamlet` is an R package intended for the statistical analysis of pre-clinical studies. This document is a basic introduction to the functionality of `hamlet` and a general overview to the analysis workflow of preclinical studies.

This document is structured as follows: First, a general overview to inputting and processing the raw data is presented. Second, functionality is presented for the processing of pre-intervention data. Finally, functionality is presented for the post-intervention period, along with brief discussion on the differences between non-matched and matched statistical approaches. Each section comes with a list of useful functions specific for the subtask.

Latest version of `hamlet` is available in the Comprehensive R Archive Network (CRAN, http://cran.r-project.org/). CRAN mirrors are by default available in the installation of R, and the `hamlet` package is installable using the R terminal command: `install.packages("hamlet")`. This should prompt the user to select a nearby CRAN mirror, after which the installation of `hamlet` is automatically performed. After the `install.packages`-call, the `hamlet` package can be loaded with either command `library("hamlet")` or `require("hamlet")`.

The following notation is used in the document: R commands, package names and function names are written in `typewriter font`. The notation of format `pckgName::funcName` indicates that the function `funcName` is called from the package `pckgName`. If only the function name is given, this indicates that it is located in the base package in R and is thus always available.

1.1 Analysis workflow

Two different types of case-control setups for the analysis of pre-clinical are presented in Fig. 1.

The type A experiment design in Fig. 1 is preferred, as matching is performed before allocation to the experiment groups, and therefore improves the balance and power of the experiment. The alternate experiment type B requires the bipartite matching task, where suitable pairs of individuals are identified over two or more groups that existed prior to matching. This document focuses on experiment design of type A, where similarity information is utilized readily before interventions.

2 Pre-intervention analyses

2.1 Loading data into R

The `hamlet` package comes pre-installed with the VCaP dataset, which is used here to illustrate the workflow. Two different formats of the data are provided. First one is available in `data(vcapwide)`, which includes the data in the so-called *wide* format. In this data format the columns are indicators for different variables available for the experimental unit (here animal). For example, the two first rows of observations are extracted with:

```
> require(hamlet)
> data(vcapwide)
```

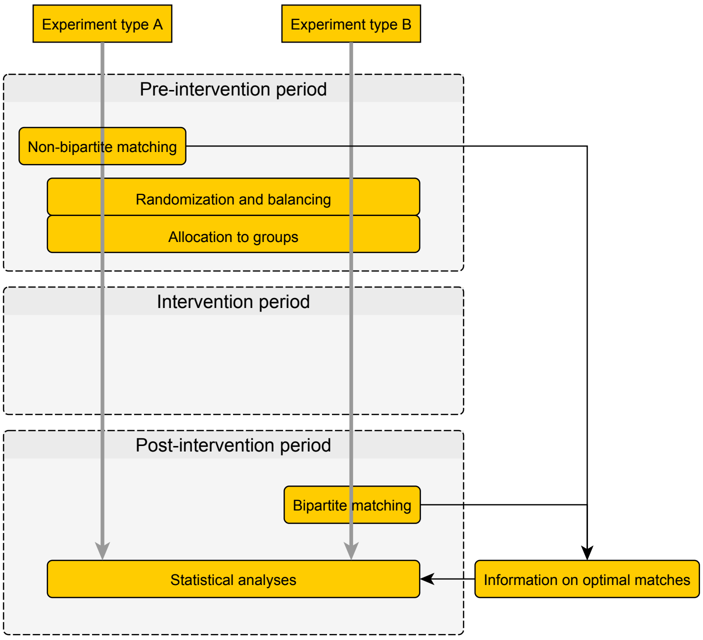

Figure 1: Analysis workflow for pre-clinical experiments

```
> vcapwide[1:2,]

      CastrationDate CageAtAllocation   Group TreatmentInitiationWeek    Submatch
ID003         100413            13489 Vehicle                  Week10  Submatch_1
ID007         170413            13810     MDV                  Week10 Submatch_10
         ID PSAWeek2 PSAWeek3 PSAWeek4 PSAWeek5 PSAWeek6 PSAWeek7 PSAWeek8 PSAWeek9
ID003 ID003     7.67    14.76    24.78     2.03     5.97     8.16    13.72    16.57
ID007 ID007     2.01     5.17     8.59    14.62     1.99     2.81     4.23     5.38
      PSAWeek10 PSAWeek11 PSAWeek12 PSAWeek13 PSAWeek14 BWWeek0 BWWeek1 BWWeek2
ID003     21.30     45.69     54.50     53.55     27.64    30.5    31.7    32.6
ID007      7.55      9.70     17.45     22.79     21.88    28.8    30.0    30.6
      BWWeek3 BWWeek4 BWWeek5 BWWeek6 BWWeek7 BWWeek8 BWWeek9 BWWeek10 BWWeek11
ID003    33.8    33.9    32.2    32.6    32.6    33.2    34.2     35.0     36.1
ID007    31.6    32.9    32.4    32.0    31.1    30.3    30.5     31.6     31.7
      BWWeek12 BWWeek13 BWWeek14
ID003     37.9     37.5     39.7
ID007     32.4     33.5     33.3
```

An another format of the same dataset is provided in `data(vcaplong)`. This is the data from the same experiment in the so-called *long* format, where only few column variables are available (here PSA or body weight), and the different observations belonging to a single experimental unit (here animal) are distinguished using the measurement time (variable *Week* or *DrugWeek*). Again, first few rows of the dataset:

```
> data(vcaplong)
> vcaplong[1:3,]
```

	A	B	C	D
1	Animal	PSA week 10 [ug/l]	PSA week 9 [ug/l]	Body weight week 10 [g]
2	ID003	21,3	16,57	35
3	ID007	7,55	5,38	31,6
4	ID008	23,58	17,4	33,6
5	ID009	13,17	11,14	31,7
6	ID010	9,9	9,33	34,1
7	ID016	15,05	15,29	39,6
8	ID018	13,53	12,14	34
9	ID025	13,13	10,91	33,3
10	ID027	9,59	8,79	32
11	ID031	7,04	6,95	36,6
12	ID032	8,49	8,02	34,9
13	ID037	13,74	13,38	32,4
14	ID040	23,62	19,15	35,9
15	ID045	14,27	9,8	34,8
16	ID047	6,57	6,28	31,9
17	ID054	34,72	27,14	32,1
18	ID056	28,15	22,05	32,2
19	ID058	9,74	7,68	34

Figure 2: Example Excel-format data, where rows correspond to individuals and columns to different characteristics at baseline. The single sheet data can be easily exported in a text-based format such as CSV.

```
     PSA  log2PSA   BW   Submatch    ID Week DrugWeek   Group Vehicle ARN MDV
11 21.30 4.412782 35.0 Submatch_1 ID003   10        0 Vehicle       1   0   0
12 45.69 5.513807 36.1 Submatch_1 ID003   11        1 Vehicle       1   0   0
13 54.50 5.768184 37.9 Submatch_1 ID003   12        2 Vehicle       1   0   0
```

The former *wide* format is useful for summarizing multiple variables when constructing distance matrices for the data. The latter *long* format is typically used for longitudinal mixed-effects modeling where observations are correlated through time.

2.2 Excel format data

An example view of a pre-clinical dataset is given in Fig. 2. Such a dataset can be saved in an R-friendly format by selecting option `File > Save As` and `CSV (Comma delimited)` as the save format in MS Excel.

2.2.1 CSV-files

CSV (Comma Delimited Values) is a suitable text-based format for the data to be read into R using either the function `read.table` or `read.csv`. The above presented example CSV file can be opened with the following command:

```
> ex <- read.table(file="example.csv", sep=";", dec=",", stringsAsFactors=F, header=T)
> ex
```

```
   Animal PSA.week.10..ug.l. PSA.week.9..ug.l. Body.weight.week.10..g.
1   ID003              21.30             16.57                    35.0
```

2	ID007	7.55	5.38	31.6
3	ID008	23.58	17.40	33.6
4	ID009	13.17	11.14	31.7
5	ID010	9.90	9.33	34.1
6	ID016	15.05	15.29	39.6
7	ID018	13.53	12.14	34.0
8	ID025	13.13	10.91	33.3
9	ID027	9.59	8.79	32.0
10	ID031	7.04	6.95	36.6
11	ID032	8.49	8.02	34.9
12	ID037	13.74	13.38	32.4
13	ID040	23.62	19.15	35.9
14	ID045	14.27	9.80	34.8
15	ID047	6.57	6.28	31.9
16	ID054	34.72	27.14	32.1
17	ID056	28.15	22.05	32.2
18	ID058	9.74	7.68	34.0

The above presented CSV file was read into R using `read.table` with the following parameters: `file="example.csv"` is the first parameter and indicates the input file from our current working directory. The working directory may be changed using the command `setwd` or by including its path in the file parameter, i.e. `file="D://my//current//windows//working//directory//example.csv"`. `sep=";"` indicates that the values on each line are separated with the symbol ';', as is the format defined for the CSV delimited files with ","-decimals. This could also be a value such as `\tab` or `" "` (space). `dec=","` indicates that the "," symbol is used for decimals. The default value for indicating decimals is "." otherwise. `stringsAsFactors=F` indicates that strings should not be handled as factors. Factors are an R class, where a character string may only take instances of a predetermined set of strings. As each of our animal IDs - which are read as strings - are unique, it is generally more flexible to conserve them as character strings. Lastly, `header=T` indicates that the text CSV file has a header row as the first row, which includes names for each column. If this value is set to `header=F` or `header=FALSE`, the first row of the text file is read as the first observation and the columns are left unnamed.

Depending on the country of origin, the CSV files may use "." decimals and "," separator, or alternatively (as assumed here) "," decimals" and ";" separators.

List of useful functions:

- `read.table`, `read.csv`
- `data`: `data(vcaplong)`, `data(vcapwide)`

2.3 Distance and dissimilarity functions

A distance or dissimilarity function is used to describe the amount of dissimilarity between two experimental units. Common choices for computing the amount of similarity between two vectors $\mathbf{x}$ and $\mathbf{y}$ include:

- Euclidean distance: $\mathrm{d}(\mathbf{x},\mathbf{y}) = \sqrt{(x_1-y_1)^2+(x_2-y_2)^2+\cdots+(x_n-y_n)^2} = \sqrt{\sum_{i=1}^{n}(x_i-y_i)^2}$.

- Standardized Euclidean distance: $\sqrt{\sum_{i=1}^{P} \frac{(x_i - y_i)^2}{s_i^2}}$
- Mahalanobis distance: $\sqrt{(x-y)^T S^{-1} (x-y)}$

Here, $\mathbf{x}$ and $\mathbf{y}$ are expected to be observation vectors of length P, where each dimension describes the measured value for a particular covariate. S describes the covariance-variance matrix between covariates, and therefore incorporates inter-correlations between variables. The standard deviation s may be used to standardize differences in variation over the dimensions.

	1	2	3	4	5	6	7	8	9	10	11	12	13	14	15	16	17	18
1	0.00	18.05	2.80	10.32	13.53	7.87	9.00	10.08	14.38	17.28	15.40	8.61	3.58	9.76	18.23	17.33	9.21	14.62
2	18.05	0.00	20.14	8.05	5.23	14.78	9.34	8.04	3.99	5.27	4.33	10.15	21.60	8.66	1.36	34.81	26.51	3.98
3	2.80	20.14	0.00	12.29	15.89	10.64	11.35	12.30	16.50	19.79	17.82	10.70	2.89	12.08	20.39	14.87	6.67	16.92
4	10.32	8.05	12.29	0.00	4.44	9.12	2.53	1.62	4.29	8.90	6.47	2.42	13.82	3.55	8.20	26.84	18.54	5.39
5	13.53	5.23	15.89	4.44	0.00	9.61	4.59	3.68	2.19	4.48	2.08	5.83	16.97	4.45	5.02	30.61	22.33	1.66
6	7.87	14.78	10.64	9.12	9.61	0.00	6.60	7.91	11.39	11.95	10.86	7.56	10.10	7.33	14.57	24.16	16.49	10.84
7	9.00	9.34	11.35	2.53	4.59	6.60	0.00	1.47	5.54	8.71	6.57	2.04	12.43	2.58	9.34	26.03	17.75	5.85
8	10.08	8.04	12.30	1.62	3.68	7.91	1.47	0.00	4.33	7.98	5.70	2.70	13.59	2.19	8.15	27.04	18.73	4.73
9	14.38	3.99	16.50	4.29	2.19	11.39	5.54	4.33	0.00	5.57	3.20	6.20	17.87	5.55	3.93	31.12	22.81	2.29
10	17.28	5.27	19.79	8.90	4.48	11.95	8.71	7.98	5.57	0.00	2.48	10.19	20.60	7.98	4.77	34.56	26.32	3.82
11	15.40	4.33	17.82	6.47	2.08	10.86	6.57	5.70	3.20	2.48	0.00	7.91	18.81	6.05	3.96	32.58	24.30	1.58
12	8.61	10.15	10.70	2.42	5.83	7.56	2.04	2.70	6.20	10.19	7.91	0.00	11.96	4.34	10.10	25.09	16.82	7.14
13	3.58	21.60	2.89	13.82	16.97	10.10	12.43	13.59	17.87	20.60	18.81	11.96	0.00	13.27	21.73	14.19	6.53	18.11
14	9.76	8.66	12.08	3.55	4.45	7.33	2.58	2.19	5.55	7.98	6.05	4.34	13.27	0.00	8.95	26.95	18.69	5.07
15	18.23	1.36	20.39	8.20	5.02	14.57	9.34	8.15	3.93	4.77	3.96	10.10	21.73	8.95	0.00	35.04	26.73	4.05
16	17.33	34.81	14.87	26.84	30.61	24.16	26.03	27.04	31.12	34.56	32.58	25.09	14.19	26.95	35.04	0.00	8.31	31.72
17	9.21	26.51	6.67	18.54	22.33	16.49	17.75	18.73	22.81	26.32	24.30	16.82	6.53	18.69	26.73	8.31	0.00	23.42
18	14.62	3.98	16.92	5.39	1.66	10.84	5.85	4.73	2.29	3.82	1.58	7.14	18.11	5.07	4.05	31.72	23.42	0.00

Table 1: Euclidean distance matrix D for 18 animals

Table 1 shows the Euclidean distance matrix for the 18 animals presented in Figure 2.

List of useful functions:

- `dist` includes many common distance and dissimilarity functions (Euclidean by default, others: `method="manhattan"`, `method="maximum"`, `method="minkowski"`
- `cluster::daisy`, daisy includes Gower's dissimilarity for mixed data (parameter `metric="gower"`)

2.4 Non-bipartite optimal matching of animals at baseline (BB)

The non-bipartite optimal matching problem may be solved using the provided branch and bound algorithm:

```
> sol.bb <- match.bb(d, g=3)
```

```
[1] "Performing initial sorting for a good initial guess"
[1] "Computing boundaries for minimum distances in possible combinations..."
[1] "Starting branch and bound"
[1] "Branches: 272"
[1] "Bounds: 7140"
[1] "Ends visited: 25"
```

```
[1] "Solution cost 169.62"
[1] "Solution: 5,3,5,6,4,5,6,4,3,2,2,6,1,4,3,1,1,2"

> submatches <- paste("Submatch_", LETTERS[1:6][sol.bb$solution], sep="")
> names(submatches) <- names(sol.bb$solution)
> submatches

          1           2           3           4           5           6
"Submatch_E" "Submatch_C" "Submatch_E" "Submatch_F" "Submatch_D" "Submatch_E"
          7           8           9          10          11          12
"Submatch_F" "Submatch_D" "Submatch_C" "Submatch_B" "Submatch_B" "Submatch_F"
         13          14          15          16          17          18
"Submatch_A" "Submatch_D" "Submatch_C" "Submatch_A" "Submatch_A" "Submatch_B"
```

The `match.bb` function returns the solution to the optimal matching task. It takes as input a distance matrix `d`, as is indicated in the function call `match.bb(d, g=3)` (notice that `d` was defined before). Furthermore, the size of the submatches is defined using the parameter `g=3`. This value indicates that the optimal matching algorithm minimizes edges within triplets. Each observation has to belong to a triplet called a submatch.

List of useful functions:

- Multigroup non-bipartite matching: `hamlet::match.bb`
- Paired non-bipartite matching: `hamlet::match.bb`, `nbpMatching::nonbimatch`
- Paired bipartite matching: `optmatch::fullmatch`

2.5 Non-bipartite optimal matching of animals at baseline (GA)

While the above described Branch and Bound algorithm is guaranteed to identify the global optimum, in some cases it is not feasible due to size of the search tree. In such cases, a feasible optimum is easily detected using a Genetic Algorithm implemention provided in `hamlet::match.ga`.

The Genetic Algorithm (GA) commonly includes many parameters, as it aims to mimic evolutionary processes in solving a problem, here a non-bipartite multigroup matching problem. The basic parameters that should be considered include `generations`, which indicates for how many generations the simulation is run for and thus increases run time approximately linearly, and the parameter `popsize`, which indicates how many solutions should be "living" inside the whole population at a given generation. A thumb rule is that many solutions are easily solvable by the 1,000th generation, if the population size is at least 100, but the user may want to use the visualizations and diagnostic plots to see how well the GA has managed to solve the optimization problem. The convergence happens over the generations similarly as presented in Figure 3.

```
> sol.bb[["cost"]] # Guaranteed global optimum

[1] 169.62

> sol.ga[[3]] # Identified solution by GA
```

```
> set.seed(1) # GA is a stochastic algorithm, fixing the seed for reproducibility
> sol.ga <- match.ga(d, g=3, generations=100, popsize=100)

[1] "Best found solution vector:"
 [1] 5 6 5 3 1 5 1 3 6 2 2 3 4 1 6 4 4 2
[1] "Best found solution cost:"
[1] 171.72
```

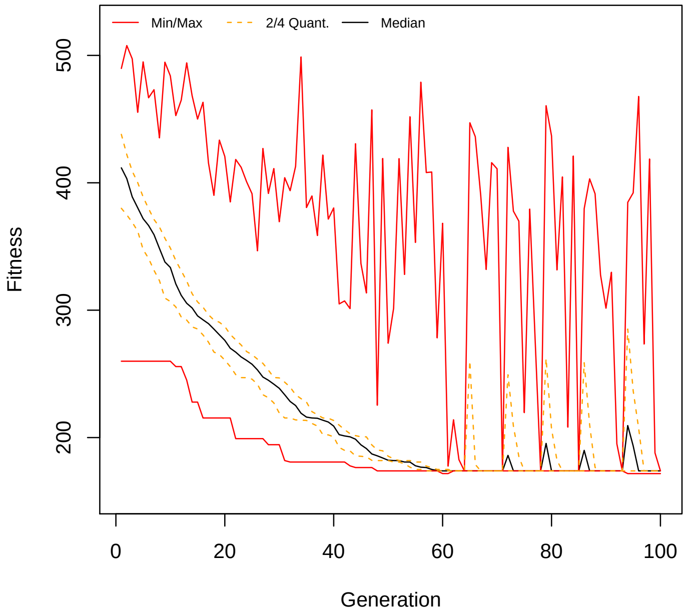

Figure 3: Convergence of the GA in the given optimization problem. The minimum shows the best identified optimization solution, while the quantiles give insight to the solution heterogeneity living in the solution space.

```
[1] 171.72
```

The GA algorithm, with a linear run time, has resulted in a very close optimum to the guaranteed global optimum identified using BB, which may in return have some cases lead to drastic increases in run times. Both algorithms may thus be applicable where appropriate.

2.6 Randomization based on matched individuals

The submatches identified in the above section should not be mistaken for the randomly allocated intervention groups. The final intervention groups are obtained by dividing members of each submatch in the found solution to a separate treatment arm. Since the within-submatch distances are minimized, this guarantees that comparable individuals are randomly divided to separate arms:

```
> ex[,"Submatch"] <- submatches
> set.seed(1) # for reproducibility
> ex[,"AllocatedGroups"] <- match.allocate(ex[,"Submatch"])
> ex <- ex[order(ex[,"Submatch"]),] # Sort for submatches
```

	Animal	PSA.week.10..ug.l.	PSA.week.9..ug.l.	Body.weight.week.10..g.	Submatch	AllocatedGroups
13	ID040	23.62	19.15	35.90	Submatch_A	Group_B
16	ID054	34.72	27.14	32.10	Submatch_A	Group_C
17	ID056	28.15	22.05	32.20	Submatch_A	Group_A
10	ID031	7.04	6.95	36.60	Submatch_B	Group_C
11	ID032	8.49	8.02	34.90	Submatch_B	Group_A
18	ID058	9.74	7.68	34.00	Submatch_B	Group_B
2	ID007	7.55	5.38	31.60	Submatch_C	Group_C
9	ID027	9.59	8.79	32.00	Submatch_C	Group_A
15	ID047	6.57	6.28	31.90	Submatch_C	Group_B
5	ID010	9.90	9.33	34.10	Submatch_D	Group_A
8	ID025	13.13	10.91	33.30	Submatch_D	Group_C
14	ID045	14.27	9.80	34.80	Submatch_D	Group_B
1	ID003	21.30	16.57	35.00	Submatch_E	Group_A
3	ID008	23.58	17.40	33.60	Submatch_E	Group_C
6	ID016	15.05	15.29	39.60	Submatch_E	Group_B
4	ID009	13.17	11.14	31.70	Submatch_F	Group_C
7	ID018	13.53	12.14	34.00	Submatch_F	Group_B
12	ID037	13.74	13.38	32.40	Submatch_F	Group_A

Table 2: The result table in variable `ex` after performing the optimal matching and allocation.

As is seen Table 2, each submatch (column `Submatch`) consists of similar experimental units in terms of the baseline characteristics (i.e. PSA and body weight). Furthermore, the baseline data has now been allocated in such a manner, that each submatch evenly distributes to the proposed intervention groups (column `AllocatedGroup`), resulting in balanced baseline intervention groups. These artificial labels `A`, `B`, and `C` may then be given to an external experimenter in a blinded manner, and allocated to the true labels in any fashion without any pre-fixed control group, as all pairwise contrasts have been considered in the submatching procedure.

List of useful functions:

- Multigroup non-bipartite matching: `hamlet::match.bb`, `hamlet::match.ga`

```
> boxplot(PSA.week.10..ug.l. ~ AllocatedGroups, data = ex, range=0,
+ xlab="Group", ylab="PSA week 10 ul/g")
```

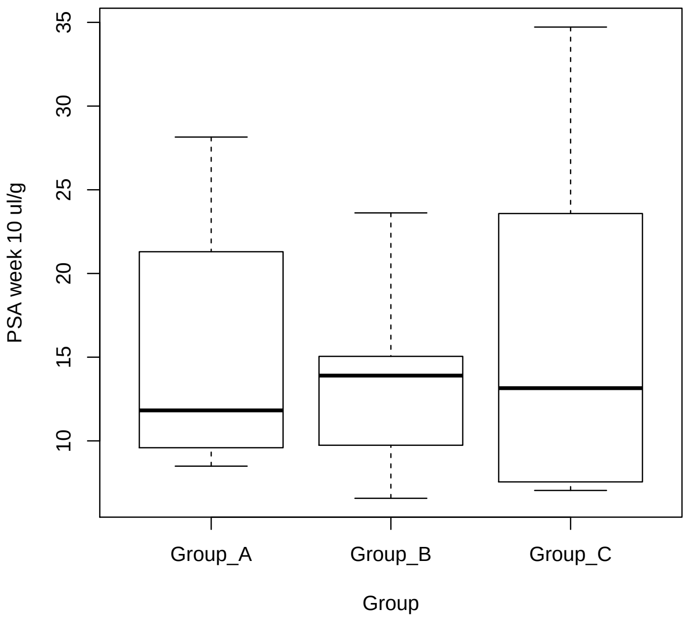

Figure 4: Boxplots for the week 10 PSA in the example allocation

- Paired non-bipartite matching: `hamlet::match.bb`, `hamlet::match.ga`, `nbpMatching::nonbimatch`
- Paired bipartite matching: `optmatch::fullmatch`

2.7 Visualizations for pre-clinical data

Various visualization functions are available to illustrate baseline balance. For example, the boxplots in respect to allocation groups can be plotted using a command such as `boxplot`, which is illustrated in Figure 4.

Mixed variable scatterplots with annotations for the submatches or allocation groups are plotted using the function `hamlet::mixplot`, which can be seen in Figures 5 or 6 respectively.

List of useful functions:

- Scatterplots etc: `hamlet::mixplot`, `plot`, `boxplot`
- Heatmaps: `hamlet::hmap`, `heatmap`, `gplots::heatmap.2`

```
> mixplot(ex[,2:5], pch=16)
```

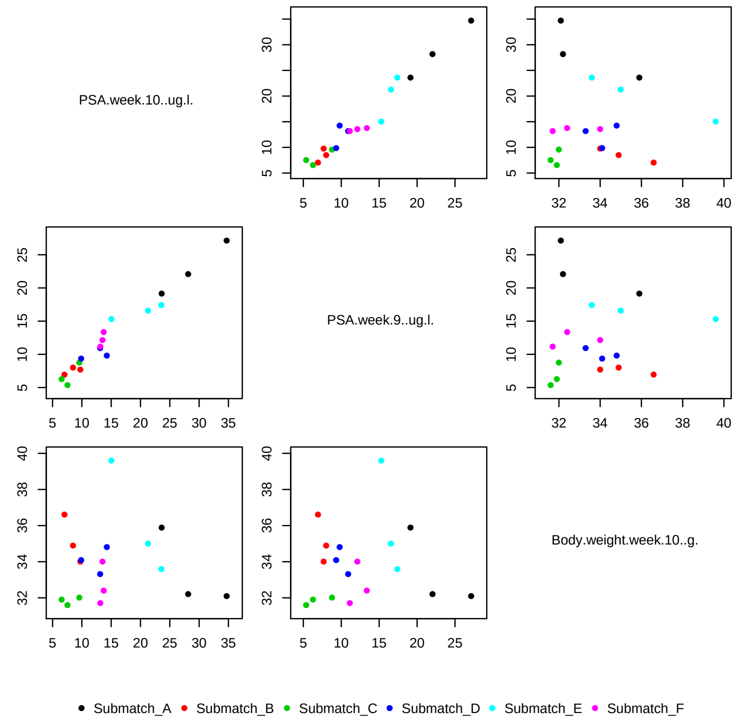

Figure 5: Test mixplot with submatch labels

```
> mixplot(ex[,c(2:4,6)], pch=16)
```

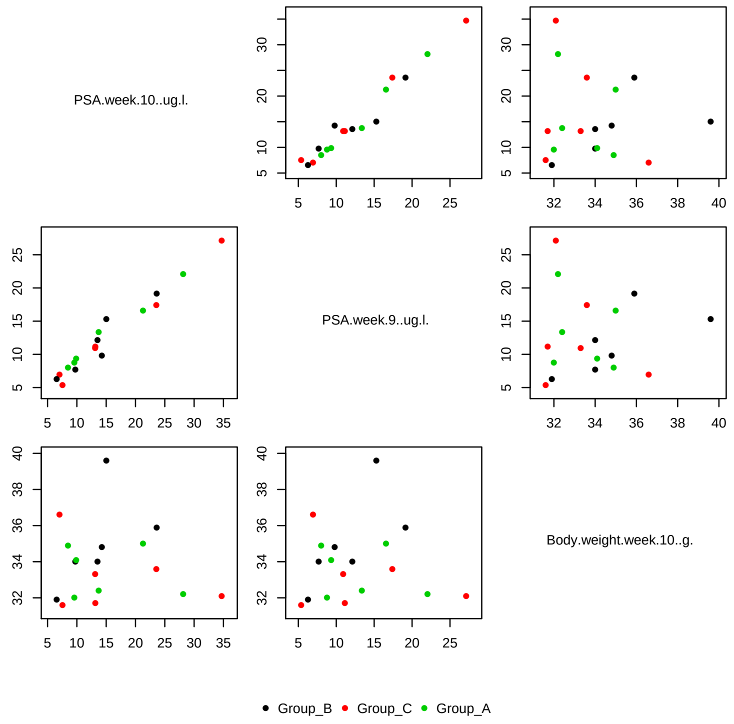

Figure 6: Test mixplot with allocation group labels

3 Power analysis

The power simulations provided by the `hamlet`-package are conducted through bootstrap (sampling with replacement) simulations using a pre-fitted mixed-effects model. For this purpose, it is essential that the user pre-defines a suitable mixed-effects model in the `lmer`-function of the `lme4`-package, as this will be used in the sampling process. The function `mem.powersimu` is the main `hamlet` function that performs this sampling, and it automatically identifies the suitable experimental unit from the `lme4`-object, and then re-fits the model structure a pre-defined amount of times at given N values.

3.1 An artificial example

In a situation where the user wishes to generate artificial data, it is important for the experimenter to evaluate such factors as:

- How many measurement points time will be available
- What is the expected effect size
- Will right-censoring (death or sacrifice) occur and what are the risk criteria
- Are there baseline differences or is there a correlation between the initial baselinse response level and intervention efficacy

The user is encouraged to creatively produce such expert curated data either by hand, or through a tailored simulation function. As a practical example, an example function will be constructed below (mainly utilizing normal distributions). In order for the artificial data to be modeled using `lme4`-package, it should follow the long format.

As an example, data with an initial baseline level of response values with $\mu = 5$ and $\sigma = 2$ will be generated from the normal distribution. 4 follow-up time points will be available after the initial baseline, and the expected control growth will be 2 per time point and in turn the intervention effect to have an effect of -1 to growth per time point. Furthermore, we simulate a right-censoring that has 20% chance to occur for individuals reaching above response values > 10. In this artificial example, 5 individuals will be available for both the control and the intervention group. Each measurement will have measurement error with no bias ($\mu = 0$) and $\sigma = 2$.

```
> # Baseline characteristics and time follow-up
> basemu <- 5
> basesigma <- 2
> ttime <- 4
> # Growth characteristics and group size
> growth <- 2
> interv <- -1
> ngroup <- 5
> # Measurement error and right-censoring
> measerror <- 2
> censthreshold <- 10
> censchance <- 0.2
```

```
> # Artificial data simulation with a set seed
> set.seed(1)
> # 2 experiment groups
> artdat <- do.call("rbind", lapply(c("Control", "Intervention"), FUN=function(group){
+  # Simulated individuals
+  do.call("rbind", lapply(1:ngroup, FUN=function(i){
+   # Baseline time = 0 and 5 follow up points
+   y <- rnorm(n = 1, mean=basemu, sd = basesigma)
+   # Growth as a function of time, with a possible intervention effect
+   measurements <- unlist(lapply(0:ttime, FUN=function(t){
+    y + growth*t + ifelse(group=="Intervention", interv*t, 0)
+   }))
+   # Random chance of censoring for response above >10,
+         # 20\% chance per time point to right-censor
+   for(index in 1:length(measurements)){
+    if(!is.na(measurements[index]) & measurements[index]>censthreshold)
+    if(rbinom(n=1, size=1, prob=censchance))
+         measurements[index:(length(measurements))] <- NA
+   }
+   # Add random measurement error
+   measurements <- measurements +
+    rnorm(n=length(measurements), mean=0, sd=measerror)
+         # Collect all data to a long format data.frame
+   data.frame(
+         Response = measurements,
+         ID = paste(group, i, sep="_"),
+         Group = ifelse(group=="Intervention", 1, 0),
+         Time = 0:ttime)
+  }))
+ }))
```

The above generated simulation script captures some key elements in a preclinical longitudinal intervention study, but should be naturally refined more precisely if more complex interactions are to be incorporated. To give insight into the overall structure of the long-format data, here are the so-called `head` and `tail` of the artificially generated `data.frame`:

```
> head(artdat)

  Response        ID Group Time
1 6.406691 Control_1     0    0
2 8.291951 Control_1     0    1
3 8.576375 Control_1     0    2
4 6.667192 Control_1     0    3
5 9.889958 Control_1     0    4
6 9.219866 Control_2     0    0

> tail(artdat)

   Response             ID Group Time
45 7.593970 Intervention_4     1    4
```

```
46 6.856897 Intervention_5     1    0
47 7.117348 Intervention_5     1    1
48 6.258141 Intervention_5     1    2
49 8.907789 Intervention_5     1    3
50 8.509502 Intervention_5     1    4
```

After a suitable long-format data.frame has been generated (variable `artdat` here), one has to specify and fit a preliminary mixed-effects model that will be used as a base for power simulations. The generated artificial data is presented in Figure 7.

3.1.1 Structure of a mixed-effects model

A standard mixed-effects model would, for example, include the following coefficients, given the input data `artdat` (the formula coefficients need to corresponds to column names in the input `data.frame`):

```
> f1a <- as.formula(Response ~ 1 + Time + Time:Group + (1 + Time|ID))
> f1b <- as.formula(Response ~ 1 + Time + Time:Group + (1|ID) + (0 + Time|ID))
```

This formula is structured as follows:

- The left hand side `Response` is our response vector **y**.
- The non-parenthesis coefficients following the tilde are the so called *fixed effects*, which are here population-wise parameters
- The first right hand side coefficient `1` stands for standard model intercept, i.e. y level when $x = 0$
- Coefficient `Time` captures natural growth of the tumors as a function of time
- Coefficient `Time:Group` introduces grouping information as an interaction with the growth coefficient, and thus tests whether the intervention gives a growth inhibition advantage.
- The terms in parenthesis are *random effects* with analogous counterparts to their *fixed effects*. The difference is that the grouping variable, indicated here with `|ID`, is gives flexibility for the each experimental unit to have deviating intercepts and growth slopes. Separate value from a normal distribution with mean 0 and an estimated standard deviation are identified when fitting the mixed-effects model. The *random effects* allow individualized response curves, while controlling that multiple observations belong to a single individual (`ID`).
- An alternate non-correlated random-effects structure is given in `f1b`, indicated by separating the two *random effects* terms without a cross-correlation.

Fixed effects are typically utilized in inference of possible intervention effects, and here the term `Time:Group` will estimate possible intervention effects. A linear mixed-effects model of the above structure can be fitted using:

```
> # Plot the artificial data
> plot.new()
> plot.window(xlim=range(artdat[,"Time"]),
+         ylim=c(0,max(artdat[,"Response"], na.rm=T)))
> axis(1); axis(2); box()
> title(xlab="t", ylab="y", main="Artificially generated data")
> # Plot each individual as its own curve
> invisible(by(artdat, INDICES=artdat[,"ID"], FUN=function(z){
+         points(z[,"Time"], z[,"Response"], type="l", col=1+z[1,"Group"])
+         points(z[,"Time"], z[,"Response"], pch=16, col=1+z[1,"Group"])
+ }))
> legend("bottomright", col=1:2, pch=16, lwd=1, legend=c("Control", "Intervention"))
```

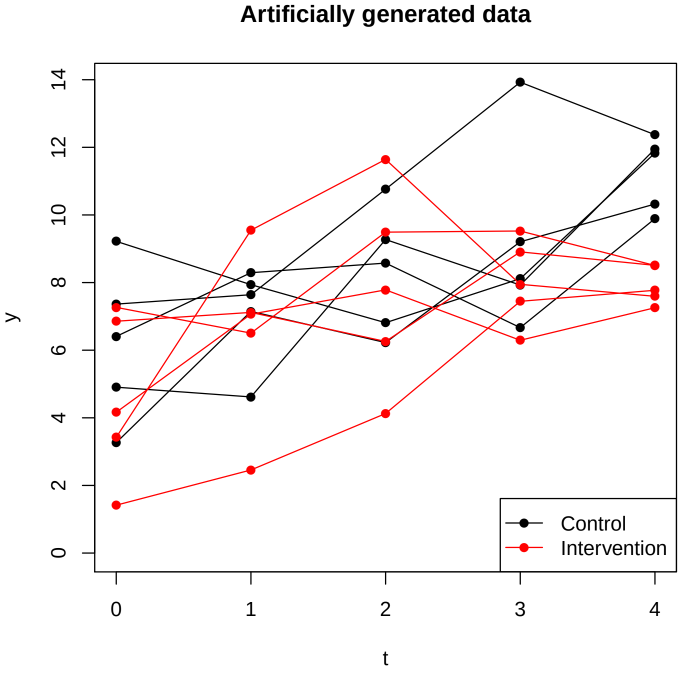

Figure 7: Visualization of the artificially generated data, with two experimental groups.

```
> library(lme4)
> # We defined formulae already before
> fit1 <- lmer(f1b, data = artdat)
> library(lmerTest)
> summary(fit1)

Linear mixed model fit by REML ['lmerMod']
Formula: Response ~ 1 + Time + Time:Group + (1 | ID) + (0 + Time | ID)
   Data: artdat

REML criterion at convergence: 204.8

Scaled residuals:
    Min      1Q  Median      3Q     Max
-1.6891 -0.6997  0.1232  0.5499  2.3074

Random effects:
 Groups   Name        Variance Std.Dev.
 ID       (Intercept) 1.207    1.099
 ID.1     Time        0.000    0.000
 Residual             2.830    1.682
Number of obs: 50, groups:  ID, 10

Fixed effects:
            Estimate Std. Error t value
(Intercept)   5.6894     0.5390  10.556
Time          1.2732     0.2135   5.964
Time:Group   -0.5251     0.2629  -1.998

Correlation of Fixed Effects:
           (Intr) Time
Time       -0.492
Time:Group  0.000 -0.616
```

3.1.2 Bootstrap simulations

The package `lmerTest` is used to provide Satterthwaite approximation for the p-values for fixed effects in the linear mixed-effects model. Albeit the p-values are provided here for the model coefficients, we are interested in how power in such a study would develop as a function of animal numbers N. For this purpose we can perform power simulations, which bootstraps the pre-fitted mixed-effects model on our artificial data:

Notice that the artificial data simulations were run with a very limited bootstrap sample size, in order to save time in generation of this vignette. A better estimate for the power as well as more exact N would be given e.g. by setting `boot=1000` and `N=3:20`. Furthermore, we indicated with `level` that our experimental unit is defined by the individual indicator `ID`, and that we want to subsample evenly over the intervention groups through `strata`. The resulting power curve is shown in Figure 8, suggesting that in order to achieve sufficient statistical power, our experiment should include at least $N_{arm} = 9$ individuals

```
> set.seed(1)
> pow <- mem.powersimu(fit1,
+         N=c(3, 5, 7, 9, 11, 13, 15), boot=20,
+         level="ID", strata="Group")
> abline(h=0.8, col="grey")
> pow

                    (Intercept) Time Time:Group
GroupN_3_TotalN_6             1    1       0.35
GroupN_5_TotalN_10            1    1       0.25
GroupN_7_TotalN_14            1    1       0.70
GroupN_9_TotalN_18            1    1       0.80
GroupN_11_TotalN_22           1    1       0.80
GroupN_13_TotalN_26           1    1       1.00
GroupN_15_TotalN_30           1    1       0.95
```

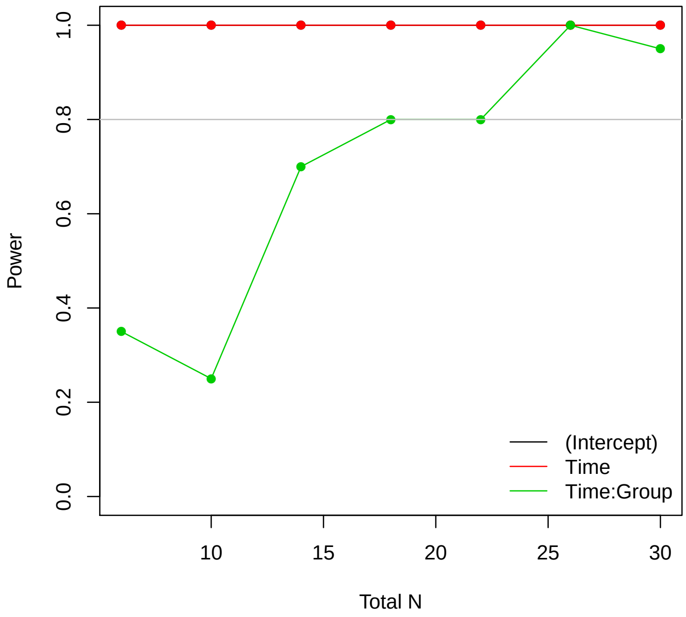

Figure 8: Preliminary power curve for all the fixed effects, with a limited number of bootstrapped datasets (20).

in both intervention arms, and total experiment size consisting of $N_{total} = 18$ individuals.

3.2 An ARN-509 example

The longitudinal intervention observations of the ARN-509 / MDV3100 -study and ORX / ORX+Tx -study are provided inside the `hamlet`-package with the commands `data(vcaplong)` and `data(orxlong)`, respectively. Here we will provide a model fit to the ARN-509 -study, as well as show how its power curve behaves in respect to different *fixed effects*. Load the ARN-509 / MDV3100 -study and constraint to ARN-509 by:

```
> data(vcaplong)
> arndat <- vcaplong[
+         # Select observations only from the vehicle or ARN-509 groups
+         vcaplong[,"Group"] %in% c("Vehicle", "ARN"),
+         # Select columns (=features) that are required for the conventional MEM
+         c("PSA", "DrugWeek", "ARN", "ID")]
> head(arndat)

     PSA DrugWeek ARN    ID
11 21.30        0   0 ID003
12 45.69        1   0 ID003
13 54.50        2   0 ID003
14 53.55        3   0 ID003
15 27.64        4   0 ID003
71 13.17        0   0 ID009
```

Similarly as for the artificial data example, this study could be be representative for estimating power for interventions with similar effect sizes, censoring, follow-up periods etc. The conventional non-matched modeling process and corresponding preliminary power curve would be computed using:

```
> arnfit <- lmer(PSA ~ 1 + DrugWeek + DrugWeek:ARN + (1|ID) + (0 + DrugWeek|ID),
+         data = arndat)
> summary(arnfit)

Linear mixed model fit by REML t-tests use Satterthwaite approximations to degrees
  of freedom [lmerMod]
Formula: PSA ~ 1 + DrugWeek + DrugWeek:ARN + (1 | ID) + (0 + DrugWeek |      ID)
   Data: arndat

REML criterion at convergence: 1082.6

Scaled residuals:
    Min      1Q  Median      3Q     Max
-3.4768 -0.3911 -0.0044  0.3425  3.1437

Random effects:
 Groups   Name        Variance Std.Dev.
 ID       (Intercept) 67.80    8.234
```

```
 ID.1     DrugWeek    26.65    5.163
 Residual             33.05    5.749
Number of obs: 150, groups:  ID, 30

Fixed effects:
             Estimate Std. Error     df t value Pr(>|t|)
(Intercept)    14.311      1.709 29.587   8.374 2.68e-09 ***
DrugWeek       10.062      1.407 28.206   7.150 8.47e-08 ***
DrugWeek:ARN   -7.627      1.982 27.792  -3.849 0.000636 ***
---
Signif. codes:  0 Ś***Š 0.001 Ś**Š 0.01 Ś*Š 0.05 Ś.Š 0.1 Ś Š 1

Correlation of Fixed Effects:
            (Intr) DrugWk
DrugWeek    -0.092
DrugWek:ARN  0.000 -0.704
```

As is seen in Figure 9, the power curves become smoother with higher bootstrap rates. Here a highly narrowed N vector was tested (values 5 to 9), due to a priori knowledge that the power 0.8 would be achieved at $N = 7$.

```
> set.seed(123)
> arnpow <- mem.powersimu(arnfit,
+         level = "ID", strata = "ARN",
+         N = c(5,6,7,8,9), boot = 100)
> abline(h=0.8, col="grey")
> arnpow
```

```
                   (Intercept) DrugWeek DrugWeek:ARN
GroupN_5_TotalN_10           1     0.98         0.68
GroupN_6_TotalN_12           1     1.00         0.75
GroupN_7_TotalN_14           1     1.00         0.90
GroupN_8_TotalN_16           1     1.00         0.83
GroupN_9_TotalN_18           1     1.00         0.93
```

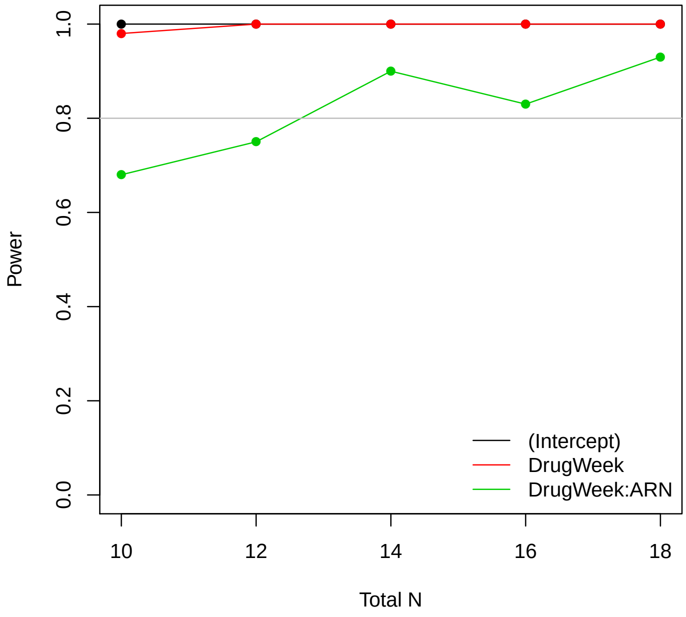

Figure 9: ARN-509 fixed effects power curves, estimated using the conventional model with 100 bootstrapped data sets.

4 Post-intervention analyses

The presented pre-intervention submatching procedure provides a unique opportunity to utilize this predictive power to improve accuracy in the post-intervention inference. We here provide the datasets from both the *ARN-509 / MDV3100* -study and the *ORX / ORX+Tx* -study, by typing `data(vcaplong)` and `data(orxlong)`, respectively. Alternatively, the pre-intervention data is also available in `data(vcapwide)` and `data(orxwide)`, respectively.

4.1 Long format and the presented datasets

As out-lined before, in order to perform regression modeling, R requires the observations to be in the so-called long format, where each row in a data.frame corresponds to a single measurement. These measurements are then usually uniquely defined using individual identification codes as well as time points. The presented datasets:

```
> data(vcaplong)
> data(orxlong)
> head(vcaplong)

     PSA  log2PSA   BW    Submatch    ID Week DrugWeek   Group Vehicle ARN MDV
11 21.30 4.412782 35.0  Submatch_1 ID003   10        0 Vehicle       1   0   0
12 45.69 5.513807 36.1  Submatch_1 ID003   11        1 Vehicle       1   0   0
13 54.50 5.768184 37.9  Submatch_1 ID003   12        2 Vehicle       1   0   0
14 53.55 5.742815 37.5  Submatch_1 ID003   13        3 Vehicle       1   0   0
15 27.64 4.788686 39.7  Submatch_1 ID003   14        4 Vehicle       1   0   0
41  7.55 2.916477 31.6 Submatch_10 ID007   10        0     MDV       0   0   1
```

```
> head(orxlong)

   ID   PSA    log2PSA Day TrDay       Date  Group    Submatch ORXTx ORX Intact
1 ID1 0.368 -1.4422223   0   -10 2015-01-12 ORX+Tx Submatch_11     1   0      0
2 ID1 1.524  0.6078629  10     0 2015-01-22 ORX+Tx Submatch_11     1   0      0
3 ID1 0.034 -4.8783214  25    15 2015-02-06 ORX+Tx Submatch_11     1   0      0
4 ID1 0.100 -3.3219281  35    25 2015-02-16 ORX+Tx Submatch_11     1   0      0
5 ID1 0.203 -2.3004484  45    35 2015-02-26 ORX+Tx Submatch_11     1   0      0
6 ID1 0.357 -1.4860040  56    46 2015-03-09 ORX+Tx Submatch_11     1   0      0
```

Typically, an experimenter may model a single interesting contrast with a single model, thus we will split the `vcaplong` and `orxlong` into two separate data sets.

```
> # Interesting fields in the orthotopic VCaP study
> fields <- c('PSA', 'DrugWeek', 'ID', 'Submatch', 'Group', 'ARN', 'MDV')
> # ARN-509 vs Vehicle
> arndat <- vcaplong[vcaplong[,'Group'] %in% c('ARN', 'Vehicle'),fields]
> # MDV3100 vs Vehicle
> mdvdat <- vcaplong[vcaplong[,'Group'] %in% c('MDV', 'Vehicle'),fields]
> # Interesting fields in the subcutaneous VCaP study
> fields <- c('PSA', 'TrDay', 'ID', 'Submatch', 'Group', 'ORXTx', 'ORX')
```

```
> # ORX vs Intact
> orxdat <- orxlong[orxlong[,'Group'] %in% c('ORX', 'Intact'), fields]
> # ORX+Tx vs ORX
> xtxdat <- orxlong[orxlong[,'Group'] %in% c('ORX+Tx', 'ORX'), fields]
```

4.2 Collating to pairwise submatched observations

In order to fit pairwise matched mixed-effects models, the experimenter should utilize the baseline submatch information to subtract corresponding control growth from its intervened counterpart. For this, a field indicating *Submatch* should be available in the data frame, and subtraction in a pairwise manner per each time point *Time*. For example:

```
> arndat <- arndat[order(arndat[,'Submatch']),]
> arnpair <- do.call('rbind', by(arndat, INDICES=arndat[,'Submatch'], FUN=function(z){
+         # Within each Submatch, subtract Vehicle from the Case
+         z[,'PairPSA'] <- z[,'PSA'] - z[z[,'Group']=='Vehicle','PSA']
+         z
+ }))
> arnpair[1:10,]

                  PSA DrugWeek    ID   Submatch   Group ARN MDV PairPSA
Submatch_1.11  21.30        0 ID003 Submatch_1 Vehicle   0   0    0.00
Submatch_1.12  45.69        1 ID003 Submatch_1 Vehicle   0   0    0.00
Submatch_1.13  54.50        2 ID003 Submatch_1 Vehicle   0   0    0.00
Submatch_1.14  53.55        3 ID003 Submatch_1 Vehicle   0   0    0.00
Submatch_1.15  27.64        4 ID003 Submatch_1 Vehicle   0   0    0.00
Submatch_1.266 23.62        0 ID040 Submatch_1     ARN   1   0    2.32
Submatch_1.267 22.09        1 ID040 Submatch_1     ARN   1   0  -23.60
Submatch_1.268 30.95        2 ID040 Submatch_1     ARN   1   0  -23.55
Submatch_1.269 31.98        3 ID040 Submatch_1     ARN   1   0  -21.57
Submatch_1.270 41.54        4 ID040 Submatch_1     ARN   1   0   13.90

> # The vehicle observations are redundant (subtracted from themselves)
> arnpair <- arnpair[arnpair[,'Group']=='ARN',]
```

In the above example, first pairwise computed PSA results in $23.62-21.30 = 2.32$ at time point $DrugWeek = 0$ (baseline). The following time points, i.e. $DrugWeek = 1$ result in turn a much more drastic growth in control tumor, i.e. $22.09 - 45.69 = -23.60$. In this particular example, the treated tumor seems to grow much slower for 3 weeks subsequently to the baseline, until it bounces back in the final time point.

4.3 Fitting conventional and pairwise matched mixed-effects models

Linear mixed-effects models compose of two main components:

- **Fixed effects**; Population effects, usually considered to cover either whole range of experimental units or a subpopulation such as an intervention group

- **Random effects**; Individual effects, that allows flexible model fits. Typical random effects include a random intercept (variation at baseline) and a random slope (individual variation in the growth coefficient).

The formula interface in R for fitting linear mixed-effects models in `lme4`-package consists of three parts:

$$LFS \sim \mathbf{Xb} + \mathbf{Zu} + \epsilon \tag{1}$$

where the LFS refers to left-hand side, i.e. the response vector $\mathbf{y}$ which is usually a tumor growth feature such as serum PSA or tumor volume. The right hand side from $\sim$ holds the fixed effects part (here $\mathbf{Xb}$), random effects part (here $\mathbf{Zu}$) and the normally distributed error term ϵ.

Fixed effects are separated using the + sign. A typical longitudinal preclinical model could be built on three fixed effects terms:

$$1 + Time + Time : Group \tag{2}$$

where 1 refers to a common intercept, which could be alternatively omitted using either a 0 or a -1 sign instead of 1. $Time$ typically is a running time point indicator, that starts from 0 at baseline and ranges to certain time units such as weeks or days, and tumor growth is computed a slope coefficient as a function of time. Furthermore, the term $Time : Group$ adds a binary indicator $Group$ that may be used to compare a subpopulation in comparison to control tumor growth.

Furthermore, random effects follow a notation:

$$\begin{cases} +(1 + Time|GroupingFactor) \\ +(1|GroupingFactor) + (0 + Time|GroupingFactor) \end{cases} \tag{3}$$

where the notation indicates that each unique factor value within variable `GroupingFactor` is treated as an instance of the experimental unit. The upper notation indicates that both an individual-specific intercept as well as an individualized time-dependent slope are estimated along with a cross-covariance between the two normally distributed random effects. The lower notation in turn estimates these two effects, an individual-specific intercept and an individualized time-dependent slope, separately from each other. By default we utilized the lower notation approach, though the upper notation may offer an interesting alternative. The error term does not need to explicitly included in the model formula. In the ARN-509 -study, the presented mixed-effects models were fitted using:

```
> fit_arn_unmatched <- lmer(PSA ~ 1 + DrugWeek + DrugWeek:ARN
+         + (1|ID) + (0 + DrugWeek|ID), data = arndat)
> fit_arn_matched <- lmer(PairPSA ~ 0 + DrugWeek
+         + (1|ID) + (0 + DrugWeek|ID), data = arnpair)
> summary(fit_arn_unmatched)
```

```
Linear mixed model fit by REML t-tests use Satterthwaite approximations to degrees
  of freedom [lmerMod]
Formula: PSA ~ 1 + DrugWeek + DrugWeek:ARN + (1 | ID) + (0 + DrugWeek |      ID)
   Data: arndat
```

```
REML criterion at convergence: 1082.6

Scaled residuals:
    Min      1Q  Median      3Q     Max
-3.4768 -0.3911 -0.0044  0.3425  3.1437

Random effects:
 Groups   Name        Variance Std.Dev.
 ID       (Intercept) 67.80    8.234
 ID.1     DrugWeek    26.65    5.163
 Residual             33.05    5.749
Number of obs: 150, groups:  ID, 30

Fixed effects:
             Estimate Std. Error     df t value Pr(>|t|)
(Intercept)    14.311      1.709 29.587   8.374 2.68e-09 ***
DrugWeek       10.062      1.407 28.206   7.150 8.47e-08 ***
DrugWeek:ARN   -7.627      1.982 27.792  -3.849 0.000636 ***
---
Signif. codes:  0 Ś***Š 0.001 Ś**Š 0.01 Ś*Š 0.05 Ś.Š 0.1 Ś Š 1

Correlation of Fixed Effects:
            (Intr) DrugWk
DrugWeek    -0.092
DrugWek:ARN  0.000 -0.704

> summary(fit_arn_matched)

Linear mixed model fit by REML t-tests use Satterthwaite approximations to degrees
  of freedom [lmerMod]
Formula: PairPSA ~ 0 + DrugWeek + (1 | ID) + (0 + DrugWeek | ID)
   Data: arnpair

REML criterion at convergence: 592.8

Scaled residuals:
     Min       1Q   Median       3Q      Max
-2.36132 -0.53405 -0.04075  0.34125  2.72586

Random effects:
 Groups   Name        Variance Std.Dev.
 ID       (Intercept) 49.74    7.053
 ID.1     DrugWeek    79.11    8.894
 Residual             70.55    8.399
Number of obs: 75, groups:  ID, 15

Fixed effects:
         Estimate Std. Error     df t value Pr(>|t|)
DrugWeek   -7.962      2.366 13.770  -3.365  0.00472 **
```

```
---
Signif. codes:  0 Ś***Š 0.001 Ś**Š 0.01 Ś*Š 0.05 Ś.Š 0.1 Ś Š 1
```

Alternate pairwise-matched longitudinal model formulations could include for example:

```
> # Paired model with correlated random effects
> fit_arn_matched2 <- lmer(PairPSA ~ 0 + DrugWeek
+         + (1 + DrugWeek|ID), data = arnpair)
> # Paired model with an intercept fixed effect
> fit_arn_matched3 <- lmer(PairPSA ~ 1 + DrugWeek
+         + (1|ID) + (0 + DrugWeek|ID), data = arnpair)
> # Paired model with an intercept fixed effect
> # and correlated random effects
> fit_arn_matched4 <- lmer(PairPSA ~ 1 + DrugWeek
+         + (1 + DrugWeek|ID), data = arnpair)
> # Only fixed effects shown here to save space
> summary(fit_arn_matched2)$coefficients

          Estimate Std. Error df   t value   Pr(>|t|)
DrugWeek -5.840363   2.122596 14 -2.751519 0.01559838

> summary(fit_arn_matched3)$coefficients

             Estimate Std. Error       df   t value    Pr(>|t|)
(Intercept) -4.305333   2.295298 14.28259 -1.875719 0.081283732
DrugWeek    -7.302533   2.423880 14.28259 -3.012745 0.009142255

> summary(fit_arn_matched4)$coefficients

             Estimate Std. Error       df   t value    Pr(>|t|)
(Intercept) -4.305333   2.122769 13.99982 -2.028169 0.062013340
DrugWeek    -7.302533   2.241686 13.99999 -3.257608 0.005725389
```

For fitted models, further functions are provided inside `hamlet`, both for visualization as well as diagnostics purposes. Examples:

- `hamlet::mem.plotresid` for plotting residuals along with trend lines
- `hamlet::mem.getcomp` for extracting a `data.frame` containing observation-specific fixed effects fit, full model fit, response vector etc. These can be then used to visualize the corresponding model fits, for example by paneling for experimental groups or each individual participating in the study.

R-vivo User Instructions

Introduction

R-vivo is a browser-based interface for the hamlet R-package (**Fig. 1**). Its functionalities are intended mainly for refining and improving the experimental design and statistical analysis of pre-clinical intervention studies, although the tools can be generalized to cover other relevant fields. R-vivo analysis functions are divided into two main subcategories:

- Pre-intervention analysis
- Post-intervention analysis (incl. power analysis)

Both subsections require specific data format(s). Pre-intervention analysis uses the so-called *wide format* data and post-intervention analysis uses the so-called *long format* data (see more detailed examples below).

File upload

R-vivo supports several different input file formats:

- Excel files: .xls .xlsx
- Comma separated: .csv
- Text files: .txt

R-vivo automatically detects the most suitable file format of the above templates. If your data is in the *long format*, press the "Data is in long format"-button after uploading the file. *Long format* data is suitable for downstream mixed-effects modelling, both for post-intervention statistical testing as well as for power analyses prior to conducting an actual experiment.

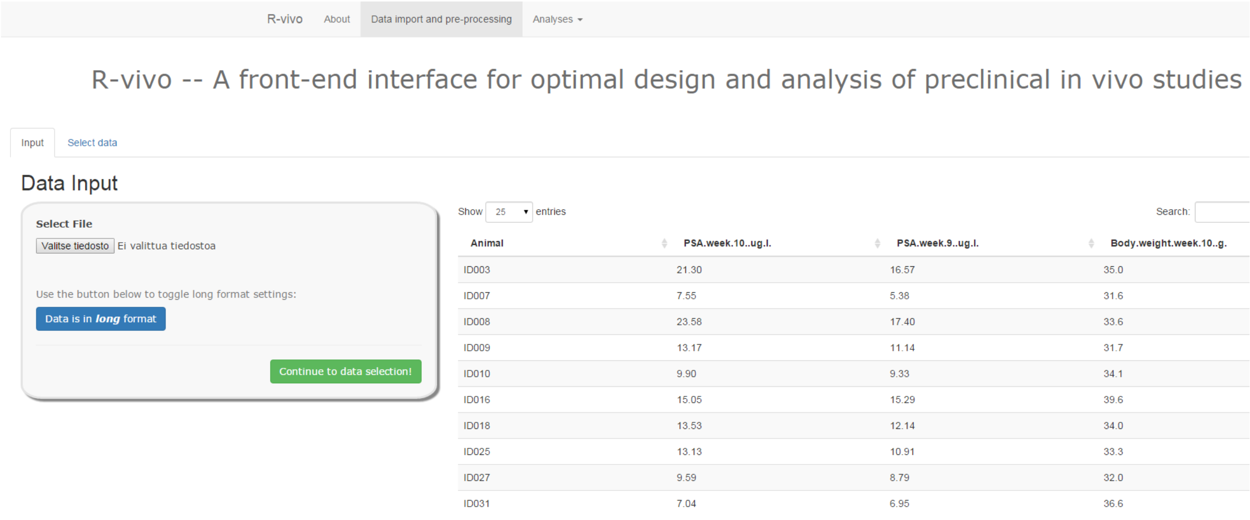

Figure 1: Overview of R-vivo.

	A	B	C	D	E	F	G	H	I	J
1	ID	Group	TreatmentInitiationWeek	Submatch	PSAWeek2	PSAWeek3	PSAWeek4	PSAWeek5	PSAWeek6	PSAWeek7
2	ID003	Vehicle	Week10	Submatch_1	7.67	14.76	24.78	2.03	5.97	8.16
3	ID007	MDV	Week10	Submatch_10	2.01	5.17	8.59	14.62	1.99	2.81
4	ID008	MDV	Week10	Submatch_3	6.68	15.17	22.85	1.96	5.69	9.2
5	ID009	Vehicle	Week10	Submatch_2	8.64	17.47	26.81	1.32	3.1	5.38
6	ID010	ARN	Week10	Submatch_11	1.73	3.93	6.18	11.83	1.75	3.92
7	ID016	Vehicle	Week10	Submatch_12	3.68	7.85	13.19	20.96	2.28	4.36
8	ID018	MDV	Week10	Submatch_11	4.69	7.19	12.85	24.08	2.36	5.39
9	ID025	MDV	Week10	Submatch_2	9.01	17.01	29.24	0.44	2.04	3.42
10	ID027	MDV	Week10	Submatch_5	3.3	7.27	15.83	0.04	1.61	2.74
11	ID031	ARN	Week10	Submatch_6	5.75	11.13	19.2	0.89	1.77	3.14
12	ID032	Vehicle	Week10	Submatch_6	7.3	11.79	20.36	0.63	1.51	2.61
13	ID037	Vehicle	Week10	Submatch_5	5.78	11.96	21.01	0.87	2.22	3.8

Figure 2: Example of *wide format* data, where rows correspond to individuals and columns to different animal characteristics at baseline.

In the *wide format* data (**Fig. 2**), the columns are indicators of the variables available for the experimental unit (here animal or tumor). The *wide format* is utilized for constructing dissimilarity matrices from the multivariate baseline data.

	A	B	C	D	E	F	G	H	I	J
1	log2PSA	BW	Submatch	ID	Week	DrugWeek	Group	Vehicle	ARN	MDV
2	4.4127815	35	Submatch_1	ID003	10	0	Vehicle	1	0	0
3	5.5138065	36.1	Submatch_1	ID003	11	1	Vehicle	1	0	0
4	5.7681843	37.9	Submatch_1	ID003	12	2	Vehicle	1	0	0
5	5.7428146	37.5	Submatch_1	ID003	13	3	Vehicle	1	0	0
6	4.7886857	39.7	Submatch_1	ID003	14	4	Vehicle	1	0	0
7	2.9164766	31.6	Submatch_10	ID007	10	0	MDV	0	0	1
8	3.2779847	31.7	Submatch_10	ID007	11	1	MDV	0	0	1
9	4.1251551	32.4	Submatch_10	ID007	12	2	MDV	0	0	1
10	4.5103290	33.5	Submatch_10	ID007	13	3	MDV	0	0	1

Figure 3: Example of *long format* data for post-intervention analysis.

In the *long format* data (**Fig. 3**) selected set of longitudinally interesting response variables are available (i.e. here PSA or body weight), and the different observations belonging to a single experimental unit (here animal) are uniquely distinguished using measurement time (field *Week* or *DrugWeek*) and/or identification codes (field *ID*). In this experiment, treatment is initiated at week 10 so *DrugWeek = 0* is the baseline for initiation of interventions. This *long format* is typically used for longitudinal mixed-effects modeling where multiple observations are correlated within an experimental unit.

In this particular example (**Fig. 4**), matching of animals is conducted before allocation to the treatment arms based on three characteristics: PSA level at baseline, PSA level at the week prior to allocation, and body weight at baseline.

	A	B	C	D
1	Animal	PSA week 10 [ug/l]	PSA week 9 [ug/l]	Body weight week 10 [g]
2	ID003	21,3	16,57	35
3	ID007	7,55	5,38	31,6
4	ID008	23,58	17,4	33,6
5	ID009	13,17	11,14	31,7
6	ID010	9,9	9,33	34,1
7	ID016	15,05	15,29	39,6
8	ID018	13,53	12,14	34
9	ID025	13,13	10,91	33,3
10	ID027	9,59	8,79	32
11	ID031	7,04	6,95	36,6

Figure 4: Example data further used in this document.

Non-bipartite multigroup matching of the example data

The multigroup matching presents the fundamental experimental design improvement presented in the current work. By identifying similar subgroups (*submatches*) of experimental units, the pipeline allows random distribution of these similar units evenly to the intervention arms. This leads to each group having a corresponding experimental unit in each of the other intervention arms, as well as results in balanced distributions over the treatment arms for the baseline characteristics. As the *submatches* consider all pairwise comparisons, the interventions arms can be blinded (for instance no need to fix a control group), and therefore comply with the rigorous standards applied e.g. in controlled clinical trials.

The pre-intervention analyses begin with uploading of *wide format* data (**Fig. 5**).

Step 1:

- Upload example.xlsx (**Fig. 5**)

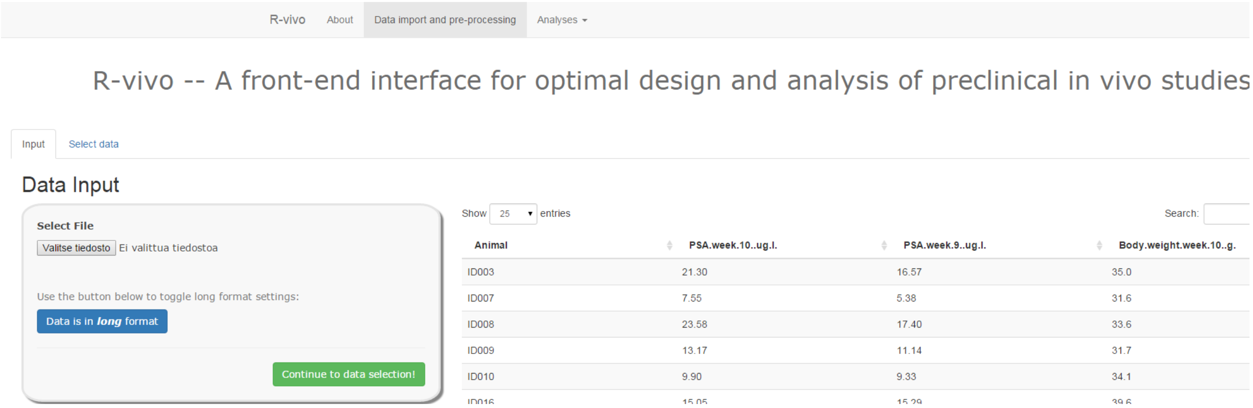

Figure 5: File upload example.

Step 2:

If unnecessary columns are present in the data, process the data accordingly (**Fig. 6**).

- Press the "Continue to data selection!"-button
- Select the desired columns
- Choose a representative name for the data
- Press "Save and continue!" to advance to the next step

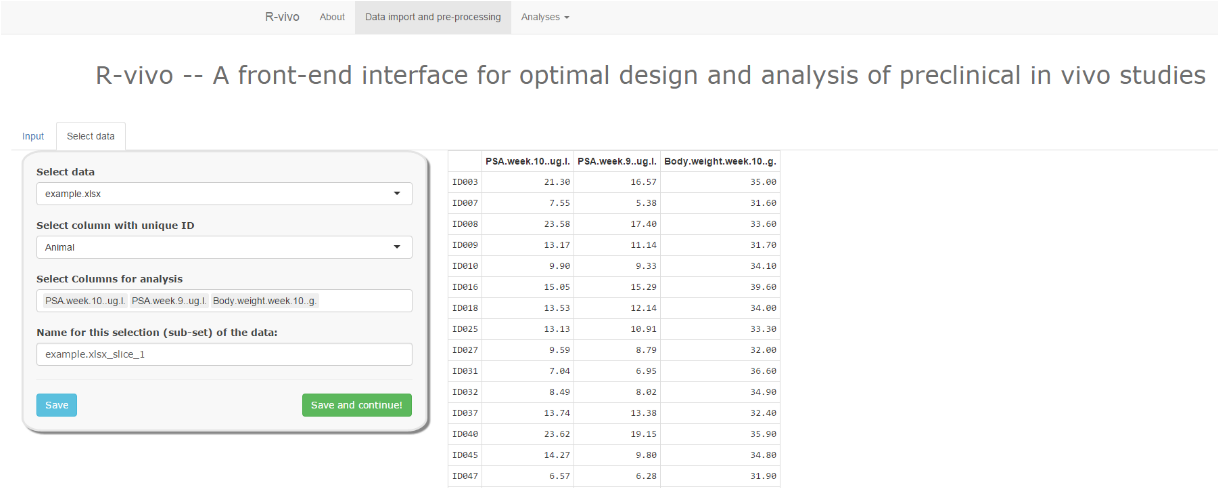

	PSA.week.10..ug.l.	PSA.week.9..ug.l.	Body.weight.week.10..g.
ID003	21.30	16.57	35.00
ID007	7.55	5.38	31.60
ID008	23.58	17.40	33.60
ID009	13.17	11.14	31.70
ID010	9.90	9.33	34.10
ID016	15.05	15.29	39.60
ID018	13.53	12.14	34.00
ID025	13.13	10.91	33.30
ID027	9.59	8.79	32.00
ID031	7.04	6.95	36.60
ID032	8.49	8.02	34.90
ID037	13.74	13.38	32.40
ID040	23.62	19.15	35.90
ID045	14.27	9.80	34.80
ID047	6.57	6.28	31.90

Figure 6: Column selection.

Step 3: Distance and dissimilarity functions

A distance or dissimilarity function is used to describe the amount of dissimilarity between two experimental units (animal or tumours), based on their baseline characteristics. Common choices include: Euclidean distance, standardized Euclidean distance, Manhattan distance, and Gower's dissimilarity for mixed type data. (**Fig. 7**)

- Select the desired subset of data
- Select the metric for computing the dissimilarity matrix
- If you want to standardize the Euclidean or Manhattan distances, check "Standardize measurements" box (z-score transformation)
- Press "Save and continue!"-button to advance to the next step

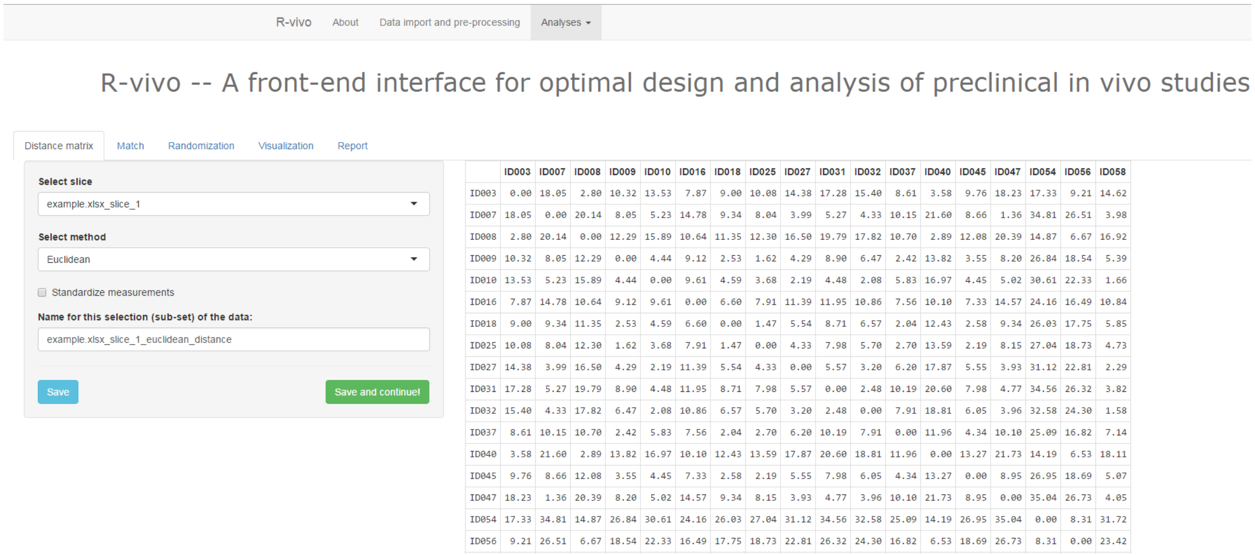

	ID003	ID007	ID008	ID009	ID010	ID016	ID018	ID025	ID027	ID031	ID032	ID037	ID040	ID045	ID047	ID054	ID056	ID058
ID003	0.00	18.05	2.80	10.32	13.53	7.87	9.00	10.08	14.38	17.28	15.40	8.61	3.58	9.76	18.23	17.33	9.21	14.62
ID007	18.05	0.00	20.14	8.05	5.23	14.78	9.34	8.04	3.99	5.27	4.33	10.15	21.60	8.66	1.36	34.81	26.51	3.98
ID008	2.80	20.14	0.00	12.29	15.89	10.64	11.35	12.30	16.50	19.79	17.82	10.70	2.89	12.08	20.39	14.87	6.67	16.92
ID009	10.32	8.05	12.29	0.00	4.44	9.12	2.53	1.62	4.29	8.90	6.47	2.42	13.82	3.55	8.20	26.84	18.54	5.39
ID010	13.53	5.23	15.89	4.44	0.00	9.61	4.59	3.68	2.19	4.48	2.08	5.83	16.97	4.45	5.02	30.61	22.33	1.66
ID016	7.87	14.78	10.64	9.12	9.61	0.00	6.60	7.91	11.39	11.95	10.86	7.56	10.10	7.33	14.57	24.16	16.49	10.84
ID018	9.00	9.34	11.35	2.53	4.59	6.60	0.00	1.47	5.54	8.71	6.57	2.04	12.43	2.58	9.34	26.03	17.75	5.85
ID025	10.08	8.04	12.30	1.62	3.68	7.91	1.47	0.00	4.33	7.98	5.70	2.70	13.59	2.19	8.15	27.04	18.73	4.73
ID027	14.38	3.99	16.50	4.29	2.19	11.39	5.54	4.33	0.00	5.57	3.20	6.20	17.87	5.55	3.93	31.12	22.81	2.29
ID031	17.28	5.27	19.79	8.90	4.48	11.95	8.71	7.98	5.57	0.00	2.48	10.19	20.60	7.98	4.77	34.56	26.32	3.82
ID032	15.40	4.33	17.82	6.47	2.08	10.86	6.57	5.70	3.20	2.48	0.00	7.91	18.81	6.05	3.96	32.58	24.30	1.58
ID037	8.61	10.15	10.70	2.42	5.83	7.56	2.04	2.70	6.20	10.19	7.91	0.00	11.96	4.34	10.10	25.09	16.82	7.14
ID040	3.58	21.60	2.89	13.82	16.97	10.10	12.43	13.59	17.87	20.60	18.81	11.96	0.00	13.27	21.73	14.19	6.53	18.11
ID045	9.76	8.66	12.08	3.55	4.45	7.33	2.58	2.19	5.55	7.98	6.05	4.34	13.27	0.00	8.95	26.95	18.69	5.07
ID047	18.23	1.36	20.39	8.20	5.02	14.57	9.34	8.15	3.93	4.77	3.96	10.10	21.73	8.95	0.00	35.04	26.73	4.05
ID054	17.33	34.81	14.87	26.84	30.61	24.16	26.03	27.04	31.12	34.56	32.58	25.09	14.19	26.95	35.04	0.00	8.31	31.72
ID056	9.21	26.51	6.67	18.54	22.33	16.49	17.75	18.73	22.81	26.32	24.30	16.82	6.53	18.69	26.73	8.31	0.00	23.42

Figure 7: Distance/dissimilarity matrix construction.

Step 4: Matching

The non-bipartite optimal matching problem can be solved using the implemented Genetic Algorithm (GA). The algorithm takes as input a distance matrix and desired size of the submatches (i.e. number of animals/tumours per a subgroup, and thus per intervention group). If the size of the submatch is for example $G = 3$, it indicates that the GA minimizes dissimilarity edges within triplets. These subgroups of desired size are called *submatches*. (**Fig. 8**)

- Select the desired distance matrix
- Select the submatch size G, the count of desired intervention arms
- Press the "Match"-button
- Default options are suitable for small to mediocre size experiments, but a better solution may be found by checking "Advanced options" and tuning the GA parameters. Notice that parameters such as *generations* will result in a linear increase in the GA run time.
- Press "Save and continue!"-button to advance to the next step

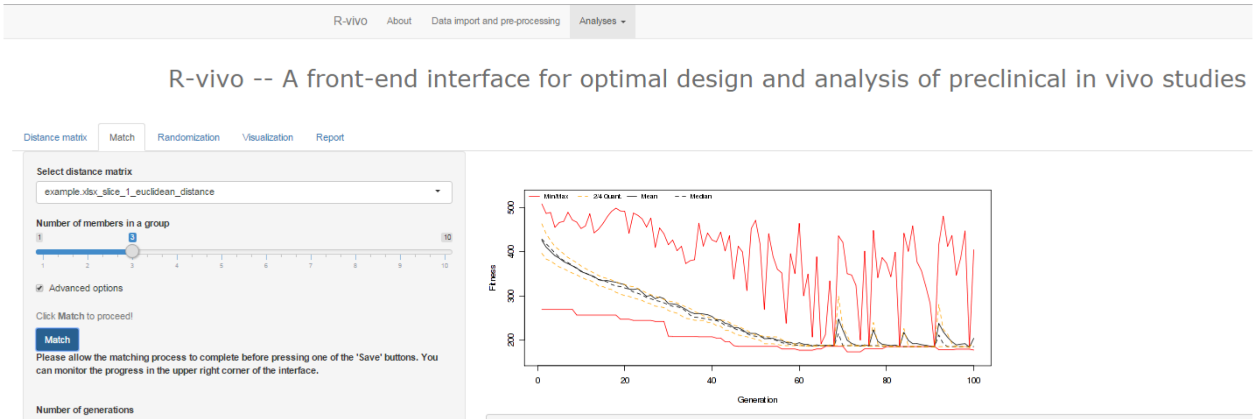

	PSA.week.10..ug.l.	PSA.week.9..ug.l.	Body.weight.week.10..g.	Submatch
ID003	21.30	16.57	35.00	Submatch_3
ID007	7.55	5.38	31.60	Submatch_2
ID008	23.58	17.40	33.60	Submatch_6
ID009	13.17	11.14	31.70	Submatch_5
ID010	9.90	9.33	34.10	Submatch_4
ID016	15.05	15.29	39.60	Submatch_3
ID018	13.53	12.14	34.00	Submatch_4
ID025	13.13	10.91	33.30	Submatch_5
ID027	9.59	8.79	32.00	Submatch_2
ID031	7.04	6.95	36.60	Submatch_1
ID032	8.49	8.02	34.90	Submatch_1
ID037	13.74	13.38	32.40	Submatch_5
ID040	23.62	19.15	35.90	Submatch_3
ID045	14.27	9.80	34.80	Submatch_4
ID047	6.57	6.28	31.90	Submatch_2
ID054	34.72	27.14	32.10	Submatch_6
ID056	28.15	22.05	32.20	Submatch_6
ID058	9.74	7.68	34.00	Submatch_1

Figure 8: Non-bipartite multigroup matching example.

Step 5: Randomization based on submatched individuals

The intervention groups are obtained by dividing the members of each optimal submatch randomly into the various separate treatment arm. Since all the pairwise within-submatch distances are minimized, this guarantees that comparable individuals are randomly divided into separate arms and that the experiment may be blinded in respect to the future true intervention labels (**Fig. 9**). These dummy-labels $A, B, C, \ldots$ may be given as-is to an external experimenter to ensure that the randomizer has not been influenced by possible intervention choices.

Steps

- Select matched data for randomization
- Press “Randomise”
- Press “Continute to visualization” to analyze your results
- Press “Print” in upper right corner of data table to print your results
- Press “Copy” to copy data to clipboard
- Use the “text import wizard” for pasting the results to Excel. Otherwise Excel may transfer some columns to wrong type automatically.
- Press “Save” to save table as a .csv or .xls file

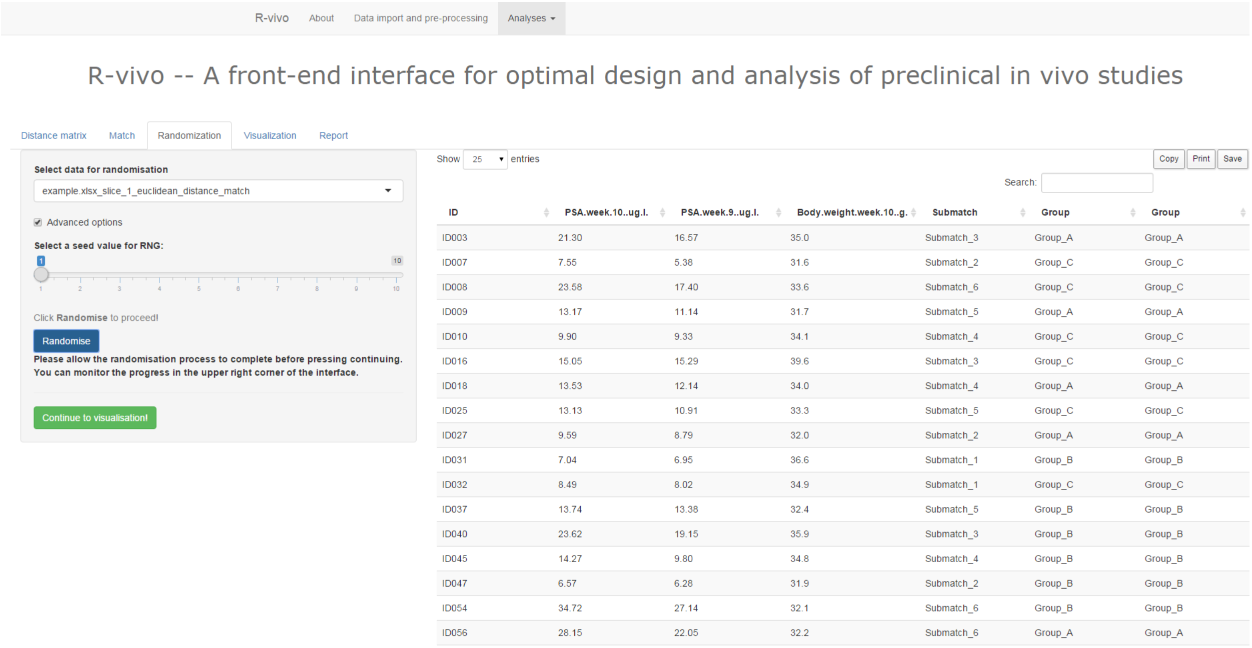

Figure 9: Randomized allocation based on the identified submatches. Each submatch is evenly distributed to the (blinded) treatment arms.

Visualizations for pre-clinical data:

Various visualization methods are available to illustrate the baseline balancing and submatches. For example, the boxplots in respect to allocation groups can be plotted using the boxplot tab.

Boxplot

- Go to the "Pre-intervention/Visualization/Boxplot" tab
- Select the relevant analyzed data
- Select the response variable of interest
- Select a grouping variable (e.g. *Submatch* or *Group*)
- Press "Plot" (**Fig. 10**)
- Press "Advanced options" if you want to add annotations
- Press "Download" to download a pdf file of the boxplot

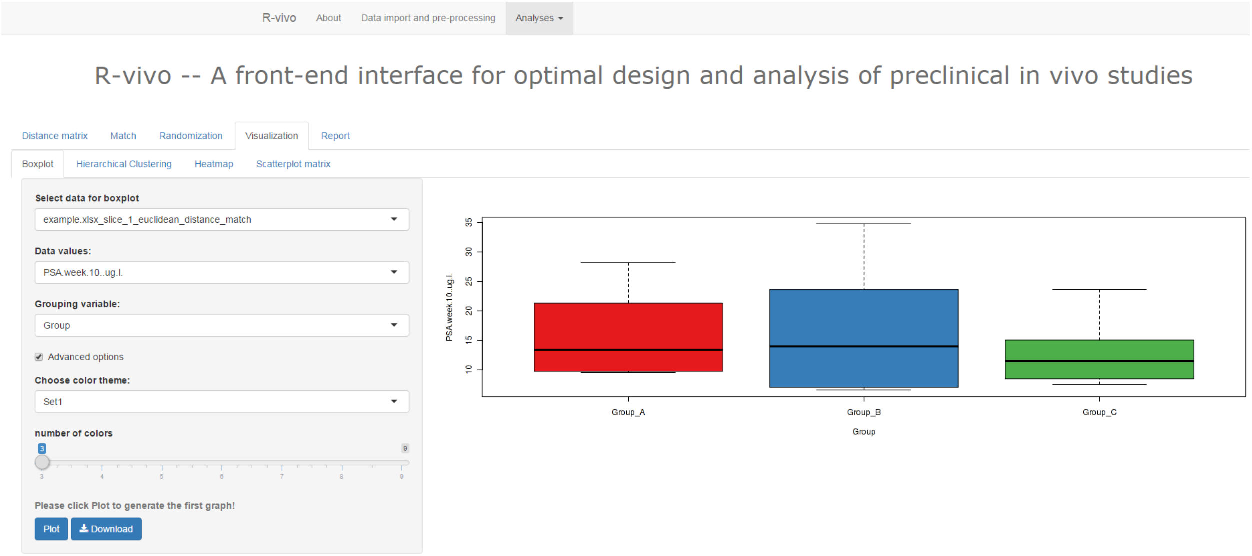

Figure 10: Boxplots of the presented data.

Hierarchical Clustering:

- Go to “Hierarchical Clustering”-tab
- Select the desired dissimilarity matrix to plot (**Fig. 11**)
- Press “Download” to get a PDF file of the clustering

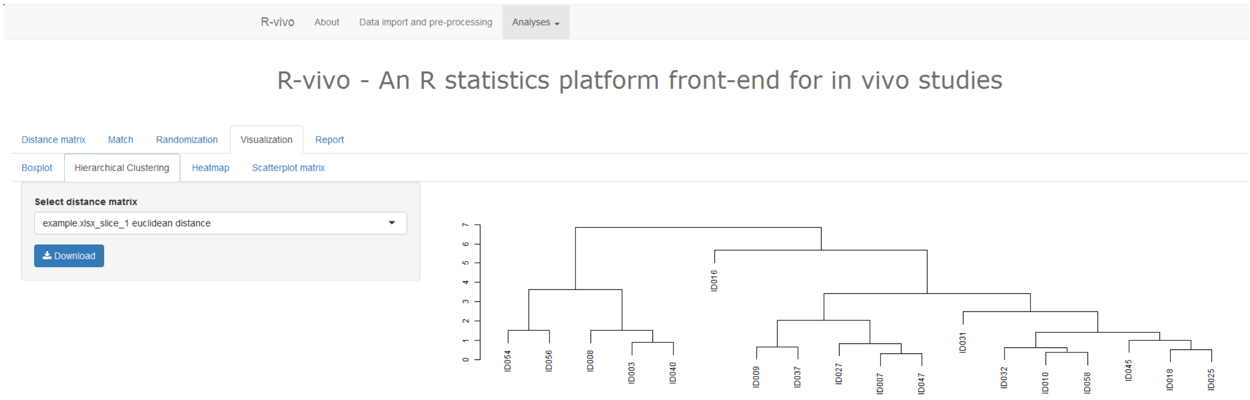

Figure 11: Hierarchical clustering of the presented data.

Heatmap:

A common way to illustrate distance matrices is through heatmaps, along with hierarchical clustering to connect similar individuals. R-vivo utilises the d3heatmap package (https://github.com/rstudio/d3heatmap) for generating interactive heatmaps.

Steps:

- Go to "Heatmap"-tab
- Select the desired dissimilarity matrix (**Fig. 12**)
- Press "Advanced options" if specific annotations are needed.
- Press "Download" to get an interactive picture of the heatmap.

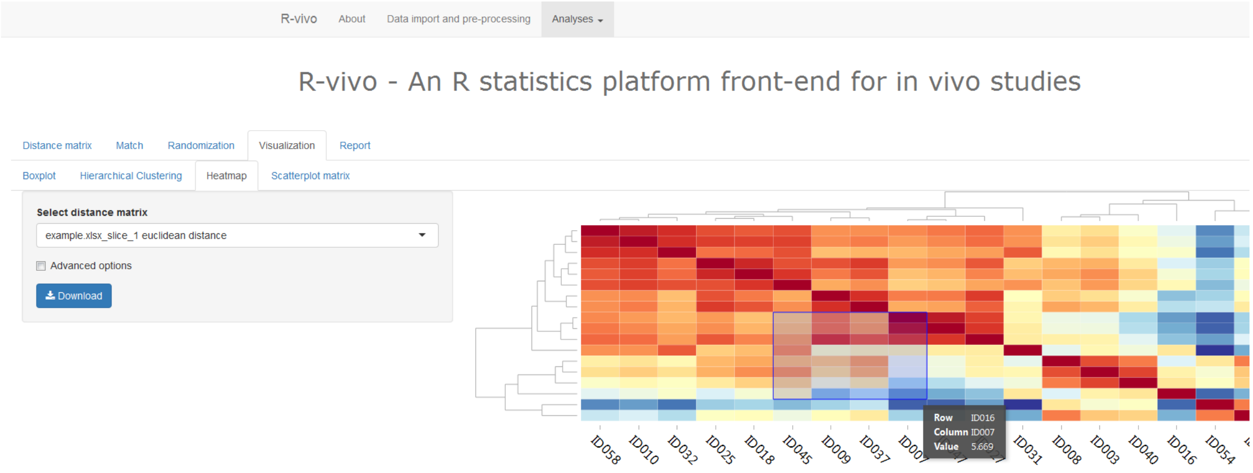

Figure 12: Heatmap presentation of the computed distance matrix

Scatterplot matrices

Scatterplot matrices can be used to determine roughly if you have a linear correlation between multiple variables, or to identify other interesting pairwise trends between the variables in the data.

Steps:

- Go to “Scatterplotmatrix”-tab
- Select variables for the plots (**Fig. 13**)
- Press “Download” to get a PDF file of the scatterplot matrix

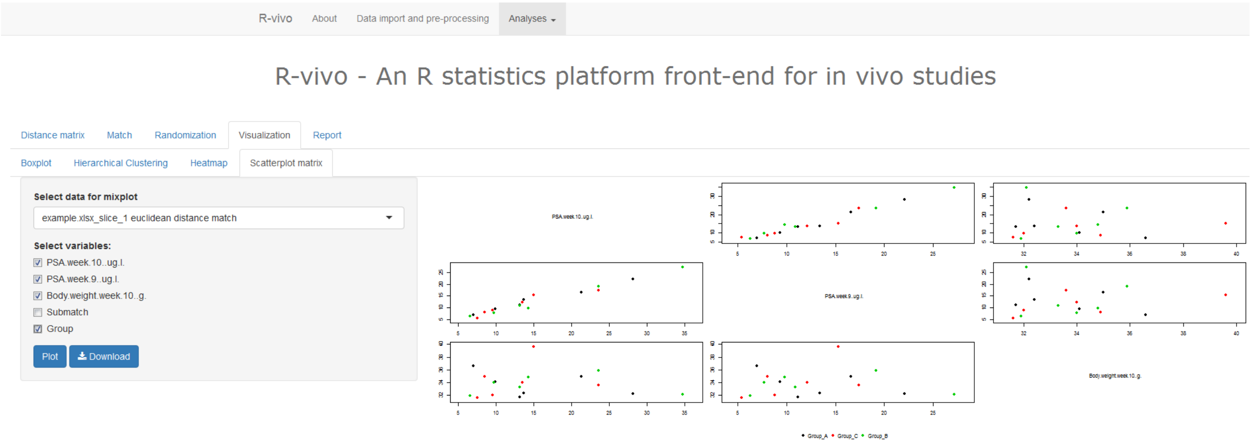

Figure 13: Multivariate scatterplot of the baseline data.

Post-intervention

Post-intervention refers to the modeling of the provided *long format* data, here using mixed-effects modeling. It may be utilized to fit a mixed-effects model and to test population-wise hypotheses (fixed effects) and to model the individual variation (random effects).

Power simulation here is done through bootstrapping (sampling with replacement) the data source of a pre-fitted mixed-effects model, and then re-fitting and estimating the fixed effects coefficients to the bootstrapped datasets. However, please note that the power analysis is not intended to be used for retrospective speculation of an already conducted experiment. Its purpose is to model and simulate either an artificial or a pilot experiment, in order to improve accuracy and to guarantee sufficient statistical power in a representative future study.

Upload data

- Upload *long format* data
- Press the "Data is in long format"-button (**Fig. 14**)
- Press the "Continue to Post-intervention analysis!"-button to advance to the next step

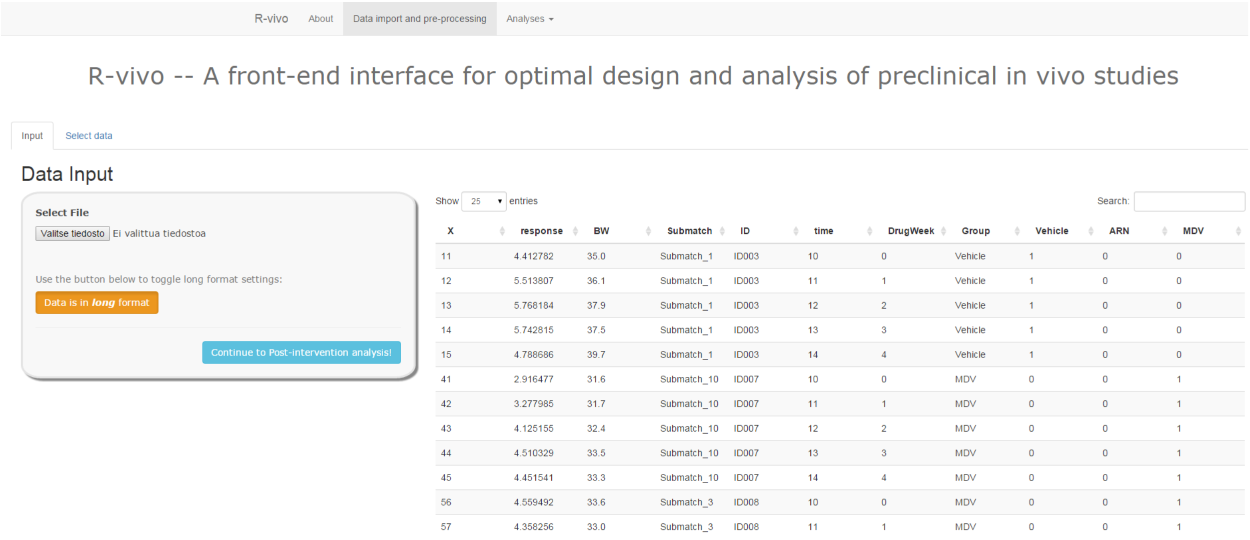

Figure 14: Long format data input for mixed-effects modeling.

Mixed-effects modeling

Mixed-effects models are a flexible model family that incorporate fixed effects (population effects) as well as random effects (individualized effects) into a regression model. By default, R-vivo utilizes linear mixed-effects models. Refer to the hamlet R-package step-by-step documentation or an extensive mixed-effects modeling resource (for example Bates et al.), in order to understand the details in the chosen model structure and its effect on the inference and conclusions.

To choose a suitable model:

- Select the desired *long format* data frame to model
- Select appropriate fixed effects structure
- Select appropriate random effects structure
- Select ID column (identification codes for the experimental unit)
- Select response column (primary response that will be tested, i.e. PSA or tumor volume)
- Select time column (longitudinal time column)
- Select intervention group (a binary indicator column for an intervention group)
- Select control group (a binary indicator column for the reference group)
- Press "Fit"
- In the "Summary"-tab, you can see a summary of the analysis (**Fig. 15**)
- Residual and longitudinal fit plots may be drawn in the corresponding tabs

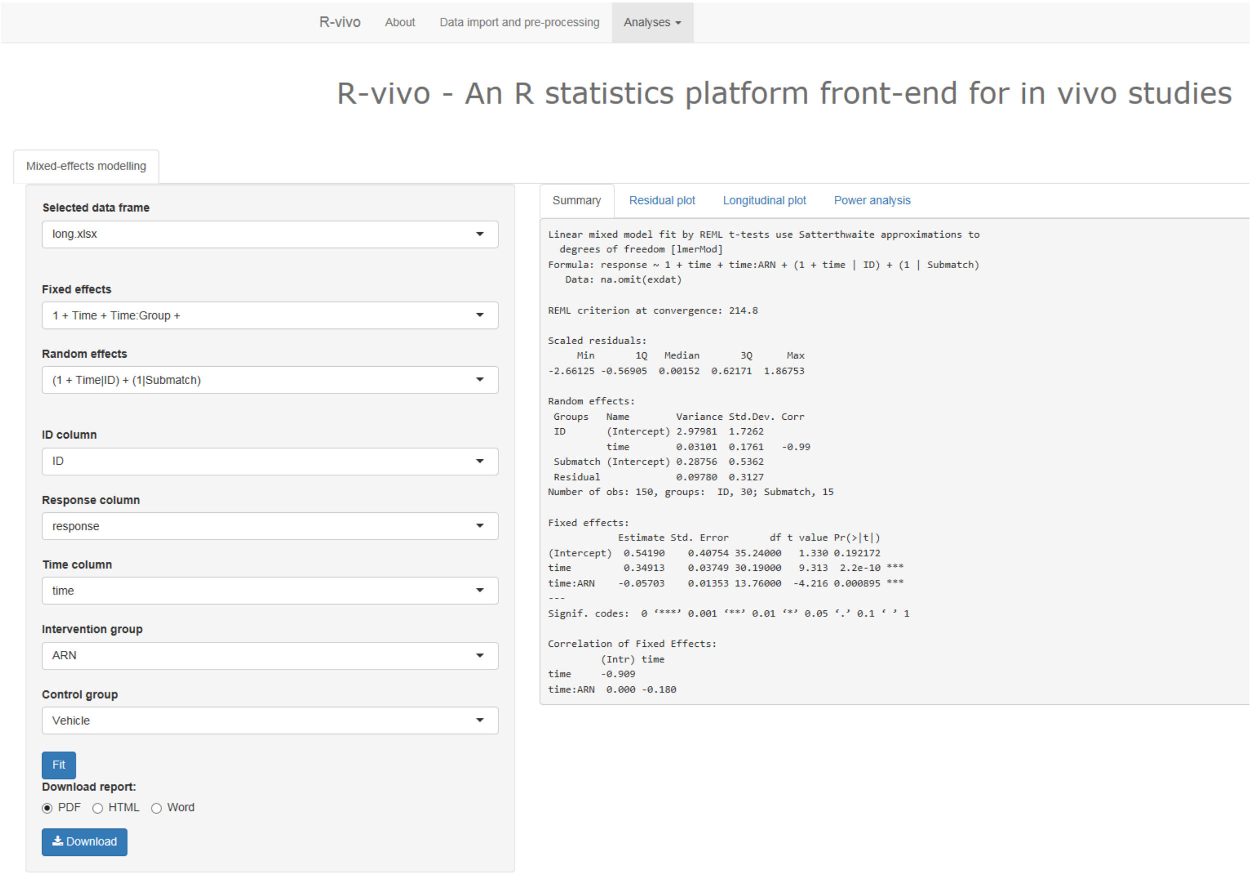

Figure 15: A fitted mixed-effects model and its summary.

Power analysis

The power analyses are sensitive to the model structure, and in order to obtain a smooth and accurate estimation of statistical power, the user should use a sufficient amount of bootstrapped datasets. Additionally, a tight grid of N values may ensure that exact numbers are reported. Here N refers to tested group-wise amounts of animals. It is a vector with a minimum value, maximum value, and a grid-spacing; for example, an N vector with minimum 5 and maximum 14 with a spacing of 3 would test $N = \{5, 8, 11, 14\}$.

- After fitting a mixed-effect model as described above, go to the “Power analysis”-tab
- Select a suitable sequence of N values to test
- Select number of bootstraps per Note: calculation time increases linearly
- If model contains grouping information check corresponding checkbox and select tested group.
- If a loess smoothed curve is desired for approximation purposes, choose the “Smoothed curve”-checkbox
- Each differently colored line represents the power of a fixed effects coefficient as a function of the sample size N (**Fig. 16**).
- Download a report of the power simulations by pressing “Download”

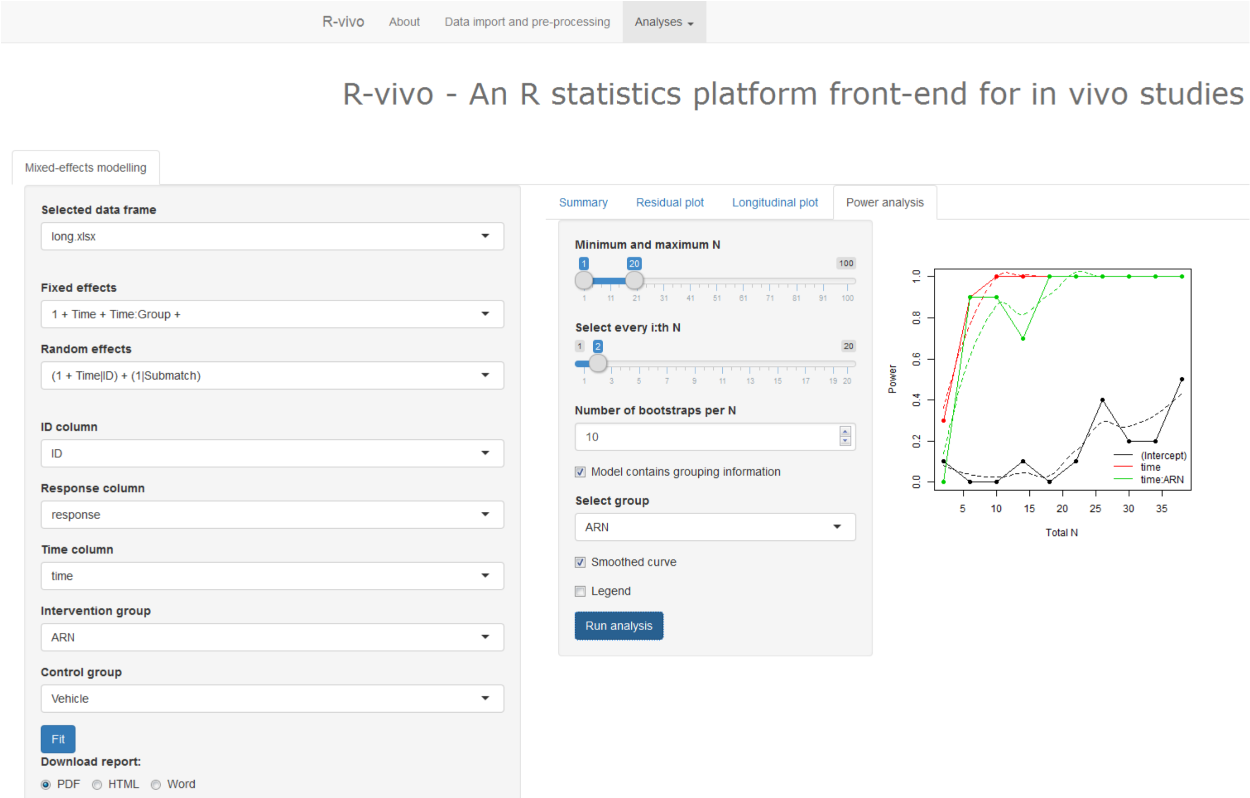

Figure 16: A bootstrapped power curve for each of the specified fixed effects, which utilizes the previously fitted mixed-effects model.

The ARRIVE Guidelines Checklist adopted from Kilkenny *et al.*[1]

*Animal Research: Reporting In Vivo Experiments**

**Section**	**#**	**RECOMMENDATION**	**ARN-509/MDV3100-study**	**ORX/ORX+Tx-study**
Title	1	Provide as accurate and concise a description of the content of the article as possible.	See Knuuttila et al[3] for the original publication.	Previously unpublished study.
Abstract	2	Provide an accurate summary of the background, research objectives, including details of the species or strain of animal used, key methods, principal findings and conclusions of the study.	See Knuuttila et al[3] for the original publication. Applicable fields for the methodological publication are described in this novel publication.	Previously unpublished study. Applicable fields for the methodological publication are described in this novel publication.
INTRODUCTION				
Background	3	**a.** Include sufficient scientific background (including relevant references to previous work) to understand the motivation and context for the study, and explain the experimental approach and rationale.<br>**b.** Explain how and why the animal species and model being used can address the scientific objectives and, where appropriate, the studys relevance to human biology.	See Knuuttila et al[3] for specific details.	The related background to orchidectomy (ORX) is well known in literature, while the undisclosed intervention (Tx) remains to be further described.
Objectives	4	Clearly describe the primary and any secondary objectives of the study, or specific hypotheses being tested.	To longitudinally model response to antiandrogen therapy for ARN-509 and MDV3100 in orthotopic VCaP cells	To longitudinally model response to orchidectomy (ORX) as well as a novel therapeutic intervention (Tx) in subcutaneous VCaP cells.
METHODS				
Ethical statement	5	Indicate the nature of the ethical review permissions, relevant licences (e.g. Animal [Scientific Procedures] Act 1986), and national or institutional guidelines for the care and use of animals, that cover the research.	All animal handling was conducted in accordance with Finnish Animal Ethics Committee and institutional animal care policies, which fully meet the requirements defined in current NIH guidelines on animal experimentation (license number 1993/04.10.03/2011).	The study was conducted in accordance with the Animal Experiment Board in Finland (ELLA) for the care and use of animals under the license ESAVI/7472/04.10.03/2012.
Study design	6	For each experiment, give brief details of the study design including:<br>**a.** The number of experimental and control groups.<br>**b.** Any steps taken to minimise the effects of subjective bias when allocating animals to treatment (e.g. randomisation procedure) and when assessing results (e.g. if done, describe who was blinded and when).<br>**c.** The experimental unit (e.g. a single animal, group or cage of animals). A time-line diagram or flow chart can be useful to illustrate how complex study designs were carried out.	120 mice were originally used for the study. Eventually 45 mice were used to form 3 study groups, one control group and two treatment groups, each including 15 mice. (**Supplementary Fig. S1b**). Experimental unit was a single animal. Blinding, allocation, and randomization were conducted as presented in this manuscript.	Originally 109 mice were allocated to 6 experimental groups, each containing 14 to 16 mice (**Supplementary Fig. S1c**). Experimental unit was a single animal. Blinding, allocation, and randomization were conducted as presented in this manuscript.
Experimental procedures	7	For each experiment and each experimental group, including controls, provide precise details of all procedures carried out. E.g.:<br>**a.** How (e.g. drug formulation and dose, site and route of administration, anaesthesia and analgesia used [including monitoring], surgical procedure, method of euthanasia). Provide details of any specialist equipment used, including supplier(s).<br>**b.** When (e.g. time of day).<br>**c.** Where (e.g. home cage, laboratory, water maze).<br>**d.** Why (e.g. rationale for choice of specific anaesthetic, route of administration, drug dose used).	Inoculations, blood sampling, castration: see Knuuttila et al[3]. Animals were treated with 20 mg/kg/day of treatment compounds. Compounds were administrated per os by gavage in the, every morning for 28 days at the animal laboratory. No anaesthetics were need for administration.	Detailed description is given in the Supplementary Material, section 'Subcutaneous VCaP xenograft studies in immunodeficient mice: Interventions by ORX and ORX+Tx' paragraphs 1 and 2.

*Original checklist published in *PLoS Biol*, June 2010.

Experimental animals	8	**a.** Provide details of the animals used, including species, strain, sex, developmental stage (e.g. mean or median age plus age range) and weight (e.g. mean or median weight plus weight range).<br>**b.** Provide further relevant information such as the source of animals, international strain nomenclature, genetic modification status (e.g. knock-out or transgenic), genotype, health/immune status, drug or test nave, previous procedures, etc.	Adult male immunodeficient mice (HSD:Athymice Nude Foxn1nu) were 6-7 weeks old, mean weight 29.8 g; sD 2.4, in the beginning of the experiment. Mice were purchased from Harlan Laboratories (Indianapolis,IN)	Detailed description is given in the Supplementary Material, section 'Subcutaneous VCaP xenograft studies in immunodeficient mice: Interventions by ORX and ORX+Tx' paragraph 1.
Housing and husbandry	9	Provide details of:<br>**a.** Housing (type of facility e.g. specific pathogen free [SPF]; type of cage or housing; bedding material; number of cage companions; tank shape and material etc. for fish).<br>**b.** Husbandry conditions (e.g. breeding programme, light/dark cycle, temperature, quality of water etc for fish, type of food, access to food and water, environmental enrichment).<br>**c.** Welfare-related assessments and interventions that were carried out prior to, during, or after the experiment.	See Knuuttila et al[3]: Mice were housed in individually ventilated cages under controlled conditions of light, temperature, and humidity. The mice were given irradiated soy-free natural-ingredient feed [RM3 (E); Special Diets Services, Witham, UK] and autoclaved tap water ad libitum, and were housed in specific pathogen-free conditions at the Central Animal Laboratory (University of Turku) in compliance with international guidelines.	Detailed description is given in the Supplementary Material, section 'Subcutaneous VCaP xenograft studies in immunodeficient mice: Interventions by ORX and ORX+Tx' paragraph 1.
Sample size	10	**a.** Specify the total number of animals used in each experiment, and the number of animals in each experimental group.<br>**b.** Explain how the number of animals was arrived at. Provide details of any sample size calculation used.<br>**c.** Indicate the number of independent replications of each experiment, if relevant.	120 mice were originally used for the study. Because of several different operations (inoculations of cells, castration, relapse to castration-resistant stage) prior to treatments, the amount of animals had to be significantly larger than what was needed for treatments (n=45).	The median group size was 15 animals, estimated on prior experience[3] and expected effect sizes from corresponding biological literature. Few groups were given larger size, due to suspected ethical constraints or adverse events (**Supplementary Fig. S1c**).
Allocating animals	11	**a.** Give full details of how animals were allocated to experimental groups, including randomisation or matching if done.<br>**b.** Describe the order in which the animals in the different experimental groups were treated and assessed.	See the main manuscript presenting the novel methodology[4].	See the main manuscript presenting the novel methodology[4].
Experimental outcomes	12	Clearly define the primary and secondary experimental outcomes assessed (e.g. cell death, molecular markers, behavioural changes).	Primary outcome: serum PSA concentration	Primary outcome was serum PSA, with secondary measurements on body weight and palpated tumor volume.
Statistical methods	13	**a.** Provide details of the statistical methods used for each analysis.<br>**b.** Specify the unit of analysis for each dataset (e.g. single animal, group of animals, single neuron).<br>**c.** Describe any methods used to assess whether the data met the assumptions of the statistical approach.	See the main manuscript presenting the novel methodology[4].	See the main manuscript presenting the novel methodology[4].
RESULTS				
Baseline data	14	For each experimental group, report relevant characteristics and health status of animals (e.g. weight, microbiological status, and drug or test nave) prior to treatment or testing. (This information can often be tabulated).	Serum PSA level, the change of the PSA level, body weights, cage placement, the week at which castration took place.	Baseline PSA levels, relative changes to previous measurement, body weights, as well as the longest palpated tumor dimension were measured and balanced at baseline.
Numbers analysed	15	**a.** Report the number of animals in each group included in each analysis. Report absolute numbers (e.g. 10/20, not 50%2).<br>**b.** If any animals or data were not included in the analysis, explain why.	All mice that were included in treatment groups were included in the analysis (except one mouse that died unexpectedly for unknown reason during the treatment period).	All the allocated mice were included in the analyses, while right-censoring occurred for some intact tumor mice (**Supplementary Fig. S6a**).

Outcomes and estimation	16	Report the results for each analysis carried out, with a measure of precision (e.g. standard error or confidence interval).	See Knuuttila et al[3], as well as reported model outcomes in **Supplementary Fig. S5** and Table 1.	See the reported model outcomes in **Supplementary Fig. S6** and Table 1.
Adverse events	17	**a.** Give details of all important adverse events in each experimental group. **b.** Describe any modifications to the experimental protocols made to reduce adverse events.	No adverse effects were found with these compound treatments.	Some animals were sacrificed due to ethical tumor constraints (**Supplementary Fig. 6a**), but no significant adverse effects were observed.
DISCUSSION				
Interpretation/implications	18	**a.** Interpret the results, taking into account the study objectives and hypotheses, current theory and other relevant studies in the literature.<br>**b.** Comment on the study limitations including any potential sources of bias, any limitations of the animal model, and the imprecision associated with the results[2].<br>**c.** Describe any implications of your experimental methods or findings for the replacement, refinement or reduction (the 3Rs) of the use of animals in research.	The readily established antiandrogen drugs ARN-509 and MDV3100 were correctly identified to inhibit tumor growth. The mouse model, which is described in detail in Knuuttila et al.[3], represents a clinically relevant view to CRPC. The 3R principles were taken into account, as the novel mathematical methodology was together developed and adopted.	The ORX surgery treatment resulted in a drastic decrease in tumor growth, as expected, while the undisclosed treatment (Tx) remains to be further discussed, along with the specific details of the mouse model beyond those presented in our Supplementary Methods. The 3R principles were taken into account, as the novel mathematical methodology was together developed and adopted.
Generalisability/translation	19	Comment on whether, and how, the findings of this study are likely to translate to other species or systems, including any relevance to human biology.	The androgen receptor signalling (AR) remains similar to the clinical disease, and thus the mouse model represents relevant characteristics of CRPC.	While ORX does not represent a clinically feasible intervention, it resulted in a similar effect as in human. The effects of Tx remain to be discussed in future work.
Funding	20	List all funding sources (including grant number) and the role of the funder(s) in the study.	See funding statement in Knuuttila et al[3].	See funding statement in the manuscript[4].

References (ARRIVE Note)

1. Kilkenny, C., Browne, W.J., Cuthill, I.C., Emerson, M. & Altman, D.G. Improving bioscience research reporting: The ARRIVE guidelines for reporting animal research. *PLoS Biol* **8**: e1000412 (2010). doi:10.1371/journal.pbio.1000412

2. Schulz, K.F., Altman, D.G., Moher, D. & the CONSORT Group. CONSORT 2010 Statement: updated guidelines for reporting parallel group randomised trials. *BMJ* **340**: c332 (2010).

3. Knuuttila, M. *et al.* Castration induces upregulation of intratumoral androgen biosynthesis and androgen receptor expression in orthotopic VCaP human prostate cancer xenograft model. *Am J Pathol* **184**:2163-73 (2014). doi 10.1016/j.ajpath.2014.04.010

4. Laajala, T.D. *et al.* Optimized design and analysis of preclinical intervention studies *in vivo*. *Submitted* (2016).
